# Heterolytic Si−H Bond Cleavage at a Molybdenum‐Oxido‐Based Lewis Pair

**DOI:** 10.1002/chem.201800226

**Published:** 2018-04-27

**Authors:** Niklas Zwettler, Simon P. Walg, Ferdinand Belaj, Nadia C. Mösch‐Zanetti

**Affiliations:** ^1^ Institute of Chemistry, Inorganic Chemistry University of Graz Schubertstrasse 1 8010 Graz Austria

**Keywords:** hydrosilylation, Lewis pairs, metal oxido, molybdenum, silicon

## Abstract

The reaction of a molybdenum(VI) oxido imido complex with the strong Lewis acid B(C_6_F_5_)_3_ gave access to the Lewis adduct [Mo{OB(C_6_F_5_)_3_}(N*t*Bu)L_2_] featuring reversible B−O bonding in solution. The resulting frustrated Lewis pair (FLP)‐like reactivity is reflected by the compound's ability to heterolytically cleave Si−H bonds, leading to a clean formation of the novel cationic Mo^VI^ species **3 a** (R=Et) and **3 b** (R=Ph) of the general formula [Mo(OSiR_3_)(N*t*Bu)L_2_][HB(C_6_F_5_)_3_]. These compounds possess properties highly unusual for molybdenum d^0^ species such as an intensive, charge‐transfer‐based color as well as a reversible redox couple at very low potentials, both dependent on the silane used. Single‐crystal X‐ray diffraction analyses of **2** and **4 b**, a derivative of **3 b** featuring the [FB(C_6_F_5_)_3_]^−^ anion, picture the stepwise elongation of the Mo=O bond, leading to a large increase in the electrophilicity of the metal center. The reaction of **3 a** and **3 b** with benzaldehyde allowed for the regeneration of compound **2** by hydrosilylation of the benzaldehyde. NMR spectroscopy suggested an unusual mechanism for the transformation, involving a substrate insertion in the B−H bond of the borohydride anion.

## Introduction

The chemistry of frustrated Lewis pairs (FLPs) has received large attention in recent years, with a significant spark being the discovery of metal free hydrogen splitting in 2006 by Stephan and co‐workers.^1^, ^2^ The term FLP refers to Lewis acid–base pairs that contain unquenched acidic and basic centers.^3^ Usually, these Lewis acid–base pairs combine to a Lewis adduct by forming a chemical bond between the Lewis functionalities. The deliberate introduction of steric hindrance into bimolecular Lewis pairs or the introduction of spatially separated Lewis acid and base functionalities in one molecule (an ambiphilic compound), however, results in the formation of FLPs.^2^, ^3^, ^4^ The corresponding chemistry has evolved into much more than mere curiosity, as countless reports of small molecule activations and catalytic applications have been given, with the metal‐free hydrogenation of olefins being just one prominent example.^1^, ^3^, ^4^, ^5^, ^6^, ^7^, ^8^, ^9^, ^10^ The present understanding of FLP chemistry was notably influenced by findings made several years earlier in the investigation of borane‐mediated hydrosilylation reactions by Piers and co‐workers.^11^, ^12^

In recent years, the combination of unquenched Lewis pair reactivity and transition‐metal chemistry has received considerable attention, by developing transition‐metal‐based FLPs.^6^, ^13^, ^14^, ^15^, ^16^ The benefits of using transition metals include increased reactivity in small molecule activation, reactivity not observed with main group frustrated Lewis pairs and the advantage of an easy variation of the FLP components, for example, by ligand modifications.^6^, ^13^, ^17^

High valent metal oxido fragments usually react as electrophiles, as for example observed in oxygen‐atom transfer reactions to Lewis basic phosphanes.^18^, ^19^, ^20^, ^21^, ^22^, ^23^, ^24^ Nevertheless, there have been reports of metal oxides exhibiting ambiphilic reactivity.^25^, ^26^ The inherent nucleophilicity of M=O fragments has been showcased by Green and co‐workers, who reacted various transition metal oxides with B(C_6_F_5_)_3_ to obtain the corresponding Lewis adducts. However, they restricted their investigations to the thorough characterization of the compounds, while reactivity studies were not performed.^27^, ^28^, ^29^ On the other hand, examples of transition‐metal FLPs involving the versatile metal oxido functionality are surprisingly scarce. Very recently, Ison and co‐workers disclosed the capability of rhenium(V) oxido based Lewis adducts to catalytically hydrogenate alkenes, an unprecedented behavior for high valent metal oxido compounds.^30^, ^31^

**Scheme 1:**
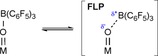
Lewis adduct/FLP equilibrium with a metal oxido fragment acting as Lewis base.

The postulated mechanism is based on the formation of a FLP with the metal oxido group as the Lewis basic component (Scheme 1), corroborated by mechanistic studies with H_2_/D_2_ mixtures that revealed isotopic scrambling (formation of HD) under catalytic conditions.^30^

FLPs in equilibrium with the corresponding classical Lewis adducts can be seen as quasi‐frustrated Lewis pairs, underlining that stable Lewis acid‐base adducts can exhibit FLP‐like reactivity under certain conditions.^1^, ^7^, ^9^, ^16^, ^30^, ^32^

For several years we have used molybdenum as an earth abundant and non‐toxic transition metal^33^ and develop high oxidation state molybdenum compounds, primarily for oxygen activation, oxygen‐atom‐transfer and oxidation reactions.^18^, ^19^, ^20^, ^23^, ^34^, ^35^, ^36^ With this in mind, we are exploring new concepts for the activation of metal peroxido and metal oxido moieties, with the latter being important intermediates in catalytic aerobic oxidation reactions, to increase the reactivity of this notoriously inert groups.^23^, ^36^

A promising approach seems to us the addition of a strong Lewis acid for the intermolecular activation. For a metal oxido moiety, the coordination of a Lewis acid should lead to a weakening of the M=O bond thus enabling novel reactivity, additionally aided by the prospect of FLP formation. The feasibility of the concept has very recently been demonstrated in dinitrogen complexes in which a Lewis acid led to the activation of the inert N_2_ molecule.^37^ Further examples of reactivity enhancement upon coordination of B(C_6_F_5_)_3_ at metal oxido moieties have been reported by the groups of Schrock^38^ and Ison.^39^

Catalytic hydrosilylation of organic carbonyl functions using B(C_6_F_5_)_3_ has been intensively investigated.^12^, ^40^, ^41^, ^42^ In fact, the general mechanism of B(C_6_F_5_)_3_‐catalyzed hydrosilylation, elucidated by the work of Piers and Oestreich, is based on the formation of a FLP with the substrate.^11^, ^12^, ^40^, ^41^, ^42^, ^43^, ^44^ In addition, some transition metal oxido complexes are known to be active catalysts in hydrosilylation reactions.^45^ While a vast number of transition metal compounds are known to react with hydrosilanes,^46^ only few examples of well‐characterized species resulting from the reaction of hydrosilanes with metal oxido groups have been disclosed by Abu‐Omar,^47^ Hayton^48^, ^49^ and Toste.^50^ For this reason, we found hydrosilanes a suitable choice as test reagents to assess the envisioned Lewis acid activation of the molybdenum(VI) oxido group.

Herein we report the cleavage of Si−H bonds at an oxidomolybdenum/borane Lewis pair, allowing for the isolation of novel cationic molybdenum(VI) silanolate species, showcasing the anticipated Lewis acid assisted increase of reactivity. The molybdenum(VI) oxido imido complex with B(C_6_F_5_)_3_, a classical Lewis acid–base adduct with the Lewis acid exclusively bound to the oxido ligand, is fully characterized. However, reactivity studies showed the B−O bonding to be reversible, corroborating the formation of a reactive Lewis pair in solution, capable to heterolytically cleave Si−H bonds. Subsequent reactivity studies towards benzaldehyde uncovered an unusual substrate insertion step into the borohydride bond under reformation of the Lewis adduct. The research presented here likewise offers the prospect of the templated transfer of not only silyl groups but also other electrophiles to a variety of substrates.

## Results and Discussion

### Lewis adduct synthesis

**Scheme 2:**
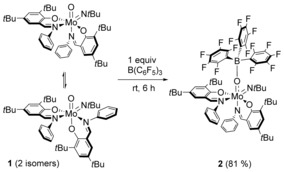
Synthesis of the Lewis adduct **2**.

Addition of one equivalent of the Lewis acid B(C_6_F_5_)_3_ to the yellow solution of the molybdenum(VI) oxido imido precursor [MoO(N*t*Bu)**L**_2_] (**1**)^35^ in pentane led to an immediate color change to deep‐red and subsequent formation of the Lewis adduct [Mo{OB(C_6_F_5_)_3_}(N*t*Bu)**L**_2_] (**2**) as a reddish precipitate. Compound **2** was isolated as a brick‐red solid in very good yield after purification (Scheme 2).

The slightly moisture‐sensitive complex **2** is soluble in most polar organic solvents, sparingly soluble in benzene and toluene and practically insoluble in acetonitrile and DMSO.

Interestingly, NMR spectroscopy revealed compound **2** to exist as a single isomer in solution, which contrasts with the isomeric equilibrium observed for **1**. We attribute this not only to the increased steric demand in the coordination sphere, but also to the pronounced electron‐withdrawing effect of the oxidoborane unit, leading to increased σ‐donation of the other ligands and thus decreased flexibility at the metal center. The single isomer further indicates that the Lewis acid exclusively coordinates at one terminal ligand, the Mo=O moiety, as shown by X‐ray crystallography (vide infra). The coordination of the Lewis acid is confirmed by a new set of signals corresponding to the *meta*, *ortho* and *para* fluorines of B(C_6_F_5_)_3_, respectively, observable by ^19^F NMR spectroscopy. The pronounced shift of the *para* F resonance (−161.9 ppm) compared to free borane (−142.0 ppm) is in agreement with literature.^25^ Compound **2** was further characterized via ^11^B NMR spectroscopy, which features a broad singlet resonance at 2.5 ppm, indicative of adduct formation.

To assess the stability of the B−O bond, that is, possible dissociation to form a FLP, the reactivity of **2** towards donor solvents known to form adducts with free B(C_6_F_5_)_3_, such as THF or MeCN, was examined. Whereas addition of THF did not lead to a reaction, addition of MeCN to a C_6_D_6_ solution of **2** led to an equilibrium between **2** and B(C_6_F_5_)_3_⋅MeCN. The ratio was found to be dependent on the added amount of MeCN (Figure S1, Supporting Information), demonstrating a rather stable B−O bond but nevertheless reversibility of the adduct formation.

A similar dynamic behavior has also been observed in Ison's rhenium based Lewis adducts, corroborated by variable temperature NMR spectroscopy.^30^, ^31^ Thus, variable temperature ^19^F NMR measurements of **2**/B(C_6_F_5_)_3_ (1:1 mixture) were performed, however, no coalescence between the signals of the adduct **2** and free B(C_6_F_5_)_3_ was observed in the investigated temperature range (up to 60 °C in [D_8_]toluene).

### Reactivity of 2 towards hydrosilanes

**Scheme 3:**
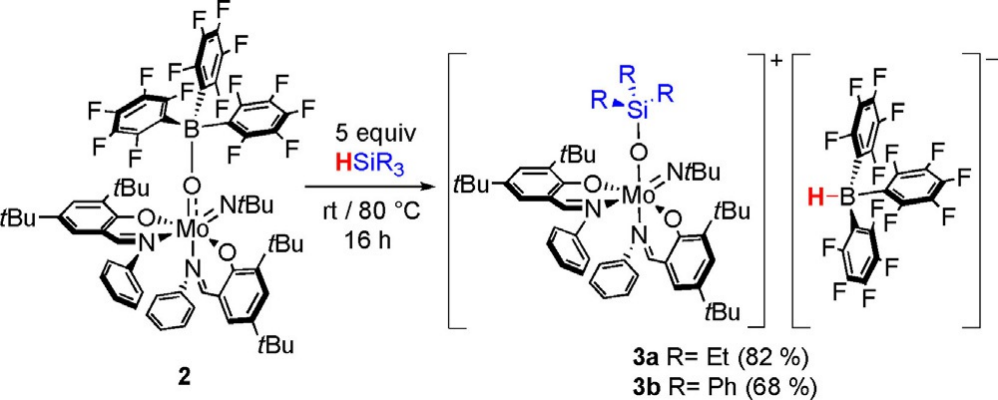
Formation of the cationic silanolate complexes **3 a** and **3 b**.

Reaction of **2** with 5 equivalents of Et_3_SiH at room temperature led to heterolytic cleavage of the Si−H bond and formation of the cationic molybdenum(VI) silanolate complex [Mo(OSiEt_3_)(N*t*Bu)**L**_2_][HB(C_6_F_5_)_3_] (**3 a**) with a hydridoborate anion as shown in Scheme 3. Similar reactivity was observed with Ph_3_SiH, leading to the corresponding ion pair [Mo(OSiPh_3_)(N*t*Bu)**L**_2_][HB(C_6_F_5_)_3_] (**3 b**). The steric demand as well as presumably the less polarized Si−H bond, however, required more drastic reaction conditions (80 °C, 16 h). Oxido imido complex **1** on the other hand did not react with Et_3_SiH or Ph_3_SiH after a reaction time of 24 h at 80 °C.

Complexes **3 a** and **3 b** were obtained in good yields after purification. They are very soluble in most polar and non‐polar organic solvents and only sparingly soluble in alkanes. The formation of the diamagnetic cationic Mo^VI^ species of the general structure [Mo(OSiR_3_)(N*t*Bu)**L**_2_]^+^ was confirmed by distinct signal sets for two iminophenolate, one imido, one silanolate ligand and a broad (1:1:1:1) quartet resonance for the [HB(C_6_F_5_)_3_]^−^ anion in the ^1^H NMR spectra. The structure of the anion is further confirmed by three new resonances in the ^19^F NMR spectra, and a characteristic doublet resonance in the ^11^B NMR spectra, both matching literature.^8^ Similar to **2**, **3 a** and **3 b** are present as a single isomer in solution, following the same reasoning presented above.

It is interesting to note that in C_6_D_6_, a non‐polar solvent, ^1^H NMR shifts of the Mo^VI^ cation are highly dependent on the concentration, while the ^19^F NMR signals are only slightly affected.

**Figure 1:**
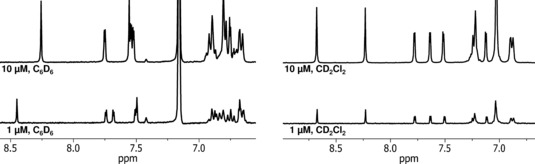
Concentration dependent ^1^H NMR resonances of **3 a** in C_6_D_6_ (left) and independent resonances in CD_2_Cl_2_ (right).

In contrast, in a polar solvent (CD_2_Cl_2_), ^1^H NMR resonances are not shifted upon change of concentration. This can be attributed to the increased solvation capability of the polar solvent and thus decreased ionic interaction (Figure 1).

The stability of complexes **3 a** and **3 b** in solution is limited as they are very sensitive towards moisture. Thus, ^1^H NMR spectra of **3 a** or **3 b**, respectively, in the presence of H_2_O, show a new set of ^1^H NMR signals, which can be attributed to **HL**H_2_,^51^ the protonated ligand with a reduced C=N moiety, underscoring the reactivity of the hydridoborate anion.^14^, ^52^, ^53^, ^54^ Infrared spectroscopy of **3 a** and **3 b** revealed no assignable Mo=O stretch. However, a broad peak in the Mo−O region at 567 cm^−1^ (**3 a**) and 568 cm^−1^ (**3 b**) indicates overlap of the stretches of three Mo−O bonds in total (Figure S3).

In complexes **1** and **2** on the other hand, a single sharp band was found at 544 and 546 cm^−1^, respectively, assigned to the two phenolate Mo−O stretches. The assignment of an additional Mo−O frequency is in good agreement with the bond lengths obtained via single‐crystal X‐ray diffraction analysis (vide infra). The infrared spectra of **3 a** and **3 b** also feature a characteristic broad B−H band at approximately 2380 cm^−1^, consistent with the hydridoborate anion (Figure S2).

Formation of complexes [Mo(OSiR_3_)(N*t*Bu)**L**_2_][HB(C_6_F_5_)_3_] (**3 a** and **3 b**) upon reaction of **2** with R_3_SiH is reminiscent of the ionic intermediate in the FLP‐based hydrosilylation mechanism formulated by Piers and Oestreich.^12^, ^43^ A major difference is the possibility of charge compensation by the metal in our case. The cationic molybdenum center is likely energetically favored over a hypothetic oxonium‐based ion. A B(C_6_F_5_)_3_ assisted reaction of a metal oxide with a hydrosilane has been disclosed previously for a high valent uranium system, however the reaction was accompanied by the reduction of the metal center and led to different products with Et_3_SiH and Ph_3_SiH.^48^, ^49^

**Scheme 4:**
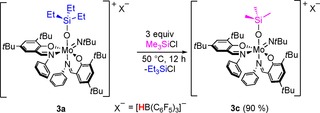
Silyl group exchange upon reaction of **3 a** with trimethylsilyl chloride.

In order to evaluate the strength of the formed Mo‐OSiR_3_ bonds, σ‐bond metathesis with Me_3_SiCl was investigated.^55^ Upon reaction of **3 a** with three equivalents of Me_3_SiCl, silyl group exchange was observed to form the complex [Mo(OSiMe_3_)(N*t*Bu)**L**_2_][HB(C_6_F_5_)_3_] (**3 c**) rather than metathesis to form (Me_3_Si)_2_O and a molybdenum chlorido species (Scheme 4). This is in agreement with the higher average bond dissociation energy of Mo−O (560 kJ mol^−1^)^56^ in comparison to Si−O (452 kJ mol^−1^).^57^ The observed reactivity represents a practical way to synthesize the trimethylsilanolate complex **3 c** in high yield without the need for the volatile Me_3_SiH. Nevertheless, (Me_3_Si)_2_O was frequently encountered as a by‐product in the reaction, a fact that is attributed to the pronounced sensitivity towards water.

### Anion exchange

As above mentioned, complexes **3 a**–**3 c** are very sensitive towards moisture, which is predominantly a result of the inherent reactivity of the hydridoborate anion, as previously discussed in literature.^14^, ^52^, ^53^, ^54^ Furthermore, **3 a**–**3 c** were obtained as amorphous solids from which X‐ray quality crystals could not be grown.

To stabilize the complexes and possibly enable crystallization, the exchange of the anion was investigated.

**Scheme 5:**
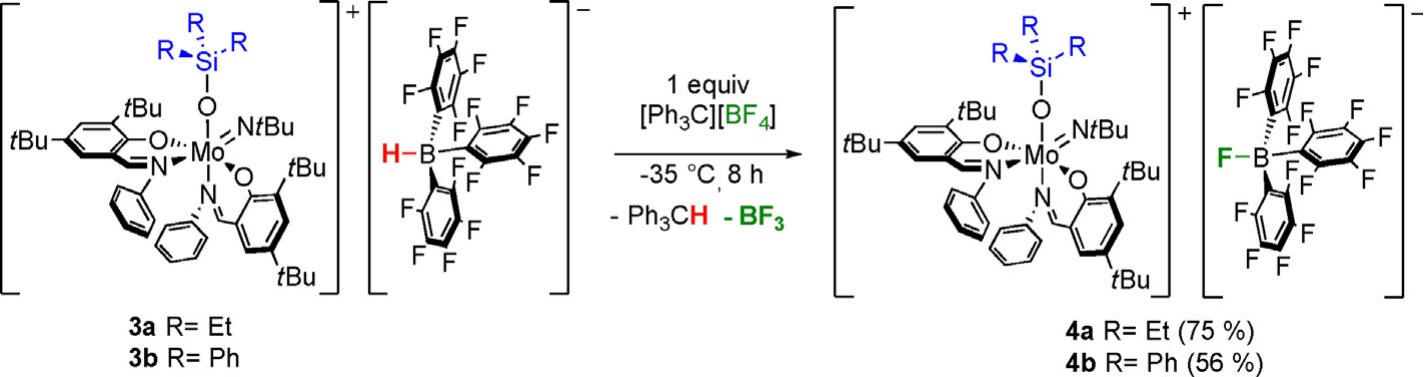
Reactions of **3 a** and **3 b** with tritylium tetrafluoroborate yielding compounds **4 a** and **4 b**.

The use of [Ph_3_C][BF_4_] should abstract the hydride from the hydridoborate and introduce the smaller and more stable anion [BF_4_]^−^. Thus, reaction of complexes **3 a** and **3 b**, respectively, with [Ph_3_C][BF_4_] at low temperatures afforded after work‐up two new species **4 a** and **4 b** in good yields as purple solids. Proton NMR spectroscopy revealed resonances that are virtually identical to those of **3 a** and **3 b**, respectively, albeit the B−H resonance is absent, consistent with an anion exchange. The absence of a B−H stretch is also confirmed via infrared spectroscopy. However, careful examination of ^19^F NMR data revealed an unexpected fluorination of the anion after hydride abstraction, leading to the formation of complexes [Mo(OSiR_3_)(N*t*Bu)**L**_2_][FB(C_6_F_5_)_3_] (**4 a**, R=Et and **4 b**, R=Ph) (Scheme 5), indicated by an additional broad resonance for the B−F moiety. The ^11^B NMR resonance observed for **4 b** (br d, −0.5 ppm) is in good agreement with literature, supporting the structure of the anion.^58^

The obtained complexes exhibit increased solubility in alkanes, compared to **3 a** and **3 b**. Thus, the anion modification allowed for the growth of single crystals suitable for X‐ray diffraction analysis for **4 b** from saturated pentane solutions. The stability of compounds **4 a** and **4 b**, especially towards moisture is, however, still low.

We attribute the fluoride transfer to the significantly higher Lewis acidity of B(C_6_F_5_)_3_ in comparison to BF_3_, which is in good agreement with the fluoride ion affinities (FIA) of the two species.^59^ A comparable behavior has been observed by Parkin and co‐workers for a Zn−F complex, in which the fluoride was largely transferred upon exposure to B(C_6_F_5_)_3_.^60^

We found that treatment of Na[HB(C_6_F_5_)_3_]^61^ or [NBu_4_][HB(C_6_F_5_)_3_]^61^ with [Ph_3_C][BF_4_] also lead to the formation of triphenylmethane and ^19^F NMR spectroscopy revealed new sets of signals (−140.2, −157.8, −163.6, −199.1 ppm for the Na^+^ salt and −136.3, −162.1, −166.7, −191.1 ppm for the [NBu_4_]^+^ compound) suggesting a similar fluoride transfer. These data are different to those found for **4 a** and **4 b**, but comparable to those of [NEt_4_][FB(C_6_F_5_)_3_],^62^ indicating that the fluoride transfer occurs independently of the used cation.

### Hydrosilylation reactivity of 3 a and 3 b

**Scheme 6:**
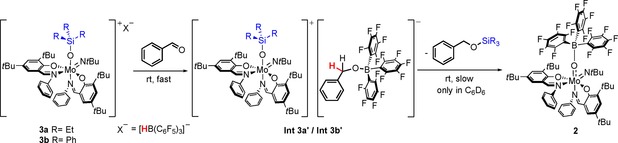
Observed reaction sequence after addition of benzaldehyde to **3 a** and **3 b**.

To evaluate the suitability of the formed ion pairs for hydrosilylation, the reaction of **3 a** with 2 equivalents of benzaldehyde as model substrate was investigated. Reaction progress was monitored by ^1^H and ^19^F NMR spectroscopy in C_6_D_6_ and CD_2_Cl_2_, respectively, revealing a two‐step process via an intermediate species **Int 3 a’** and finally the formation of benzyl silyl ether under regeneration of the Lewis adduct **2** (Scheme 6).

In apolar benzene, the ^19^F NMR spectrum recorded after 12 h revealed, next to approximately 35 % of regenerated **2** and 5 % of residual [HB(C_6_F_5_)_3_]^−^, resonances for a new B−C_6_F_5_ species (approx. 60 %) which we assign to the benzaldehyde inserted anion [PhCH_2_OB(C_6_F_5_)_3_]^−^ of **Int 3 a’** (Scheme 6 and Figure S4). This is consistent with the ^1^H NMR spectrum which reveals approximately 30 % of the hydrosilylation product BnOSiOEt_3_ and 60 % of **Int 3 a’**, showing a distinct resonance for the benzylic protons of [PhCH_2_OB(C_6_F_5_)_3_]^−^ at 4.8 ppm. It further reveals unchanged signals for the complex cation as well as disappearance of the B−H resonance (Figure S5). Reaction control after 72 h revealed full conversion to **2** and BnOSiEt_3_, also indicated by a color change from purple to red (Figures S4 and S5).

In the polar solvent CD_2_Cl_2_ again conversion to **Int 3 a’** occurs, albeit significantly faster. After 6 h, 75 % of **Int 3 a’** is formed and after 24 h full conversion is observed. Both ^19^F and ^1^H NMR spectroscopy indicate the formation of **Int 3 a’** due to unchanged resonances for the complex cation and no observable B−H resonance (Figures S6 and S7). Also in this solvent the ^1^H NMR resonance at 4.3 ppm is distinctive for the CH_2_ group in [PhCH_2_OB(C_6_F_5_)_3_]^−^, in good agreement with literature.^63^

Additionally, ^11^B NMR spectroscopy of **Int 3 a’** in CD_2_Cl_2_ revealed the disappearance of the doublet corresponding to the borohydride moiety of **3 a** (−25.4 ppm) and a new sharp singlet at −2.7 ppm, matching literature data for the [PhCH_2_OB(C_6_F_5_)_3_]^−^ anion (Figure S8).^62^

The nature of **Int 3 a’** was further elucidated by in situ addition of Et_3_SiD to a solution of **2** in CD_2_Cl_2_, resulting in the formation of **3 a**‐***d***_**1**_ (Figure S17), and subsequent addition of benzaldehyde. The resulting ^1^H NMR spectrum revealed the ‐(CHD)‐ methylene resonance integrating for only one proton, compared to two in the non‐deuterated compound (Figure S9). Additionally, the corresponding ^13^C NMR spectrum showed resonances for the aromatic benzylate protons in agreement with literature as well as a triplet for the ‐(CHD)‐ moiety (^1^ *J*_(C−D)_≈21 Hz), confirming insertion into the B−D bond (Figure S10).^62^

It is interesting to note, that in CD_2_Cl_2_ further reaction of **Int 3 a’** forming BnOSiEt_3_ virtually does not occur (yield of **2** and BnOSiEt_3_ after 72 h ≈5 %). Possibly, the polar solvent leads to a larger separation of the ion pair and thus inhibits reaction of the benzyloxy borate with the silyl group (Scheme 6).

The reactivity of compound **3 b** is similar to that of **3 a**, albeit with considerably lower chemoselectivity. Nevertheless, ^19^F NMR data revealed resonances for **Int 3 b’** virtually identical to those of **Int 3 a’**. Also the ^1^H NMR spectrum in CD_2_Cl_2_ shows the distinct resonance at 4.3 ppm for the CH_2_ group of the benzaldehyde inserted anion.

We were interested whether benzaldehyde insertion into the B−H bond also occurs in other borate salts such as Na[HB(C_6_F_5_)_3_]^61^ or [NBu_4_][HB(C_6_F_5_)_3_]^61^ but found no reactivity. This suggests that the [Mo(OSiR_3_)(N*t*Bu)**L**_2_]^+^ cation is required, possibly by activating the substrate due to its Lewis acidic properties. Similarly, a recent report on the insertion of alkynes into the B−H bond of [NBu_4_][HB(C_6_F_5_)_3_] describes the need for catalytic amounts of the strong Lewis acid B(C_6_F_5_)_3_.^64^

Based on the data described above the following steps are suggested for the reaction shown in Scheme 6:

i) the Lewis acidic molybdenum cation activates the substrate which allows, under nucleophilic attack of the hydride, insertion into the B−H bond;

ii) in the apolar solvent, the ion pair is in closer contact so that the electrophilic silicon (activated by the electron poor molybdenum center) reacts with the benzyloxy borate, leading to the formation of the hydrosilylated product BnOSiEt_3_ and the Lewis adduct **2**. The presumably low nucleophilicity of [PhCH_2_OB(C_6_F_5_)_3_]^−^ leads to the observed slow product formation.

Although a mechanism based on direct attack of the benzaldehyde at the silyl group and subsequent hydride transfer could be envisioned, our spectroscopic evidence rules against it.

The presented findings thus suggest a mechanism different to traditional FLP‐based reaction pathways, such as for example reported for rhenium oxido/borane Lewis pair catalyzed alkene hydrogenation^31^ or catalytic hydrosilylation using a nickel or cobalt/borane Lewis pair.^64^ Whereas mechanisms involving such an initial formal hydroboration of the substrate are scarce, they have been described previously in FLP‐based CO_2_ hydrogenation and alkyne hydroboration reactions, the latter also requiring substrate activation by a Lewis acid.^10^, ^65^ A comparable mechanism has very recently been proposed for a Mg/Zn mediated CO_2_ hydrosilylation reported by Parkin and co‐workers.^66^

### UV/Vis spectroscopy

**Figure 2:**
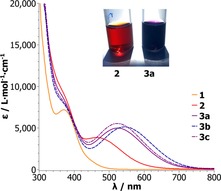
UV/Vis absorption spectra of **1**,^35^ **2** and **3 a**–**3 c** in CH_2_Cl_2_ and photograph of solutions of **2** and **3 a** (CH_2_Cl_2_).

A noteworthy property of the Lewis adduct [Mo{OB(C_6_F_5_)_3_}(N*t*Bu)**L**_2_] (**2**) as well as the ionic compounds [Mo(OSiR_3_)(N*t*Bu)**L**_2_][HB(C_6_F_5_)_3_] (**3 a**–**3 c**) is their intense color, reflected by the respective UV/Vis absorption spectra (Figure 2). The spectral properties of **2** and **3 a**–**3 c** are unusual for molybdenum(VI) complexes and indicative of charge‐transfer phenomena, which is corroborated by the order of magnitude of the corresponding molar extinction coefficients (Table 1).

**Table 1:** UV/Vis parameters of complexes **1**,^35^ **2** and **3 a**–**3 c** in CH_2_Cl_2_.

	*λ*_max,vis_ [nm]	*ϵ* [L⋅mol^−1^⋅cm^−1^]	*E* [eV]
**1**	451 (sh)^[a]^	680	2.75
**2**	467	3750	2.65
**3 a**	526	4850	2.36
**3 b**	547	5020	2.27
**3 c**	526	5310	2.36

[a] Shoulder.

While a ligand to ligand charge transfer cannot be ruled out, the electropositive metal center favors a ligand to metal (LMCT) transition, originating either from a siloxido or imido based lone pair. The observed transition energies reflect the electronic situation at the molybdenum metal center. Whereas the corresponding transition found for complex [MoO(N*t*Bu)**L**_2_] (**1**) is comparatively weak and of high energy, it is redshifted in complex **2**, corresponding to lower transition energy. Those for complexes **3 a**–**3 c** are shifted even more to lower energy/higher wavelength, with the more electron‐withdrawing triphenyl siloxide ligand in **3 b** resulting in the lowest transition energy. The trend correlates with energetically low, more accessible unoccupied molecular orbitals in the compounds with a highly electropositive metal center and thus substantiates the pronounced effect of Lewis acid coordination, as well as ionization, on the metal center.

### Molecular structures

**Figure 3:**
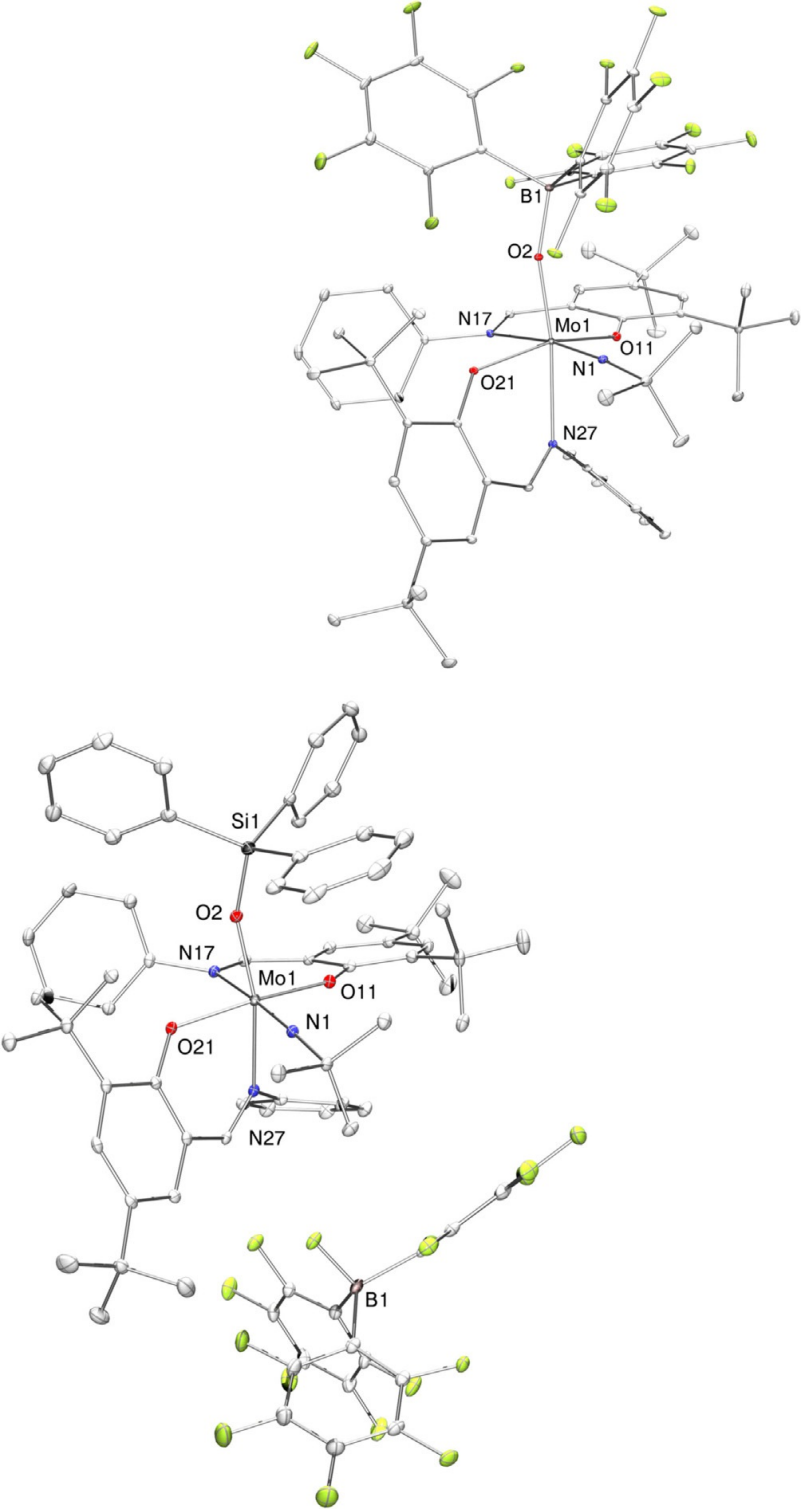
Molecular views (50 % probability level) of the Lewis adduct complex [Mo{OB(C_6_F_5_)_3_}(N*t*Bu)**L**_2_] (**2**, top) and the cationic imido silanolate complex [Mo(OSiPh_3_)(N*t*Bu)**L**_2_][FB(C_6_F_5_)_3_] (**4 b**, bottom). H atoms as well as solvent molecules are omitted for clarity reasons.

The molecular structures of **2** and **4 b** were determined by single‐crystal X‐ray diffraction analysis. Selected bond lengths are given in Table 2, molecular views of **2** and **4 b** are given in Figure 3. Full crystallographic details such as structure refinement and experimental details are provided within the Supporting Information.

**Table 2:** Comparison of selected bond lengths (Å) for complexes **1**,^35^ **2** and **4 b**.

	**1** 35	**2**	**4 b**
Mo=O^[a]^	1.7198(18)	1.8221(9)	1.8975(19)
Mo=N*t*Bu	1.739(2)	1.7259(12)	1.715(2)
N−*t*Bu	1.448(3)	1.4581(17)	1.470(4)
Mo−O_phenolate_	1.9680(18)	1.9583(10)	1.9391(18)
	1.9953(18)	1.9570(9)	1.9365(18)
Mo−N_imine_	2.390(2)	2.3140(12)	2.315(2)
	2.366(2)	2.2957(11)	2.214(2)

[a] Mo−OSiR_3_ in **4 b**.

The Mo=O bond length in **2** is substantially elongated in comparison to the parent complex **1**, 1.8221(9) versus 1.7198(18) Å.^35^ Previously reported molybdenum(VI) oxido borane adducts showed a similar bond elongation.^28^, ^29^, ^67^ The bonds from all other donor atoms to the metal center are slightly shortened, causing a decreased flexibility around the metal center, thereby most likely preventing isomerization (vide supra). The Mo−OSi bond length in **4 b**, 1.8975(19) Å, is comparable to Mo^VI^ complexes bearing triphenylsilanolate ancillary ligands, corroborating a reduction of the molybdenum oxido bond.^68^, ^69^ Also, the clearly bent Si‐O‐Mo angle of 155.54(12)° causes a limitation in orbital overlap between oxygen and molybdenum, in good agreement with a single bond to an electrophilic metal center.^69^ All other ligands are bound tighter to the metal center to compensate for the strongly increased electropositive nature, which is reflected by a mean shortening of the Mo−O, Mo−N and Mo=N_imide_ bond lengths of approximately 0.02–0.15 Å per bond, going from **1** to **4 b**. Overall, the ensemble of bond lengths in the first coordination sphere well reflects the decreasing electron density at the molybdenum center in the series **1**–**2**–**4 b**.

### Electrochemistry

To get a deeper insight into the electronic situation at the metal center, the electrochemical behavior of complexes **1**, **2** and **3 a**–**3 c** was investigated by cyclovoltammetry. Cyclovoltammograms of **1** and **3 a**–**3 c** were recorded in acetonitrile, while for complex **2**, CH_2_Cl_2_ was used for solubility reasons. Data was referenced to the ferrocene Fc/Fc^+^ redox couple in MeCN and CH_2_Cl_2_, respectively, using the same conditions.

**Figure 4:**
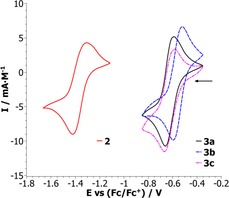
Cyclovoltammograms of **2** and **3 a**–**3 c** (scan rate 100 mV s^−1^), depicting the assigned Mo^VI^/Mo^V^ redox couples.

Whereas complex **1** exhibits no redox process in the observed potential range, the CV of complex **2** depicts a redox event at −1.37 V versus Fc/Fc^+^. For the cationic Mo^VI^ imido silanolate complexes, the Mo^VI^/Mo^V^ redox couples experience a large anodic shift (−0.62 V, −0.55 V and −0.63 V vs. Fc/Fc^+^ for **3 a**–**3 c**, respectively) in comparison to the peak potential found for complex **2**, pointing towards a much more electropositive Mo^VI^ metal center (Figure 4).

Given the assumption that the Mo^VI^/Mo^V^ couple for complex **1** lies outside of the experimentally accessible voltage range, this clearly corroborates that increased electrophilicity of the molybdenum metal center facilitates one‐electron reduction of Mo^VI^ to Mo^V^.

In general, the potential found for **2** is comparable to redox couples previously reported for dioxido molybdenum(VI) compounds, with oxido imido compounds usually exhibiting significantly higher potentials due to a potent electron donating capability of the imido ligand.^22^, ^24^, ^70^ However, the anodic shift of more than 0.7 V for the redox couples of **3 a**–**3 c**, in comparison to **2**, leads to remarkably low reduction potentials (Figure 4).

To evaluate the reversibility of the redox processes found for complexes **2** and **3 a**–**3 c** (Table 3), the scan rate (ν) dependence of **2** and **3 a** was investigated.

**Table 3:** Electrochemical parameters for the Mo^VI^/Mo^V^ redox couples of **2** and **3 a**–**3 c** in MeCN.

	*E*_p,ox_ [V]	*E*_p,red_ [V]	*E*_1/2_ [V]	Δ*E*_p_ [mV]	*I*_pa_/*I*_pc_
**2** ^[a]^	−1.32	−1.41	−1.37	91.0	0.86
**3 a**	−0.60	−0.65	−0.62	57.6	0.93
**3 b**	−0.52	−0.59	−0.55	74.2	0.99
**3 c**	−0.58	−0.67	−0.63	85.1	0.88

[a] Solvent: CH_2_Cl_2_.

For compound **3 a**, the peak separation in the cyclic voltammogram is scan rate independent and the peak current linear dependent on ν^1/2^, corroborating a reversible process. In contrast, assessment of the redox couple found for the oxido/borane adduct **2** reveals an increase in peak separation at higher scan rates pointing towards electrochemical irreversibility (Figure S3).

Such irreversibility is often caused by chemical instability of the analyte, which can likely be attributed to the dynamics of the system in solution (lability of the B−O bond) although solvent specific phenomena as well as slow electron transfer rates cannot be excluded a priori. Due to the low value for *I*_pa_/*I*_pc_ for compound **3 c**, the same scan rate study was also performed, showing scan rate independence thereby suggesting reversibility (Figure S3).

These findings indicate that the redox couples for **3 a**–**3 c** are truly reversible and likely metal based. This is highly interesting, given that the reduction of molybdenum(VI) to molybdenum(V) is frequently hampered by irreversible dimerization. An additional benefit of the system is the possibility for an electronic fine tuning by simply varying the silanolate group.

## Conclusions

The reported molybdenum oxido based Lewis adduct [Mo{OB(C_6_F_5_)_3_}(N*t*Bu)**L**_2_] featuring reversible B−O bonding reacts with tertiary silanes to form highly unusual ion pairs of the type [Mo(OSiR_3_)(N*t*Bu)**L**_2_][HB(C_6_F_5_)_3_] (R=Et, Ph). This is not only a rare example of FLP‐like reactivity involving the widespread transition metal oxido functionality but also gives access to high valent molybdenum species with unique spectroscopic and electronic properties of potential interest to a broad field of chemistry. Furthermore, it represents a rare instance of a Lewis acid mediated activation of the oxido ligand. The described ion pairs are further reactive towards benzaldehyde, regenerating the initial Lewis adduct via formation of the respective benzyloxy silane in a stepwise manner. This reactivity is highly dependent on the polarity of the employed solvent, with polar solvents inhibiting the formation of the hydrosilylation product. This supports mechanistic considerations suggesting an unusual two step pathway involving insertion of the benzaldehyde into the borohydride bond of the anion, forming the intermediate species [Mo(OSiR_3_)(N*t*Bu)**L**_2_][PhCH_2_O−B(C_6_F_5_)_3_], which is supported by spectroscopic investigations. In summary, the presented system combines the advantage of a Lewis adduct with the reactivity of a reversibly formed FLP. We believe the remarkable stability of the isolated silanolate borohydride ion pairs to be a result of electronic stabilization caused by the metal center ancillary to the Lewis basic oxido group. The research disclosed here is of particular interest because of the abundance of metal oxido motifs and the potential suitability to other M=O compounds. The here presented step‐wise reactivity also offers the prospect of a controlled, metal‐templated transfer of silyl groups and possibly other electrophiles.

## Experimental Section

### General

Unless specified otherwise, all experiments were performed under inert conditions using standard Schlenk equipment or a N_2_‐filled glovebox. Commercially available chemicals were used as received. Air and moisture sensitive chemicals were stored in Schlenk flasks or under N_2_ atmosphere in a glovebox; liquids were additionally stored over molecular sieves. The metal precursor [MoO(N*t*Bu)Cl_2_(dme)],^71^ the ligand **HL** 51 as well as B(C_6_F_5_)_3_ 72 were synthesized according to known procedures. Solvents were purified via a Pure‐Solv MD‐4‐EN solvent purification system from Innovative Technology, Inc. The ^1^H, ^11^B, ^13^C, ^19^F and HSQC NMR spectra were recorded on a Bruker Optics instrument at 300/96/75/282 MHz. Peaks are denoted as singlet (s) doublet (d), doublet of doublets (dd), triplet (t), quartet (q) and multiplet (m), broad peaks are denoted (br) and all peaks are referenced to the solvent residual signal. Shifts in ^11^B and ^19^F NMR are referenced to external standards (BF_3_⋅Et_2_O and CFCl_3_, respectively). Used solvents and peak assignment are mentioned at the specific data sets. HR‐MS (ESI^+^/ESI^−^) measurements were performed at the University of Graz, Department of Analytical Chemistry, using a Thermo Scientific Q‐Exactive mass spectrometer in positive and negative ion mode, the used solvent was acetonitrile. Peaks are denoted as ionic mass peaks, and the unit is the according ions mass/charge ratio. Calculated and found isotopic patterns are provided within the supporting information. Samples for infrared spectroscopy were measured on a Bruker Optics ALPHA FT‐IR Spectrometer. IR bands are reported with wavenumber (cm^−1^) and intensities (s, strong; m, medium; w, weak). Elemental analyses were measured at the Graz University of Technology, Institute of Inorganic Chemistry using a Heraeus Vario Elementar automatic analyzer. Deviations in the found elemental compositions of ionic compounds (low carbon content) are attributed to the pronounced water sensitivity as also observed by HR‐MS measurements. In all cases, addition of H_2_O to the elemental compositions would diminish the observed error.

### UV/Vis spectroscopy

UV/Vis spectra were recorded on a Varian Cary 50 spectrophotometer in a quartz cuvette with an optic path length of 1 mm. Analyte solutions were prepared in CH_2_Cl_2_ near 1 mm. Peak maxima are reported with wavelength (nm) and molar exctinction coefficient (M⋅cm^−1^), overlapping peak maxima are denoted as shoulders. All maxima and the corresponding absorptivities were obtained via deconvolution in the SciDaVis^73^ software using a scaled Levenberg–Marquardt algorithm.^74^

### X‐ray diffraction analyses

Single‐crystal X‐ray diffraction analyses were measured on a BRUKER‐AXS SMART APEX II diffractometer equipped with a CCD detector. All measurements were performed using monochromatized Mo_Kα_ radiation from an Incoatec microfocus sealed tube at 100 K (cf. Table S1). Absorption corrections were performed semi‐empirical from equivalents. Structures were solved by direct methods (SHELXS‐97)^75^ and refined by full‐matrix least‐squares techniques against *F* ^2^ (SHELXL‐2014/6).^75^ Full experimental details for single‐crystal X‐ray diffraction analyses of all compounds are provided in the Supporting Information.

### Electrochemistry

Electrochemical measurements were performed in a glovebox under N_2_ atmosphere in dry solvents with a Gamry Instruments Reference 600 Potentiostat using a three‐electrode setup. Used electrodes were glassy carbon as working electrode, Pt wire (99.99 %) as supporting electrode and an Ag wire immersed in a solution containing 10 mm AgNO_3_ and 100 mm [NBu_4_][PF_6_] in CH_3_CN, separated from the analyte solution by a Vycor tip, as a reference electrode. Analyte concentrations ranged from 0.2 to 1.0 mm in CH_3_CN or CH_2_Cl_2_, respectively. The supporting electrolyte used was [NBu_4_][PF_6_] (100 mm). Cyclic voltammetry data was smoothed in the SciDaVis^73^ software using a moving average filter. Full sweep‐width cyclic voltammograms are provided in the Supporting Information.

### Syntheses

**2,4‐Di‐*tert*‐butyl‐6‐((phenylimino)methyl)phenol (HL)**: Analytical data is in agreement with literature,^51^ additional ^1^H NMR data in CD_2_Cl_2_ is given for comparison reasons. ^1^H NMR (300 MHz, CD_2_Cl_2_, 25 °C): *δ*=13.71 (br s, 1 H, OH), 8.69 (s, 1 H, CH=N), 7.51–7.39 (m, 3 H, ArH), 7.36–7.24 (m, 4 H, ArH), 1.48 (s, 9 H, *t*Bu), 1.35 ppm (s, 9 H, *t*Bu).

**Tris(pentafluorophenyl)borane B(C_6_F_5_)_3_**: Analytical data is in agreement with literature,^72^ additional ^11^B and ^19^F NMR data in CD_2_Cl_2_ is given for comparison reasons. ^11^B NMR (CD_2_Cl_2_, 25 °C): *δ*=60.25 ppm (br s); ^19^F NMR (282 MHz, CD_2_Cl_2_, 25 °C): *δ*=−126.29 (br d, 6F, *o*‐F), −141.99 (br s, 3F, *p*‐F), −159.10 ppm (br m, 6F, *m*‐F).

**Complex syntheses**: All complexes except **2** are very sensitive towards moisture in solution and solid state, **2** is sensitive towards moisture in solution. They can be stored at ambient temperature in a N_2_‐filled glovebox for several weeks without decomposition.

**Improved synthesis of [MoO(N*t*Bu)L_2_] (1)**:^35^ For the synthesis of **1**, a solution of 1 equiv of [MoO(N*t*Bu)Cl_2_(dme)] (1.13 g, 3.28 mmol) in acetonitrile (20 mL) was added dropwise to a suspension of 2 equiv **HL** (2.03 g, 6.57 mmol) and 2.2 equiv NEt_3_ (1.01 mL, 7.22 mmol) in acetonitrile (20 mL) with stirring. The addition was accompanied by an immediate color change from bright yellow to deep‐red and the reaction mixture was stirred overnight. Subsequently the red mixture was evaporated in vacuo. Toluene (25 mL) was added to the red residue, resulting in a red solution and a reddish solid that was filtered off and washed with toluene (2×10 mL) until it was essentially colorless (NEt_3_⋅HCl). The red filtrate was evaporated in vacuo, followed by the addition of acetonitrile (10 mL). After 15 minutes of stirring, a bright yellow solid precipitated, which was isolated by filtration. The solid was re‐dissolved in *n*‐pentane (50 mL), filtered, and the resulting solution was evaporated in vacuo to obtain **1** as yellowish‐orange colored solid (2.11 g, 80 %). Analytical data is in agreement with literature,^35^ additional ^1^H NMR an UV/Vis data in CD_2_Cl_2_ is given for comparison reasons. ^1^H NMR (CD_2_Cl_2_, 25 °C, major isomer): *δ*=8.30 (s, 1 H, CH=N), 8.24 (s, 1 H, CH=N), 7.43 (dd, 2 H, ArH), 7.26–6.95 (m, 12 H, ArH), 1.30 (s, 18 H, *t*Bu), 1.19 (s, 9 H, *t*Bu), 1.08 (s, 9 H, *t*Bu), 1.06 ppm (s, 9 H, *t*Bu); ^1^H NMR (CD_2_Cl_2_, 25 °C, minor isomer): *δ*=8.00 (s, 1 H, CH=N), 7.87 (s, 1 H, CH=N), 7.56 (dd, 2 H, ArH), 7.26–6.95 (m, 7 H, ArH), 6.92–6.87 (m, 3 H, ArH), 6.81–6.75 (m, 2 H, ArH), 1.50 (s, 9 H, *t*Bu), 1.38 (s, 9 H, *t*Bu), 1.32 (s, 9 H, *t*Bu), 1.30 (s, 9 H, *t*Bu), 1.29 ppm (s, 9 H, *t*Bu); IR (ATR): $\tilde{\nu }$ =2952 (m, C−H), 1611 (s, C=N), 1434 (s), 1252 (m), 1186 (s), 890 (s, sh, Mo=O), 838 (s), 764 (s), 543 cm^−1^ (s, Mo‐O); UV/Vis [CH_2_Cl_2_; *λ*_max_ (*ϵ*)]: 372 nm (7340 m ^−1^ cm^−1^), 451 nm (sh, 680 m ^−1^ cm^−1^).

**Synthesis of [Mo{OB(C_6_F_5_)_3_}(N*t*Bu)L_2_] (2)**: For the synthesis of **2**, a solution of 1 equiv B(C_6_F_5_)_3_ (313 mg, 0.61 mmol) in dry pentane (5 mL) was added to a solution of 1 equiv of **1** (490 mg, 0.61 mmol) in the same solvent (10 mL). The addition was accompanied by an immediate color change from yellow to dark red. The reaction mixture was subsequently stirred at room temperature for 6 h, whereupon a large quantity of a red crystalline precipitate had formed. The precipitate was subsequently filtered off, washed with cold pentane (10 mL) and acetonitrile (5 mL) and dried in vacuo to yield **2** as a microcrystalline brick‐red solid (649 mg, 81 %). Single crystals suitable for X‐ray diffraction analysis were obtained via crystallization from a concentrated pentane solution of **2** at −35 °C. ^1^H NMR (CD_2_Cl_2_, 25 °C): *δ*=8.42 (s, 1 H, CH=N), 7.95 (s, 1 H, CH=N), 7.69 (d, 1 H, ArH), 7.42 (d, 1 H, ArH), 7.33 (d, 1 H, ArH), 7.24–7.11 (m, 3 H, ArH), 7.01–6.81 (m, 7 H, ArH), 6.79 (d, 1 H, ArH), 1.33 (s, 9 H, *t*Bu), 1.25 (s, 9 H, *t*Bu), 1.19 (s, 9 H, *t*Bu), 1.03 (s, 9 H, *t*Bu), 1.01 ppm (s, 9 H, *t*Bu); ^11^B NMR (CD_2_Cl_2_, 25 °C): *δ*=2.49 ppm (br s); ^13^C NMR (CD_2_Cl_2_, 25 °C, C−F obscured): *δ*=171.97, 169.49 (C=N), 163.60, 156.81 (ArO), 153.68, 150.88, 144.37, 144.00, 139.49, 137.77 (*q*‐C), 132.81, 132.31, 131.16 (ArH), 128.89 (Ph), 128.74 (ArH), 128.61, 127.45, 126.85, 124.96, 124.36 (Ph), 122.67, 121.95 (*q*‐C), 77.89 (imido‐*q*‐*t*Bu), 35.62 (2x), 34.82, 34.56 (*q*‐*t*Bu), 31.48, 31.39, 30.72, 30.53, 29.70 ppm (*t*Bu); ^19^F NMR (CD_2_Cl_2_, 25 °C): *δ*=−129.04 (dd, 6 F, *o*‐F), −161.87 (t, 3 F, *p*‐F), −166.33 ppm (m, 6 F, *m*‐F); IR (ATR): $\tilde{\nu }$ =2962 (m, C−H), 1606 (w, C=N), 1513 (m), 1461 (s), 1244 (m), 1085 (s), 979 (s), 896 (s), 832 (s), 765 (s), 546 cm^−1^ (s, Mo−O); UV/Vis [CH_2_Cl_2_; *λ*_max_ (*ϵ*)]: 369 nm (5980 m ^−1^ cm^−1^), 467 nm (sh, 3750 m ^−1^ cm^−1^). Anal. calcd for C_64_H_61_BF_15_MoN_3_O_3_: C, 58.59; H, 4.69; N, 3.20; Found: C, 58.44; H, 4.51; N, 3.52.

**Synthesis of [Mo(OSiEt_3_)(N*t*Bu)L_2_][HB(C_6_F_5_)_3_] (3 a)**: For the synthesis of **3 a**, 5 equiv of Et_3_SiH (61.2 μL, 0.38 mmol) were added to a solution of 1 equiv of **2** (100 mg, 0.08 mmol) in toluene (15 mL). After stirring for 16 h at room temperature, the color had changed to purple. After removal of all volatiles, the residual sticky substance was re‐dissolved in acetonitrile (6 mL) and filtered. The solvent was subsequently evaporated and the residual solid washed twice with pentane (2×5 mL) and thoroughly dried in vacuo to obtain **3 a** as a fluffy dark purple solid (104 mg, 82 %). ^1^H NMR (CD_2_Cl_2_, 25 °C): *δ*=8.67 (s, 1 H, CH=N), 8.23 (s, 1 H, CH=N), 7.78 (d, 1 H, ArH), 7.63 (d, 1 H, ArH), 7.51 (d, 1 H, ArH), 7.31–7.19 (m, 3 H, Ph), 7.12 (d, 1 H, ArH), 7.09–6.99 (m, 5 H, Ph), 6.92–6.84 (m, 2 H, Ph), 4.15–3.00 (1:1:1:1 br q, 1 H, B−H), 1.36 (s, 9 H, *t*Bu), 1.35 (s, 9 H, *t*Bu), 1.30 (s, 9 H, *t*Bu), 1.20 (s, 9 H, *t*Bu), 1.03 (s, 9 H, *t*Bu), 0.85 (t, 9 H, SiEt_3_), 0.66–0.55 ppm (m, 6 H, SiEt_3_); ^11^B NMR (CD_2_Cl_2_, 25 °C): *δ*=−25.44 ppm (d, ^1^ *J*_(B−H)_=89.9 Hz); ^13^C NMR (CD_2_Cl_2_, 25 °C, C−F obscured): *δ*=170.79, 170.65 (C=N), 162.32, 157.84 (ArO), 151.71, 150.43, 148.00, 146.32, 138.67, 137.74 (*q*‐C), 134.36, 132.95, 131.39 (ArH), 129.70, 129.48 (Ph), 129.39 (ArH), 128.51, 128.08, 124.23, 123.24 (Ph), 122.35, 121.69 (*q*‐C), 79.28 (imido‐*q*‐*t*Bu), 36.00, 35.57, 35.06, 35.05 (*q*‐*t*Bu), 31.39 (2x), 30.60, 30.15, 29.87 (*t*Bu), 7.29, 7.19 ppm (SiEt_3_); ^19^F NMR (CD_2_Cl_2_, 25 °C): *δ*=−134.02 (m, 6 F, *o*‐F), −164.88 (t, 3 F, *p*‐F), −167.71 ppm (m, 6 F, *m*‐F); IR (ATR): $\tilde{\nu }$ =2960 (m, C−H), 2386 (br w, B−H), 1591 (w, C=N), 1549 (w), 1507 (m), 1460 (s), 1243 (m), 1181 (w), 1096 (w), 966 (s), 886 (s), 841 (s), 764 (m), 704 (w), 567 cm^−1^ (s, Mo−O); UV/Vis [CH_2_Cl_2_; *λ*_max_ (*ϵ*)]: 360 nm (8320 m ^−1^ cm^−1^), 526 nm (sh, 4850 m ^−1^ cm^−1^). HR‐MS: (ESI^+^) *m*/*z* [*M*]^+^ calcd for C_52_H_76_MoN_3_O_3_Si: 916.4717, found: 916.4711; HR‐MS: (ESI^−^) *m*/*z* [HOB(C_6_F_5_)_3_]^−^ calcd for C_18_HBF_15_O: 528.9890, found: 528.9789; [HB(C_6_F_5_)_3_]^−^ calcd for C_18_HBF_15_: 512.9940, found: 512.9843; Anal. calcd for C_70_H_77_BF_15_MoN_3_O_3_Si⋅CH_3_CN: C, 58.86; H, 5.49; N, 3.82; Found: C, 59.01; H, 5.50; N, 4.03.

**Synthesis of [Mo(OSiPh_3_)(N*t*Bu)L_2_][HB(C_6_F_5_)_3_] (3 b)**: For the synthesis of **3 b**, 5 equiv of Ph_3_SiH (53 mg, 0.20 mmol) were added to a solution of 1 equiv of **2** (55 mg, 0.04 mmol) in toluene (5 mL). After stirring for 16 h at 80 °C, the color had changed to violet. After removal of all volatiles, the residual oily substance was washed three times with pentane (3×10 mL) and dried in vacuo to obtain **3 b** as a dark‐violet solid (45 mg, 68 %). ^1^H NMR (CD_2_Cl_2_, 25 °C): *δ*=8.68 (s, 1 H, CH=N), 8.15 (s, 1 H, CH=N), 7.80 (d, 1 H, ArH), 7.62 (d, 1 H, ArH), 7.53 (d, 1 H, ArH), 7.45–7.23 (m, 12 H, Ph), 7.17–7.08 (m, 6 H, Ph), 7.06 (d, 1 H, ArH), 7.03–6.92 (m, 5 H, Ph), 6.83–6.75 (m, 2 H, Ph), 4.04–3.11 (1:1:1:1 br q, 1 H, B‐H), 1.35 (s, 9 H, *t*Bu), 1.34 (s, 9 H, *t*Bu), 1.21 (s, 9 H, *t*Bu), 0.94 (s, 9 H, *t*Bu), 0.81 ppm (s, 9 H, *t*Bu); ^11^B NMR (CD_2_Cl_2_, 25 °C): *δ*=−25.42 ppm (d, ^1^J_(B_‐_H)_=91.0 Hz); ^13^C NMR (CD_2_Cl_2_, 25 °C, C−F obscured): *δ*=171.11, 170.33 (C=N), 163.02, 157.26 (ArO), 151.48, 148.93, 148.67, 146.78, 138.99, 138.09 (*q*‐C), 136.28 (*o*‐SiPh_3_), 134.62 (*q*‐SiPh_3_), 134.52, 133.12, 131.51 (ArH), 130.91 (*p*‐SiPh_3_), 129.81, 129.30 (Ph), 129.06 (ArH), 128.51, 128.33 (Ph), 128.29 (*m*‐SiPh_3_), 124.84, 123.99 (Ph), 122.66, 122.11 (*q*‐C), 79.65 (imido‐*q*‐*t*Bu), 35.97, 35.75, 35.17, 35.07 (*q*‐*t*Bu), 31.42, 31.35, 30.55, 30.01, 29.75 ppm (*t*Bu); ^19^F NMR (CD_2_Cl_2_, 25 °C): *δ*=−133.95 (m, 6F, *o*‐F), −164.82 (m, 3 F, *p*‐F), −167.64 ppm (m, 6 F, *m*‐F); IR (ATR): $\tilde{\nu }$ =2960 (m, C−H), 2387 (br w, B‐H), 1590 (m, C=N), 1548 (w), 1508 (m), 1459 (s), 1243 (m), 1180 (w), 1088 (s), 966 (s), 889 (s), 837 (s), 764 (m), 699 (s), 568 (m, Mo−O), 506 cm^−1^ (s); UV/Vis [CH_2_Cl_2_; *λ*_max_ (*ϵ*)]: 359 nm (8070 m ^−1^ cm^−1^), 547 nm (sh, 5020 m ^−1^ cm^−1^). HR‐MS: (ESI^+^) *m*/*z* [*M*]^+^ calcd for C_64_H_76_MoN_3_O_3_Si: 1060.4721, found: 1060.4729; HR‐MS: (ESI^−^) *m*/*z* [HB(C_6_F_5_)_3_]^−^ calcd for C_18_HBF_15_: 512.9940, found: 512.9877; Anal. calcd for C_82_H_77_BF_15_MoN_3_O_3_Si: C, 62.64; H, 4.94; N, 2.67; Found: C, 61.51; H, 4.67; N, 2.86.

**Synthesis of [Mo(OSiMe_3_)(N*t*Bu)L_2_][HB(C_6_F_5_)_3_] (3 c)**: For the synthesis of **3 c**, 3 equiv of trimethylsilyl chloride (10.7 μL, 0.03 mmol) were added to a solution of 1 equiv of **3 a** (50 mg, 0.03 mmol) in toluene (3 mL). The resulting reaction mixture was stirred at 50 °C for 12 h followed by removal of all volatiles. The residue was washed with cold pentane (2 mL) and dried in vacuo to obtain **3 c** as a purple solid (44 mg, 90 %). ^1^H NMR (CD_2_Cl_2_, 25 °C): *δ*=8.68 (s, 1 H, CH=N), 8.25 (s, 1 H, CH=N), 7.77 (d, 1 H, ArH), 7.64 (d, 1 H, ArH), 7.51 (d, 1 H, ArH), 7.31–7.18 (m, 3 H, Ph), 7.14 (d, 1 H, ArH), 7.08–6.95 (m, 5 H, Ph), 6.95–6.83 (m, 2 H, Ph), 4.03–3.10 (1:1:1:1 br q, 1 H, B‐H), 1.35 (s, 9 H, *t*Bu), 1.34 (s, 9 H, *t*Bu), 1.30 (s, 9 H, *t*Bu), 1.20 (s, 9 H, *t*Bu), 1.02 (s, 9 H, *t*Bu), 0.11 ppm (s, 9 H, SiMe_3_); ^13^C NMR (CD_2_Cl_2_, 25 °C, C‐F obscured): *δ*=170.59 (2x, C=N), 162.31, 157.82 (ArO), 151.71, 150.40, 147.96, 146.27, 138.74, 137.63 (*q*‐C), 134.41, 132.80, 131.28 (ArH), 129.75, 129.53 (Ph), 129.32 (ArH), 128.52, 128.04, 124.16, 123.11 (Ph), 122.23, 121.61 (*q*‐C), 79.13 (imido‐*q*‐*t*Bu), 35.96, 35.55, 35.07, 35.05 (*q*‐*t*Bu), 31.40, 31.37, 30.44, 30.16, 29.74 (*t*Bu), 2.11 ppm (SiMe_3_); ^19^F NMR (CD_2_Cl_2_, 25 °C): *δ*=−133.95 (m, 6 F, *o*‐F), −164.83 (t, 3 F, *p*‐F), −167.66 ppm (m, 6 F, *m*‐F); IR (ATR): $\tilde{\nu }$ =2960 (m, C−H), 2383 (br w, B−H), 1591 (w, C=N), 1548 (w), 1507 (m), 1460 (s), 1244 (m), 1181 (w), 1096 (s), 967 (s), 903 (s), 833 (s), 764 (m), 704 (w), 568 cm^−1^ (s, Mo−O); UV/Vis [CH_2_Cl_2_; *λ*_max_ (*ϵ*)]: 364 nm (7520 m ^−1^ cm^−1^), 526 nm (sh, 5310 m ^−1^ cm^−1^). Anal. calcd for C_67_H_71_BF_15_MoN_3_O_3_Si: C, 58.06; H, 5.16; N, 3.03; Found: C, 57.24; H, 4.95; N, 3.39.

**Synthesis of [Mo(OSiEt_3_)(N*t*Bu)L_2_][FB(C_6_F_5_)_3_] (4 a)**: For the synthesis of **4 a**, 1 equiv of **3 a** (60 mg, 0.04 mmol) was dissolved in CH_2_Cl_2_ (2 mL) and cooled to −35 °C. Subsequently, a precooled (−35 °C) solution of 1 equiv of [Ph_3_C][BF_4_] (14 mg, 0.04 mmol) in CH_2_Cl_2_ (1 mL) was added, resulting in a slight color change from deep purple to red–violet. The mixture was subsequently stored at −35 °C for 8 h and then stirred at room temperature for 3 h. Removal of all volatiles gave a violet solid that was washed twice with cold pentane (2×3 mL). Subsequent evaporation in vacuo provided **4 a** as violet solid (46 mg, 75 %). ^1^H NMR (CD_2_Cl_2_, 25 °C): *δ*=8.68 (s, 1 H, CH=N), 8.23 (s, 1 H, CH=N), 7.78 (d, 1 H, ArH), 7.64 (d, 1 H, ArH), 7.51 (d, 1 H, ArH), 7.31–7.18 (m, 3 H, Ph), 7.12 (d, 1 H, ArH), 7.08–6.98 (m, 5 H, Ph), 6.92–6.85 (m, 2 H, Ph), 1.36 (s, 9 H, *t*Bu), 1.35 (s, 9 H, *t*Bu), 1.30 (s, 9 H, *t*Bu), 1.21 (s, 9 H, *t*Bu), 1.03 (s, 9 H, *t*Bu), 0.85 (t, 9 H, SiEt_3_), 0.65–0.55 ppm (m, 6 H, SiEt_3_); ^13^C NMR (CD_2_Cl_2_, 25 °C, C‐F obscured): *δ*=170.79, 170.66 (C=N), 162.33, 157.84 (Ar‐O), 151.71, 150.44, 148.00, 146.34, 138.67, 137.78 (*q*‐C), 134.39, 132.98, 131.39 (ArH), 129.3 129.49 (Ph), 129.39 (ArH), 128.53, 128.12, 124.25, 123.25 (Ph), 122.35, 121.69 (*q*‐C), 79.28 (imido‐*q*‐*t*Bu), 36.00, 35.58, 35.07 (2x *q*‐*t*Bu), 31.41, 31.40, 30.61, 30.18, 29.88 (*t*Bu), 7.29, 7.19 ppm (SiEt_3_); ^19^F NMR (CD_2_Cl_2_, 25 °C): *δ*=−135.70 (p, 6 F, *o*‐F), −162.71 (t, 3 F, *p*‐F), −167.04 (m, 6 F, *m*‐F), −190.17 ppm (br s, 1 F, B−F); IR (ATR): $\tilde{\nu }$ =2960 (m, C−H), 1591 (w, C=N), 1549 (w), 1511 (w), 1462 (s), 1271 (w), 1243 (m), 1182 (w), 1089 (s), 962 (s), 888 (s), 842 (s), 765 (m), 681 (m), 571 cm^−1^ (m, Mo−O); Anal. calcd for C_70_H_76_BF_16_MoN_3_O_3_Si: C, 58.14; H, 5.30; N, 2.91; Found: C, 56.86; H, 5.28; N, 3.24.

**Synthesis of [Mo(OSiPh_3_)(N*t*Bu)L_2_][FB(C_6_F_5_)_3_] (4 b)**: For the synthesis of **4 b**, 1 equiv of **3 b** (27 mg, 0.02 mmol) was dissolved in CH_2_Cl_2_ (2 mL) and cooled to −35 °C. Subsequently, a precooled (−35 °C) solution of 1 equiv of [Ph_3_C][BF_4_] (6 mg, 0.02 mmol) in CH_2_Cl_2_ (1 mL) was added, resulting in a color change from violet to reddish. The mixture was stored at −35 °C for 8 h and then warmed to 5 °C where it was kept for one more hour. Removal of all volatiles gave a red‐violet solid that was subsequently extracted thrice with pentane (3×10 mL). The deep violet solution was concentrated to 10 mL and stored at −35 °C for 24 h to obtain **4 b** as dark microcrystalline solid (15 mg, 56 %). Single crystals suitable for X‐ray diffraction analysis were obtained via crystallization from a saturated pentane solution of **4 b** at −35 °C. ^1^H NMR (CD_2_Cl_2_, 25 °C): *δ*=8.69 (s, 1 H, CH=N), 8.14 (s, 1 H, CH=N), 7.79 (d, 1 H, ArH), 7.61 (d, 1 H, ArH), 7.53 (d, 1 H, ArH), 7.44–7.23 (m, 12 H, Ph), 7.167–7.08 (m, 6 H, Ph), 7.05 (d, 1 H, ArH), 7.01–6.92 (m, 5 H, Ph), 6.81–6.74 (m, 2 H, Ph), 1.34 (s, 9 H, *t*Bu), 1.33 (s, 9 H, *t*Bu), 1.21 (s, 9 H, *t*Bu), 0.93 (s, 9 H, *t*Bu), 0.80 ppm (s, 9 H, *t*Bu); ^11^B NMR (CD_2_Cl_2_, 25 °C): *δ*=−0.53 ppm (br d, ^1^J_(B−F)_=72.7 Hz); ^13^C NMR (CD_2_Cl_2_, 25 °C, C−F obscured): *δ*=171.11, 170.37 (C=N), 163.01, 157.25 (ArO), 151.47, 148.93, 148.65, 146.77, 138.97, 138.07 (*q*‐C), 136.27 (*o*‐SiPh_3_), 134.60 (*q*‐SiPh_3_), 134.49, 133.11, 131.50 (ArH), 130.91 (*p*‐SiPh_3_), 129.81, 129.29 (Ph), 129.08 (ArH), 128.50, 128.33 (Ph), 128.29 (*m*‐SiPh_3_), 124.85, 123.99 (Ph), 122.64, 122.12 (*q*‐C), 79.62 (imido‐*q*‐*t*Bu), 35.96, 35.75, 35.17, 35.08 (*q*‐*t*Bu), 31.42, 31.36, 30.55, 30.01, 29.76 ppm (*t*Bu); ^19^F NMR (CD_2_Cl_2_, 25 °C): *δ*=−135.66 (p, 6 F, *o*‐F), −162.68 (t, 3 F, *p*‐F), −167.00 (m, 6 F, *m*‐F), −190.61 ppm (br s, 1 F, B−F); IR (ATR): $\tilde{\nu }$ =2961 (m, C−H), 1590 (w, C=N), 1548 (w), 1510 (m), 1460 (s), 1243 (m), 1180 (m), 1087 (s), 962 (s), 891 (s), 838 (s), 765 (m), 699 (s), 571 (m, Mo−O), 505 cm^−1^ (s) HR‐MS: (ESI^+^) *m*/*z* [*M*]^+^ calcd for C_64_H_76_MoN_3_O_3_Si: 1060.4721, found: 1060.4718; HR‐MS: (ESI^−^) *m*/*z* [FB(C_6_F_5_)_3_]^−^ calcd for C_18_BF_16_: 530.9846, found: 530.9748; Anal. calcd for C_82_H_76_BF_16_MoN_3_O_3_Si: C, 61.93; H, 4.82; N, 2.80; Found: C, 61.54; H, 4.72; N, 2.80.

**Characterization of [Mo(OSiEt_3_)(N*t*Bu)L_2_][BnOB(C_6_F_5_)_3_] (Int 3 a′)**: For the characterization of **Int 3 a’**, 2 equiv of benzaldehyde (8 μL, 0.08 mmol) were added to a solution of 1 equiv of **3 a** (50 mg, 0.04 mmol) dissolved in CD_2_Cl_2_ (0.5 mL). NMR spectra were recorded after 1, 6 and 24 h (^1^H, ^19^F) and 24 h (^11^B). ^1^H NMR (CD_2_Cl_2_, 25 °C): *δ*=8.66 (s, 1 H, CH=N), 8.23 (s, 1 H, CH=N), 7.79 (d, 1 H, ArH), 7.64 (d, 1 H, ArH), 7.50 (d, 1 H, ArH), 7.42–7.34 (m, 2 H, Ph), 7.29–7.18 (m, 5 H, Ph),7.14–6.99 (m, 7 H, Ph+ArH), 6.93–6.85 (m, 2 H, Ph), 4.25 (s, 2 H, CH_2_), 1.37 (s, 9 H, *t*Bu), 1.36 (s, 9 H, *t*Bu), 1.30 (s, 9 H, *t*Bu), 1.21 (s, 9 H, *t*Bu), 1.03 (s, 9 H, *t*Bu), 0.86 (t, 9 H, SiEt_3_), 0.66–0.56 ppm (m, 6 H, SiEt_3_); ^11^B NMR (CD_2_Cl_2_, 25 °C): *δ*=−2.68 ppm (s); ^19^F NMR (CD_2_Cl_2_, 25 °C): *δ*=−133.97 (m, 6 F, *o*‐F), −163.82 (t, 3 F, *p*‐F), −167.23 ppm (m, 6 F, *m*‐F).

## Conflict of interest

The authors declare no conflict of interest.

## Supporting information

As a service to our authors and readers, this journal provides supporting information supplied by the authors. Such materials are peer reviewed and may be re‐organized for online delivery, but are not copy‐edited or typeset. Technical support issues arising from supporting information (other than missing files) should be addressed to the authors.

Supplementary
