# Supplementary material for: Heterolytic Si−H Bond Cleavage at a Molybdenum‐Oxido‐Based Lewis Pair
Source: Chemistry. 2018 Apr 27;24(28):7149–60. doi: 10.1002/chem.201800226 (PMC6001527; doi:10.1002/chem.201800226)
Supplement: Supplementary file 1 — Supplementary [file CHEM-24-7149-s001.pdf]

CHEMISTRY
A European Journal

Supporting Information

Heterolytic Si–H Bond Cleavage at a Molybdenum-Oxido-Based Lewis Pair

Niklas Zwettler, Simon P. Walg, Ferdinand Belaj, and Nadia C. Mösch-Zanetti*[a]

chem_201800226_sm_miscellaneous_information.pdf

Content

Supplementary figures **3**

Figure S1. ^{19}F NMR spectra of the concentration dependent equilibrium of **2** (●) with $MeCN{\cdot}B(C_6F_5)_3$ (□) in C_6D_6. 3

Figure S2. IR spectra of compounds **1**,[1] **2** and **3a** in the region from 350 to 3500 cm^{-1}. ... 4

Figure S3. Scan rate dependence of the redox couples assigned to Mo(VI)/Mo(V) for complex **2** (left), **3a** (middle) and **3c** (right). 4

Figure S4. ^{19}F NMR spectra of the reaction of **3a** with benzaldehyde in C_6D_6. 4

Figure S5. ^{1}H NMR spectra of the reaction of **3a** with benzaldehyde in C_6D_6. 5

Figure S6. ^{19}F NMR spectra of the reaction of **3a** with benzaldehyde in CD_2Cl_2. 5

Figure S7. ^{1}H NMR spectra of the reaction of **3a** with benzaldehyde in CD_2Cl_2. 6

Figure S8. ^{11}B NMR spectrum of **Int 3a'** from the reaction of **3a** with benzaldehyde in CD_2Cl_2 after 24 h. 6

Figure S9. Superimposed ^{1}H NMR spectra of **Int 3a'** and **Int 3a'-d$_1$**. 7

Figure S10. ^{13}C NMR spectrum of **Int 3a'-d$_1$** 7

NMR and MS spectra of the compounds **8**

Figure S11. ^{1}H NMR spectrum of complex **2** in CD_2Cl_2. 8

Figure S12. ^{11}B NMR spectrum of complex **2** in CD_2Cl_2. 8

Figure S13. ^{13}C NMR spectrum of complex **2** in CD_2Cl_2. 9

Figure S14. ^{19}F NMR spectrum of complex **2** in CD_2Cl_2. 9

Figure S15. ^{1}H NMR spectrum of complex **3a** in CD_2Cl_2. 10

Figure S16. ^{11}B NMR spectrum of complex **3a** in CD_2Cl_2. 10

Figure S17. ^{11}B NMR spectrum of complex **3a-d$_1$** in CD_2Cl_2. 11

Figure S18. ^{13}C NMR spectrum of complex **3a** in CD_2Cl_2. 11

Figure S19. ^{19}F NMR spectrum of complex **3a** in CD_2Cl_2. 12

Figure S20. ^{1}H NMR spectrum of complex **3b** in CD_2Cl_2. 12

Figure S21. ^{11}B NMR spectrum of complex **3b** in CD_2Cl_2. 13

Figure S22. ^{13}C NMR spectrum of complex **3b** in CD_2Cl_2. 13

Figure S23. ^{19}F NMR spectrum of complex **3b** in CD_2Cl_2. 14

Figure S24. ^{1}H NMR spectrum of complex **3c** in CD_2Cl_2. 14

Figure S25. ^{13}C NMR spectrum of complex **3c** in CD_2Cl_2. 15

Figure S26. ^{19}F NMR spectrum of complex **3c** in CD_2Cl_2. 15

Figure S27. ^{1}H NMR spectrum of complex **4a** in CD_2Cl_2. 16

Figure S28. ^{13}C NMR spectrum of complex **4a** in CD_2Cl_2. 16

Figure S29. ^{19}F NMR spectrum of complex **4a** in CD_2Cl_2. 17

Figure S30. ^{1}H NMR spectrum of complex **4b** in CD_2Cl_2. 17

Figure S31. ^{11}B NMR spectrum of complex **4b** in CD_2Cl_2. 18

Figure S32. ^{13}C NMR spectrum of complex **4b** in CD_2Cl_2. 18

Figure S33. ^{19}F NMR spectrum of complex **4b** in CD_2Cl_2. 19

Figure S34. ESI-MS (positive mode) spectrum for **3a**, showing the peak for $[Mo(OSiEt_3)(NtBu)]^+$ with the correct isotope pattern. 19

Figure S35. ESI-MS (negative mode) spectrum for **3a**, showing peaks for $[HB(C_6F_5)_3]^-$ and $[HOB(C_6F_5)_3]^-$....20

Figure S36. ESI-MS (positive mode) spectrum for **3b**, showing the peak for $[Mo(OSiPh_3)(NtBu)]^+$ with the correct isotope pattern. 20

Figure S37. ESI-MS (negative mode) spectrum for **3b**, showing the peak for $[HB(C_6F_5)_3]^-$. 21

Figure S38. ESI-MS (positive mode) spectrum for **4b**, showing the peak for $[Mo(OSiPh_3)(NtBu)]^+$ with the correct isotope pattern. 21

Figure S39. ESI-MS (negative mode) spectrum for **4b**, showing a peak for $[FB(C_6F_5)_3]^-$, .22

Electrochemistry data of the compounds 22

Figure S40. Full sweep-width cyclovoltammogram of complex **1**[1] in MeCN....22

Figure S41. Full sweep-width cyclovoltammogram of complex **2** in CH_2Cl_2. 23

Figure S42. Full sweep-width cyclovoltammogram of complex **3a** in MeCN. 23

Figure S43. Full sweep-width cyclovoltammogram of complex **3b** in MeCN. 23

Figure S44. Full sweep-width cyclovoltammogram of complex **3c** in MeCN. 24

Crystallographic data for 2 and 4b....25

Crystal structure determination of 2.....25

Figure S45. Stereoscopic ORTEP[5] plot of **2** 25

Table S1. Crystal data and structure refinement for **2** (CCDC deposition number: 1574332) 26

Table S2. Selected bond lengths [Å] and angles [°] for **2**. 27

Crystal structure determination of 4b. 27

Figure S46. Stereoscopic ORTEP[5] plot of the complex cation found in **4b** 28

Figure S47. Stereoscopic ORTEP[5] plot of **4b** 28

Table S3. Crystal data and structure refinement for **4b** (CCDC deposition number: 1574333) 29

Table S4. Selected bond lengths [Å] and angles [°] for **4b**. 30

References....30

Supplementary figures

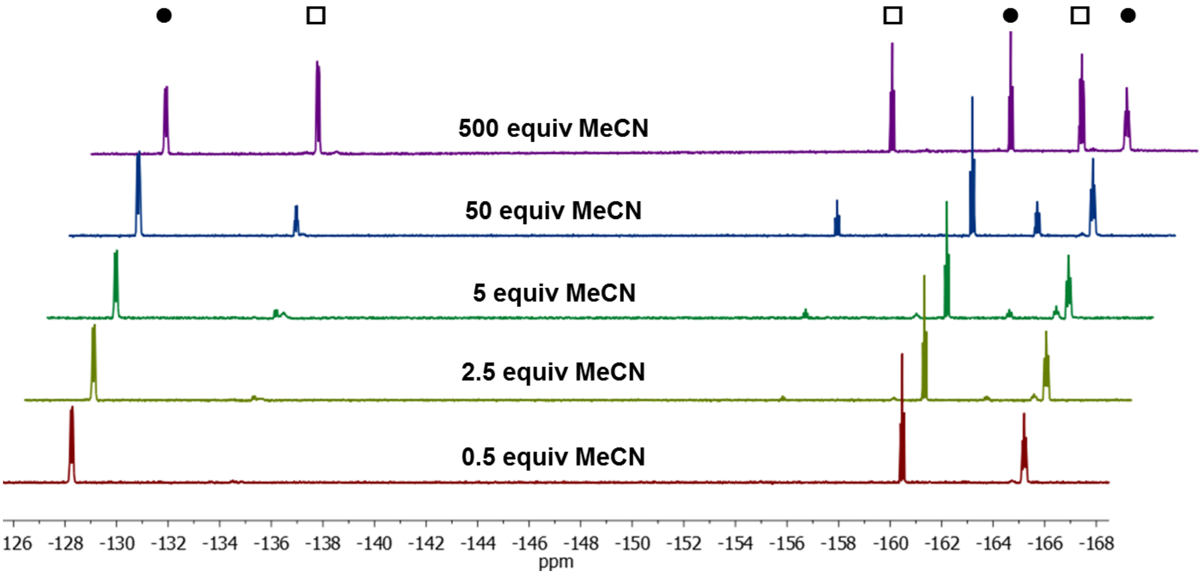

Figure S1. ^{19}F NMR spectra of the concentration dependent equilibrium of **2** (●) with $MeCN{\cdot}B(C_6F_5)_3$ (☐) in C_6D_6.

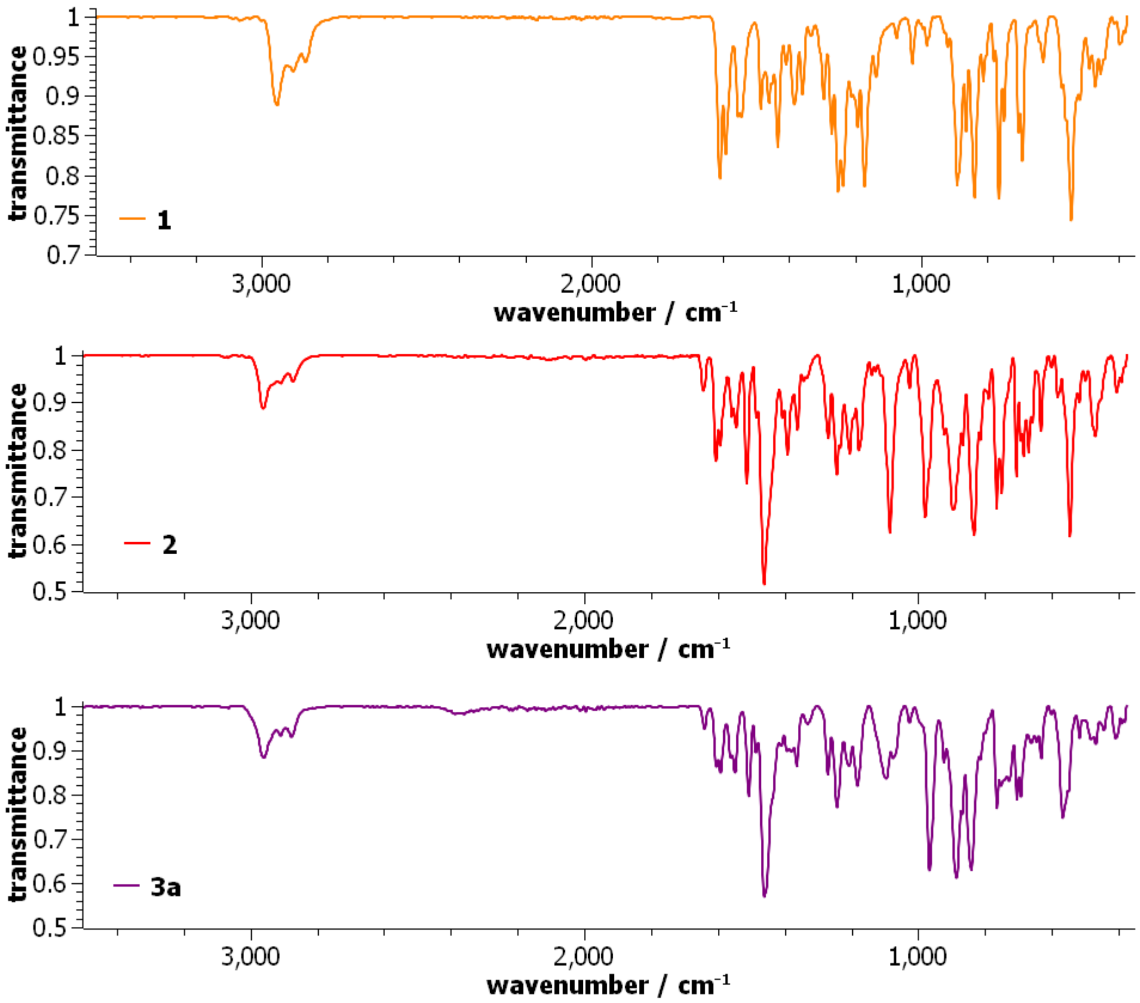

Figure S2. IR spectra of compounds **1**,[1] **2** and **3a** in the region from 350 to 3500 cm^{-1}.

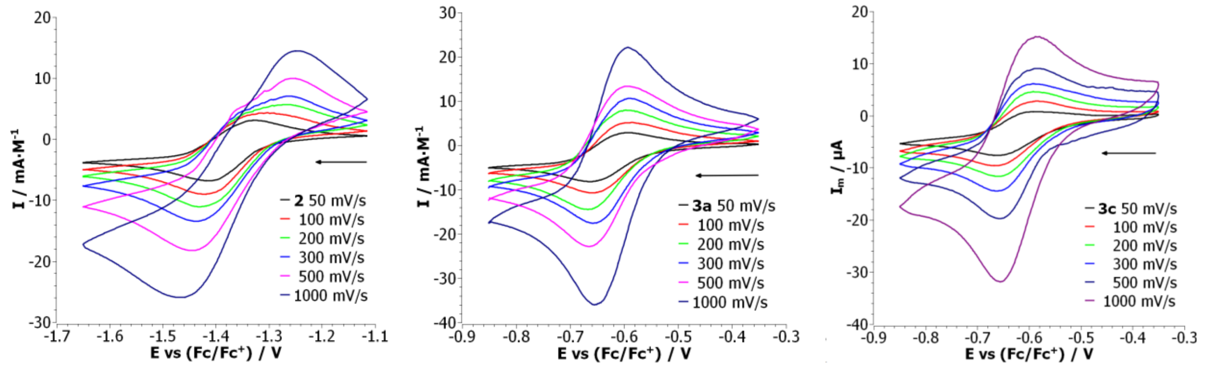

Figure S3. Scan rate dependence of the redox couples assigned to Mo(VI)/Mo(V) for complex **2** (left), **3a** (middle) and **3c** (right).

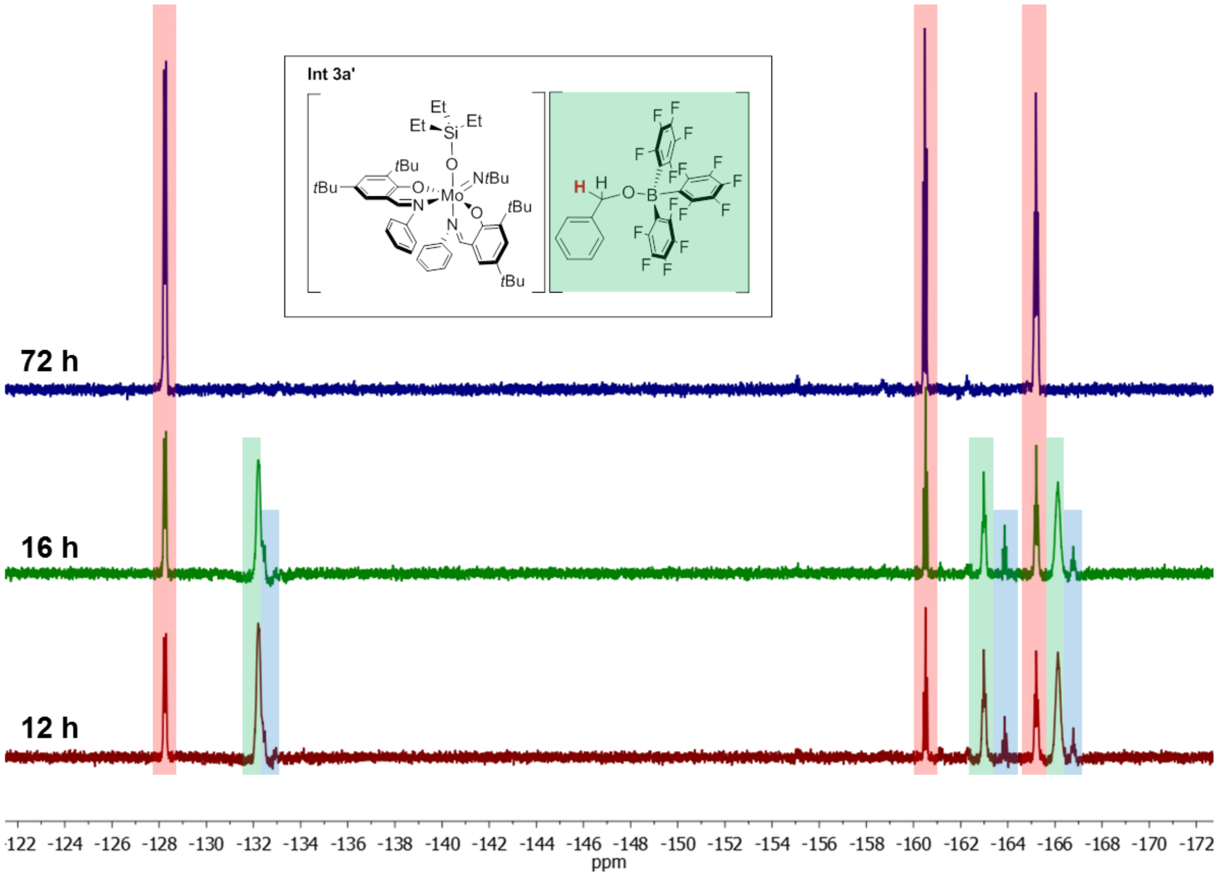

Figure S4. ^{19}F NMR spectra of the reaction of **3a** with benzaldehyde in C_6D_6. The red boxes mark the resonances for the Lewis adduct **2**, the blue boxes mark residual $[HB(C_6F_5)_3]^-$ from **3a** and the resonances in the green boxes correspond to the anion of the intermediate species **Int 3a'**.

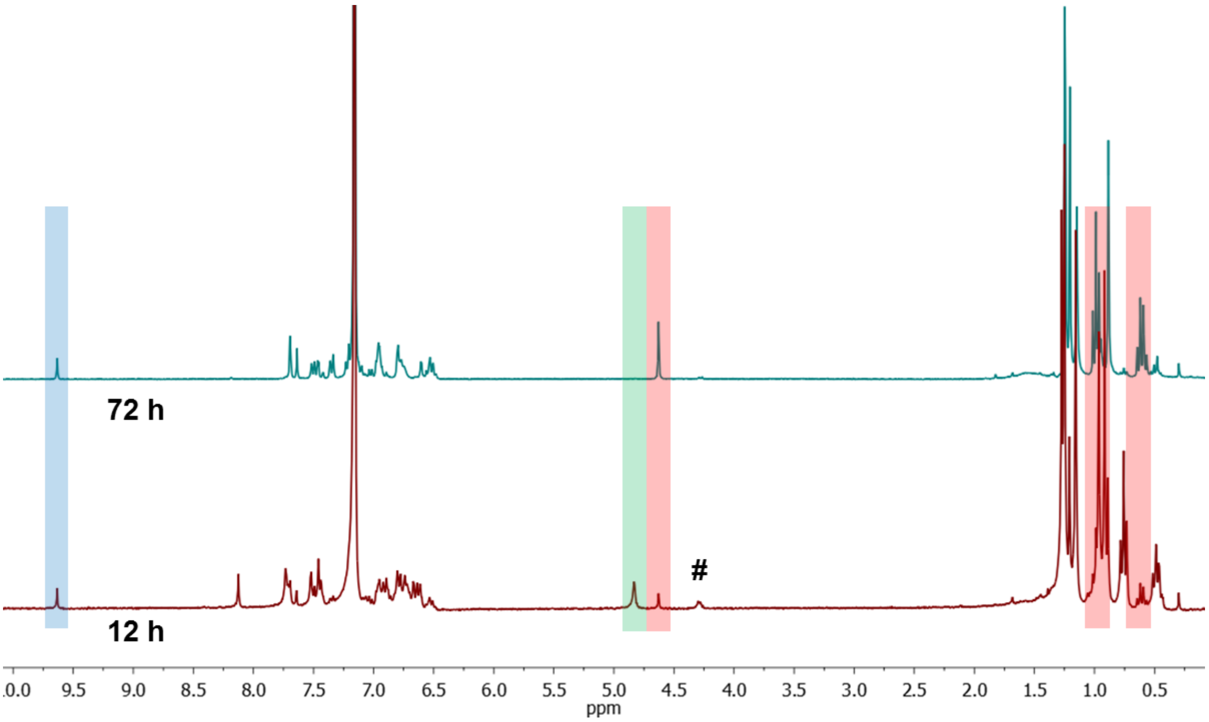

Figure S5. ^{1}H NMR spectra of the reaction of **3a** with benzaldehyde in C_6D_6. The red boxes correspond to Bn-$OSiEt_3$, the blue box refers to residual benzaldehyde and the # symbols correspond to Bn–OH impurity. The resonance in the green box corresponds to the intermediate species **Int 3a'**.

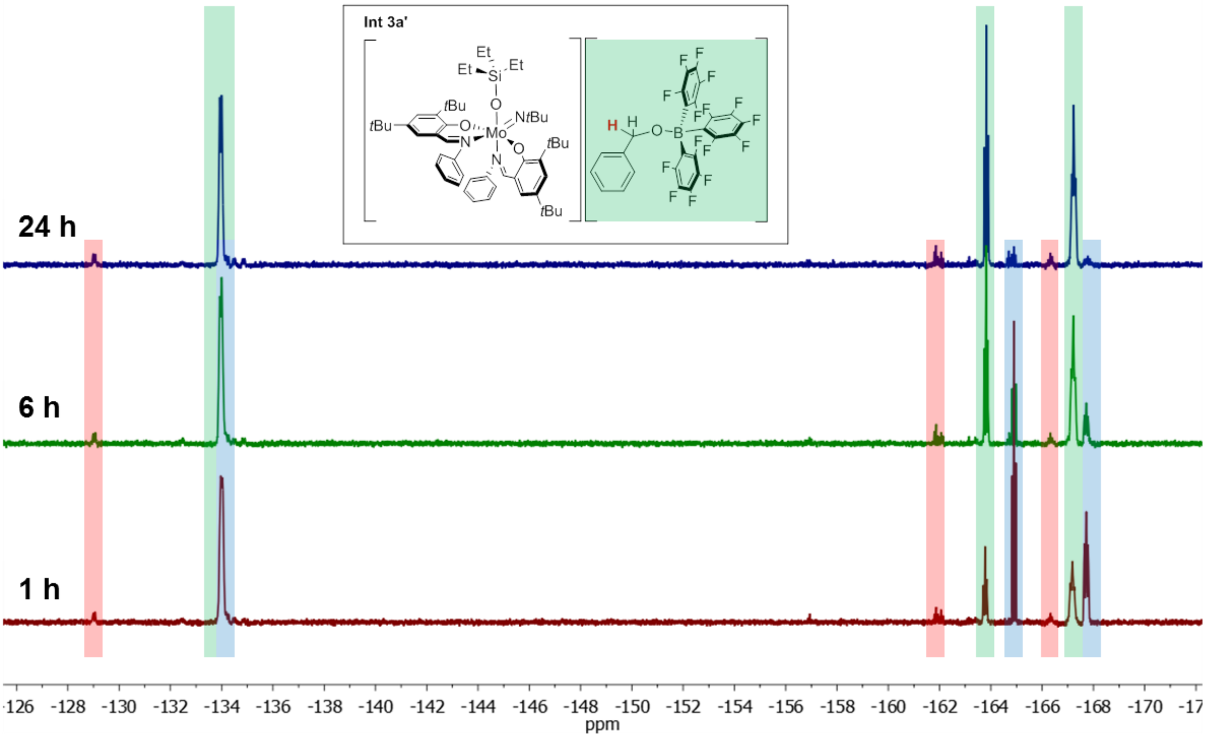

Figure S6. ^{19}F NMR spectra of the reaction of **3a** with benzaldehyde in CD_2Cl_2. The blue boxes mark the resonances for the $[HB(C_6F_5)_3]^-$ anion of the initial complex **3a** and the resonances in the green boxes correspond to the intermediate species **Int 3a'**, consistent to literature.[2] Lewis adduct **2** is only formed in minute quantities, marked with the red boxes.

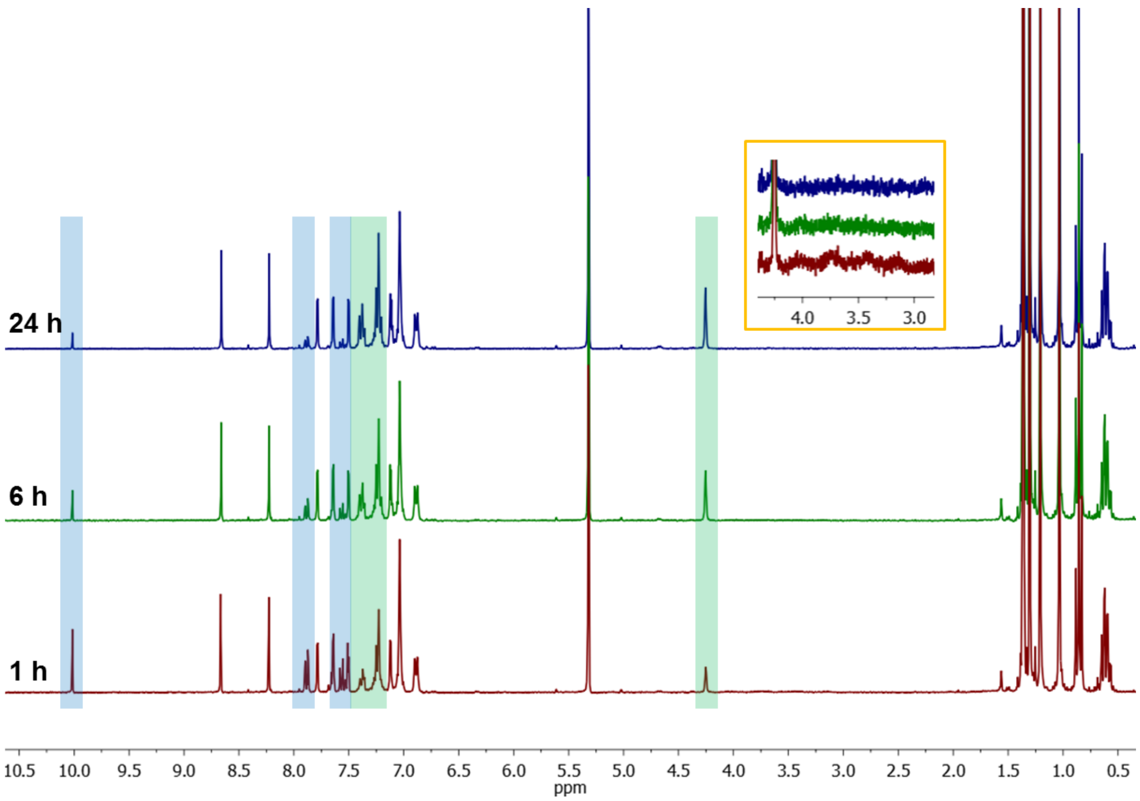

Figure S7. ^{1}H NMR spectra of the reaction of **3a** with benzaldehyde in CD_2Cl_2. The resonances in the green boxes correspond to the intermediate species **Int 3a'**, the blue boxes refer to residual benzaldehyde and the yellow box shows the decrease of the B–H resonance. No formation of complex **2** or Bn–$OSiEt_3$ is observed within 24 h of reaction time, after 72 h, the conversion is ~ 5%.

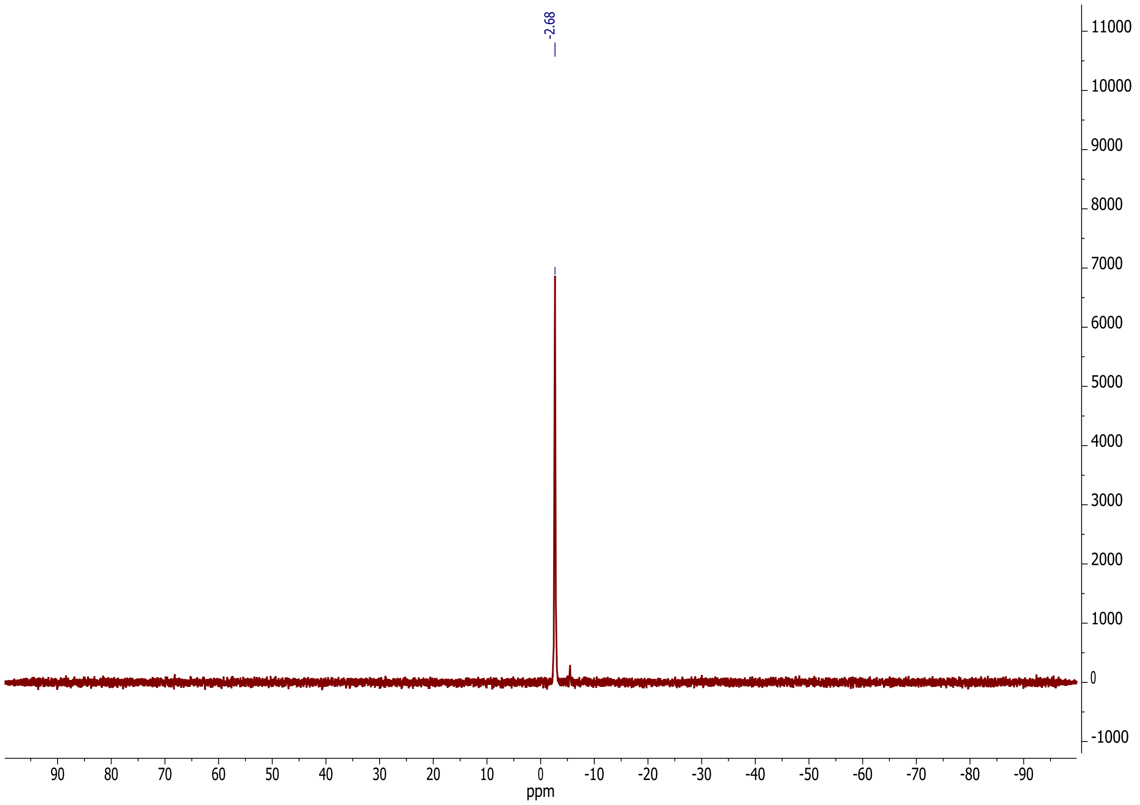

Figure S8. ^{11}B NMR spectrum of **Int 3a'** from the reaction of **3a** with benzaldehyde in CD_2Cl_2 after 24 h.

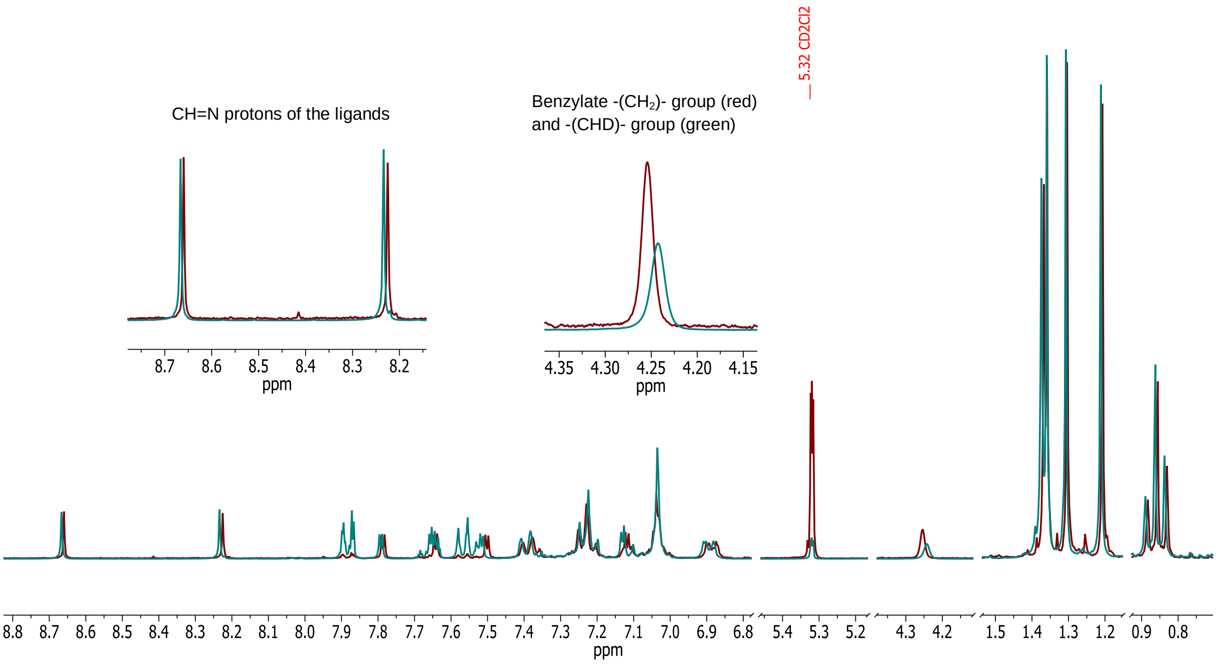

Figure S9. Superimposed ^{1}H NMR spectra of **Int 3a'** and **Int 3a'-d$_1$** showing the different peak areas for the benzylate methylene group; Red: **Int 3a'**, -(CH_2)- group integrates for two protons, Green: **Int 3a'-d$_1$**, -(CHD)- group integrates for one proton. Additional signals in the spectrum of **Int 3a'-d$_1$** correspond to excess benzaldehyde.

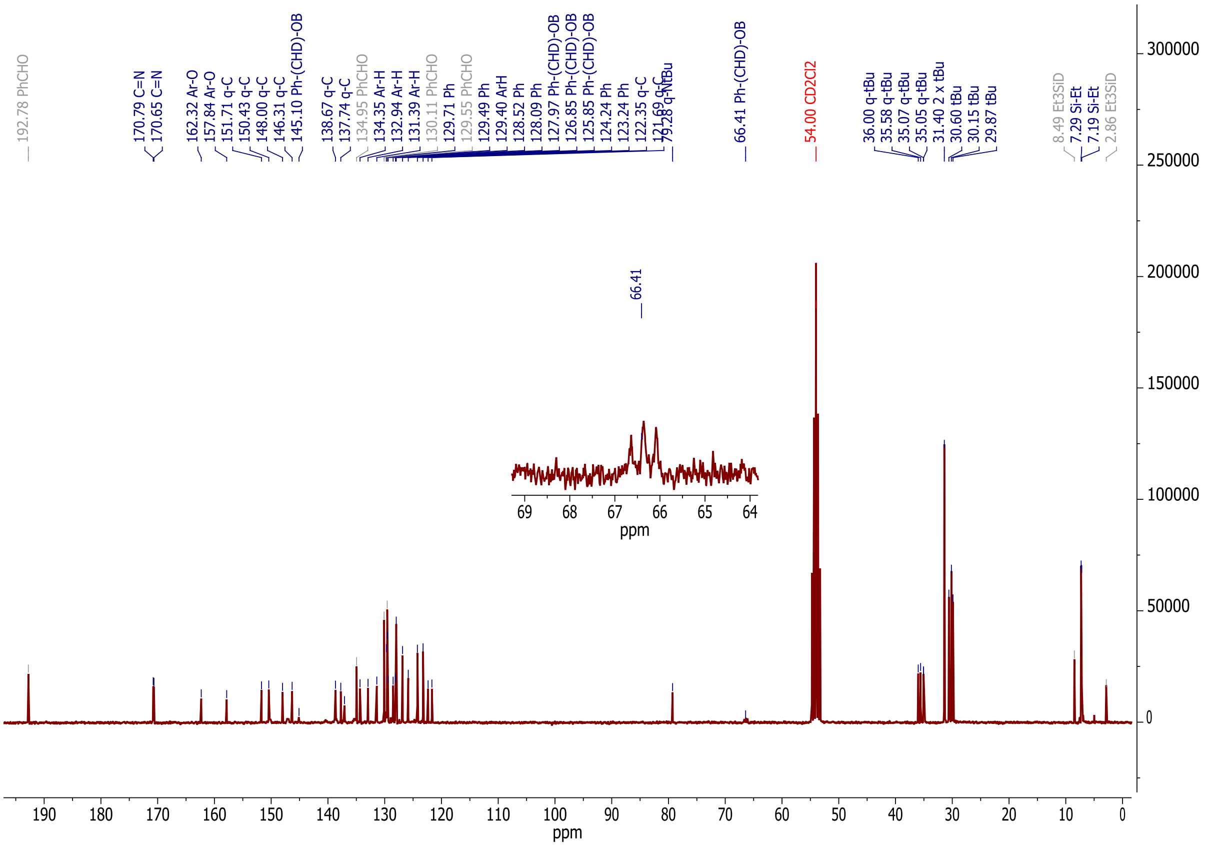

Figure S10. ^{13}C NMR spectrum of **Int 3a'-d$_1$** showing the characteristic triplet for the -(CHD)- group arising from $^{1}J_{(C\text{-}D)}$ coupling. The mixture also contains excess Et_3SiD and PhCHO as a result of *in situ* measurements.

NMR and MS spectra of the compounds

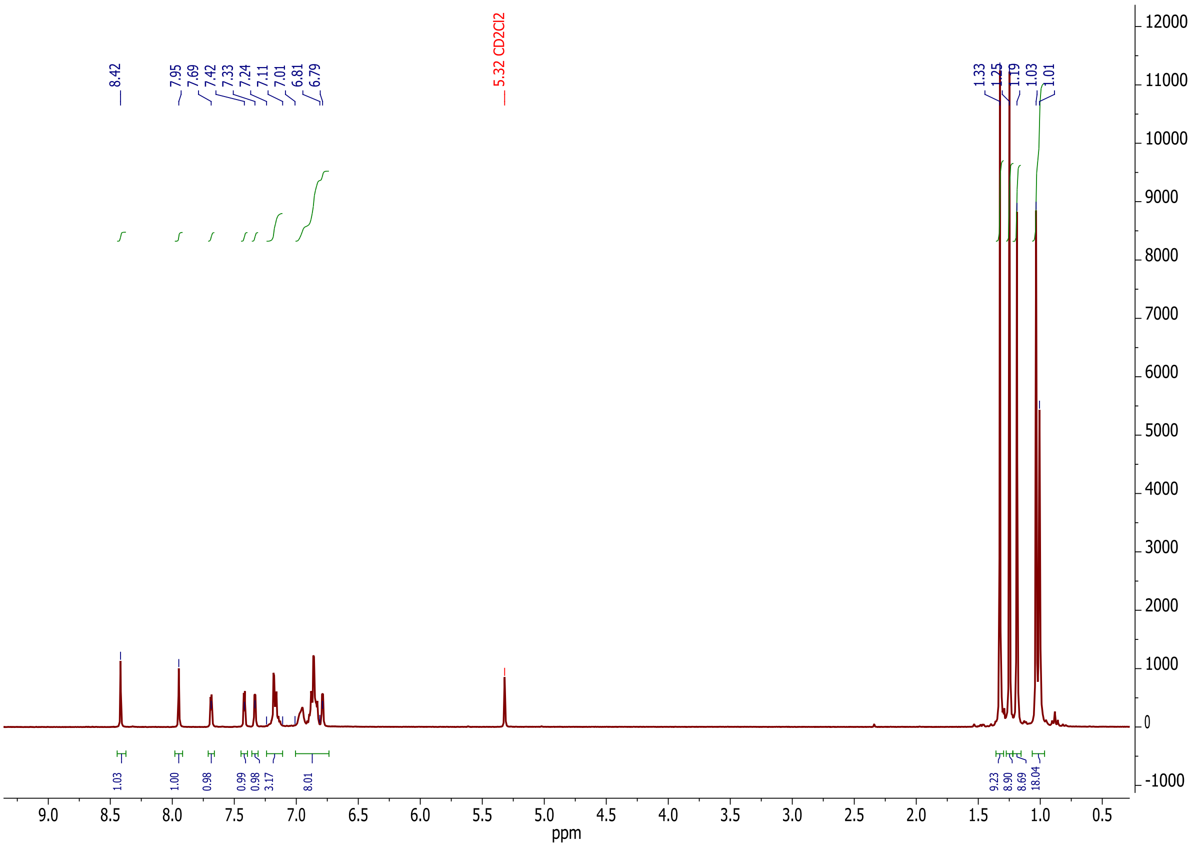

Figure S11. ^{1}H NMR spectrum of complex **2** in CD_2Cl_2.

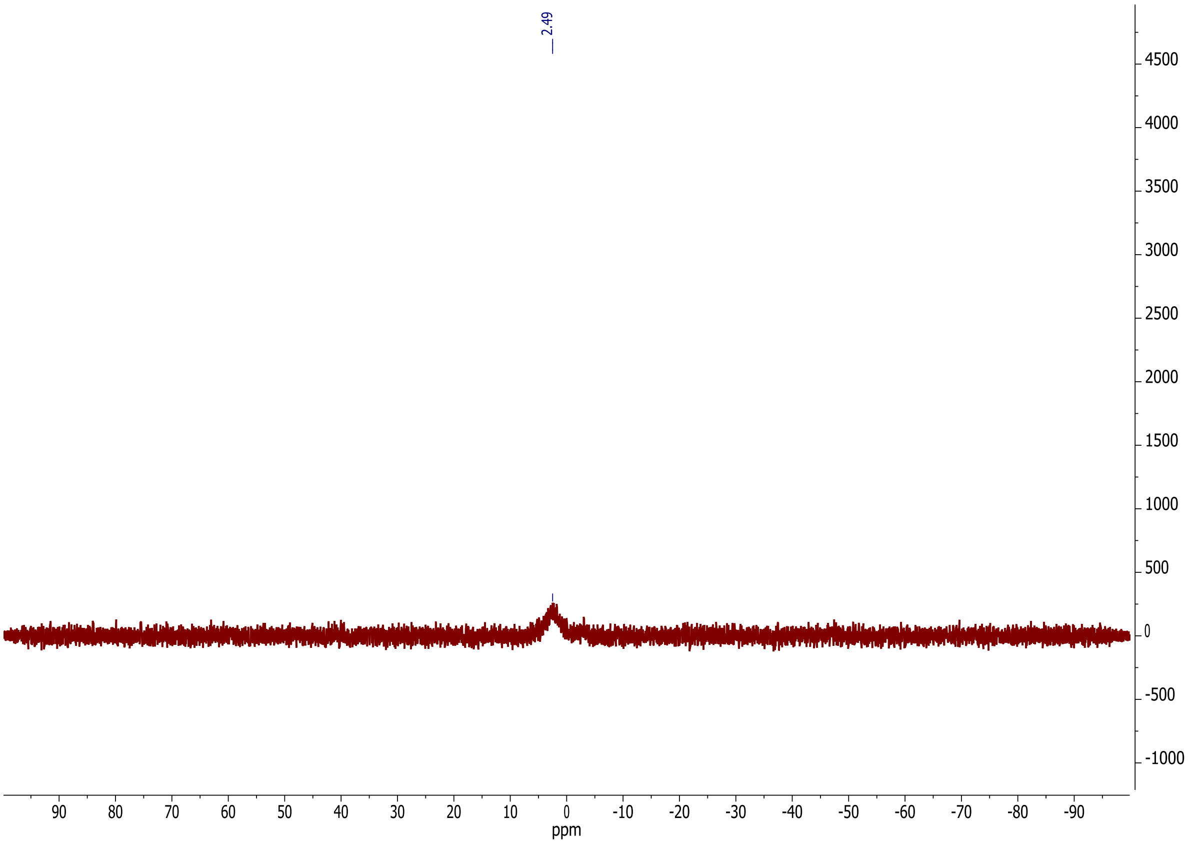

Figure S12. ^{11}B NMR spectrum of complex **2** in CD_2Cl_2.

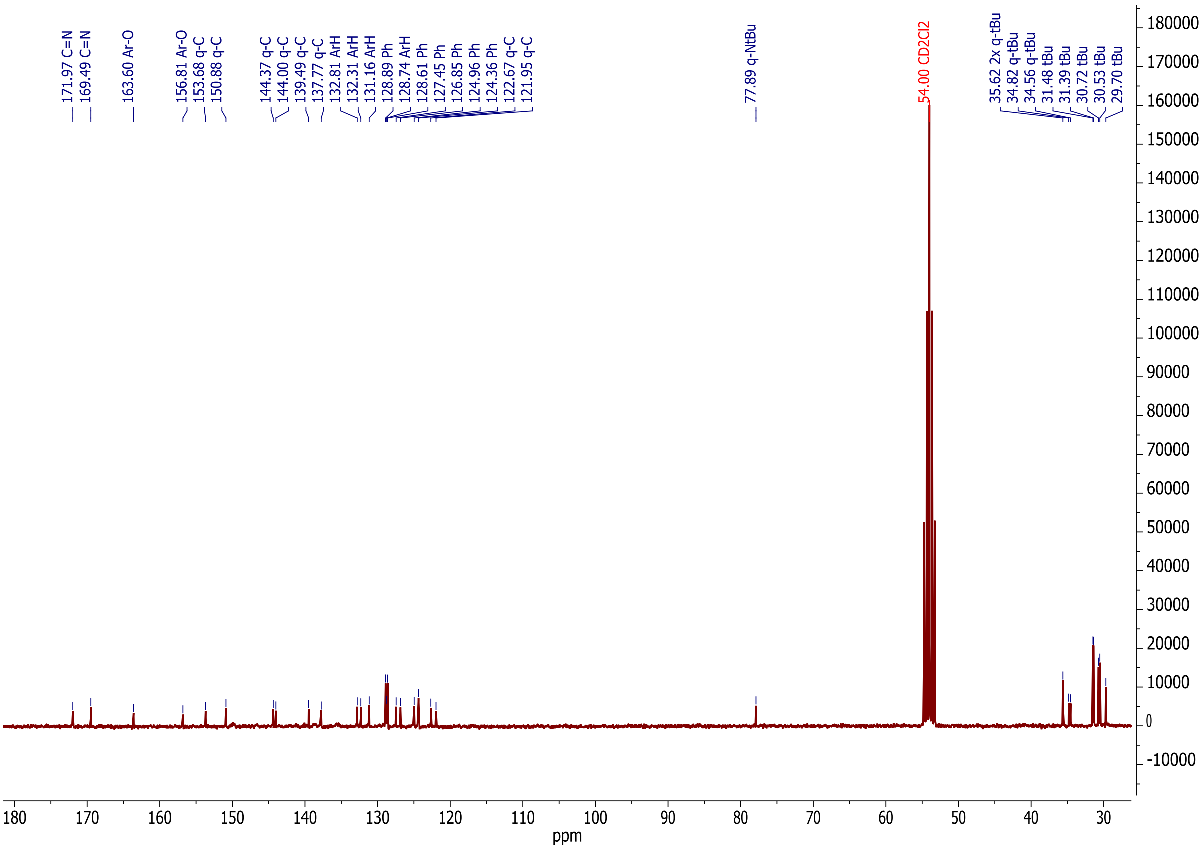

Figure S13. ^{13}C NMR spectrum of complex **2** in CD_2Cl_2.

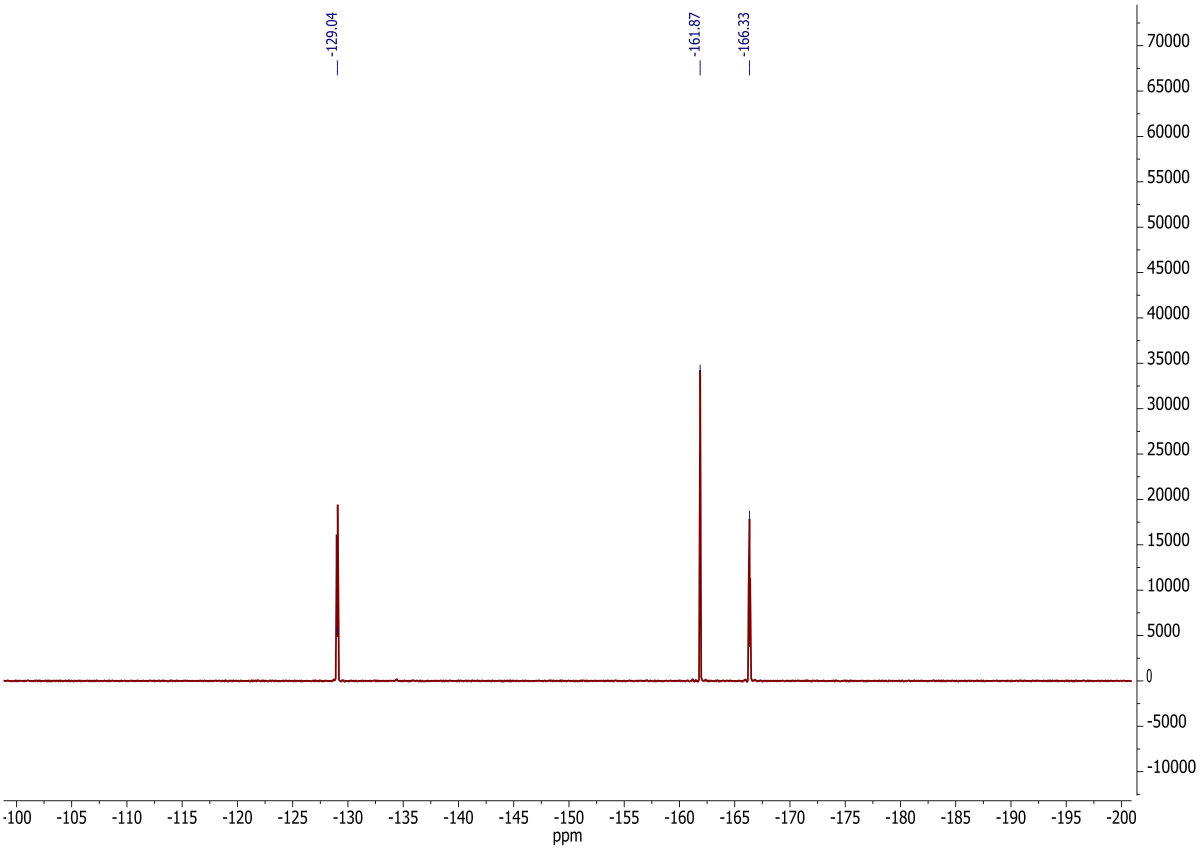

Figure S14. ^{19}F NMR spectrum of complex **2** in CD_2Cl_2.

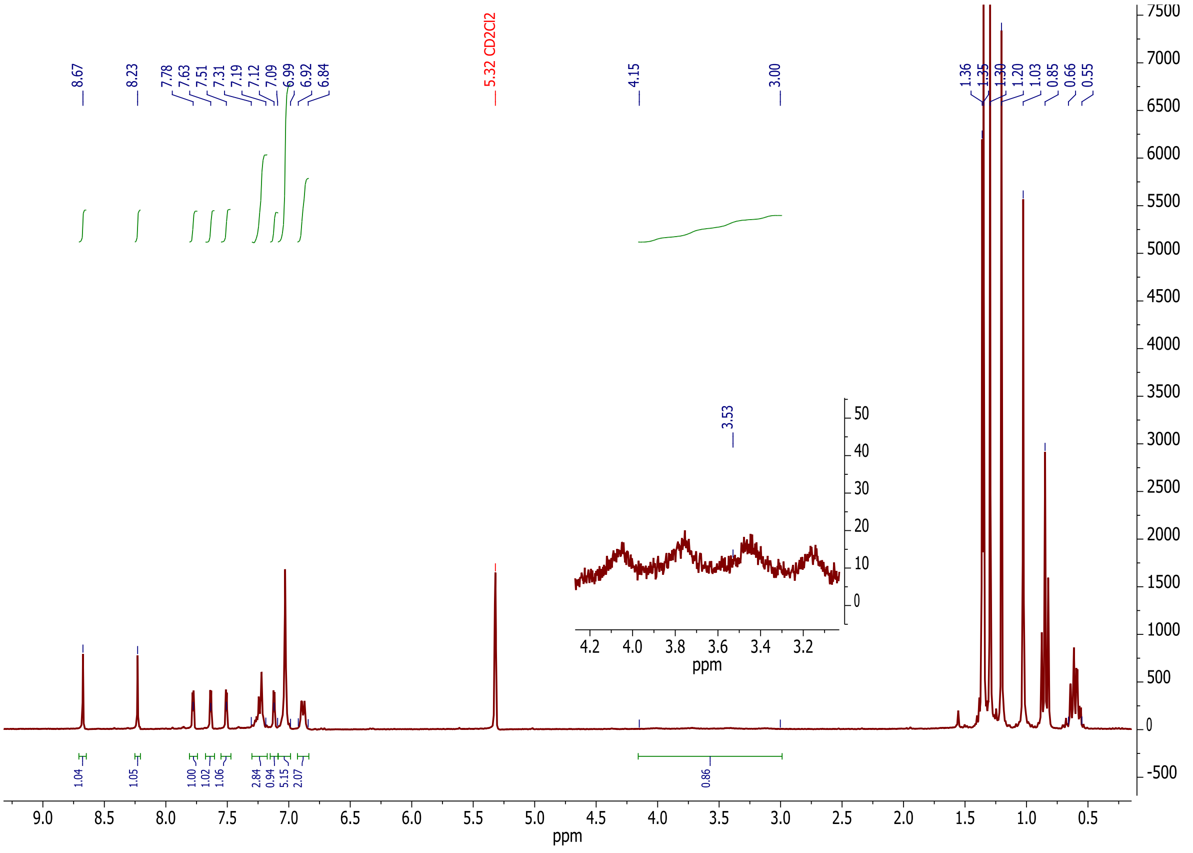

Figure S15. ^{1}H NMR spectrum of complex **3a** in CD_2Cl_2.

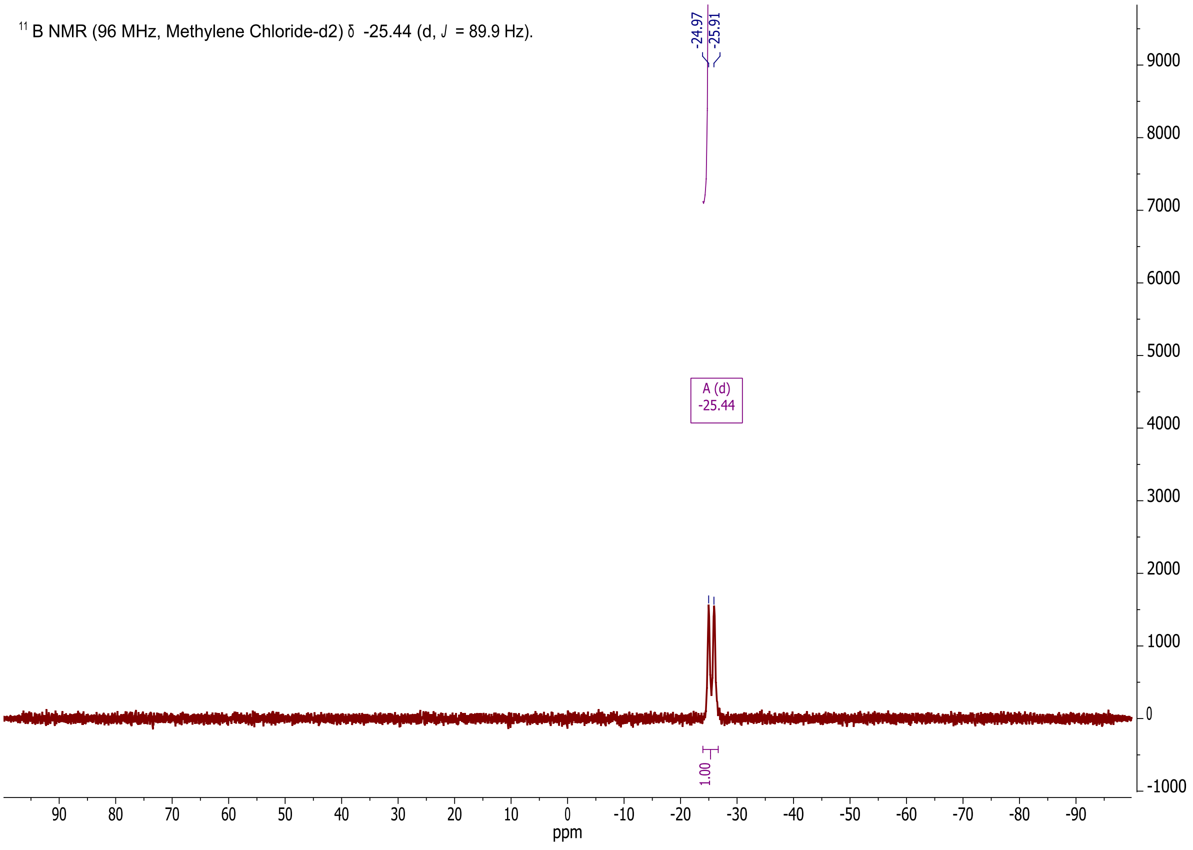

Figure S16. ^{11}B NMR spectrum of complex **3a** in CD_2Cl_2.

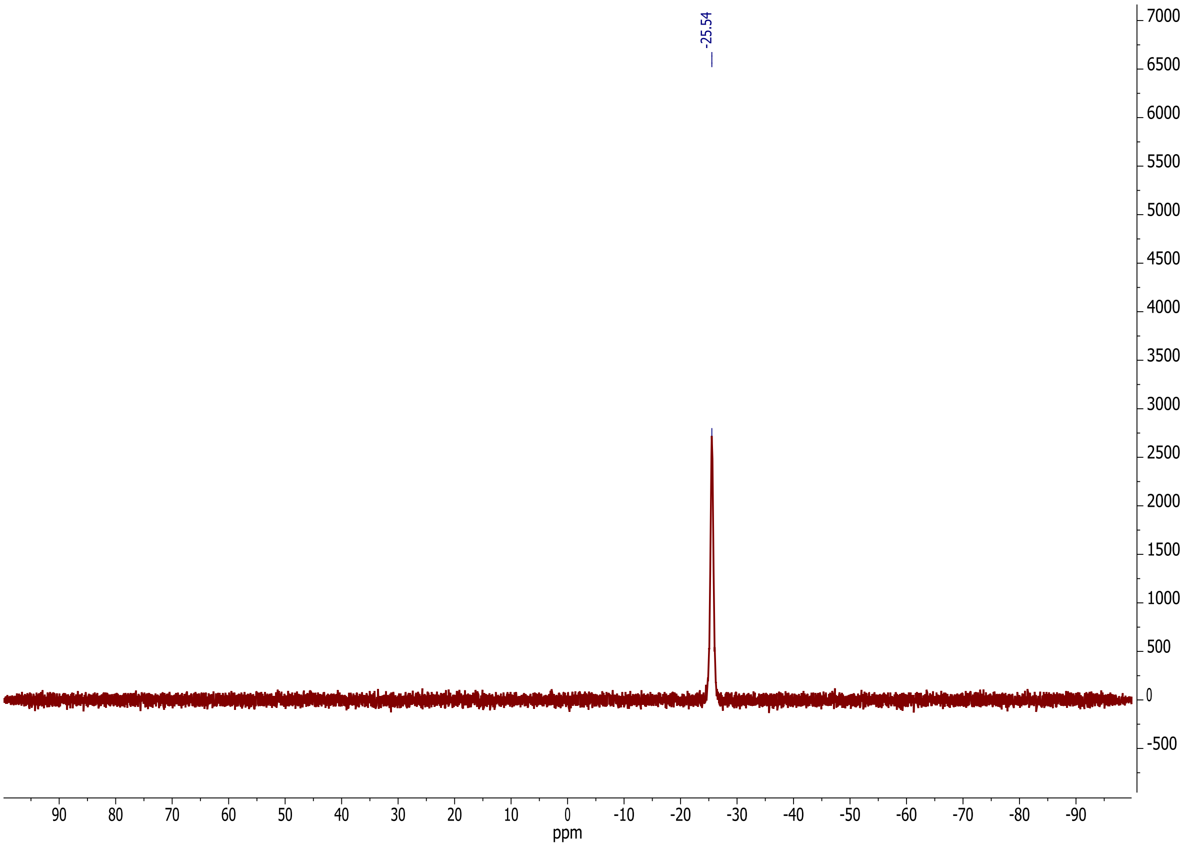

Figure S17. ^{11}B NMR spectrum of complex **3a-d$_1$** in CD_2Cl_2.

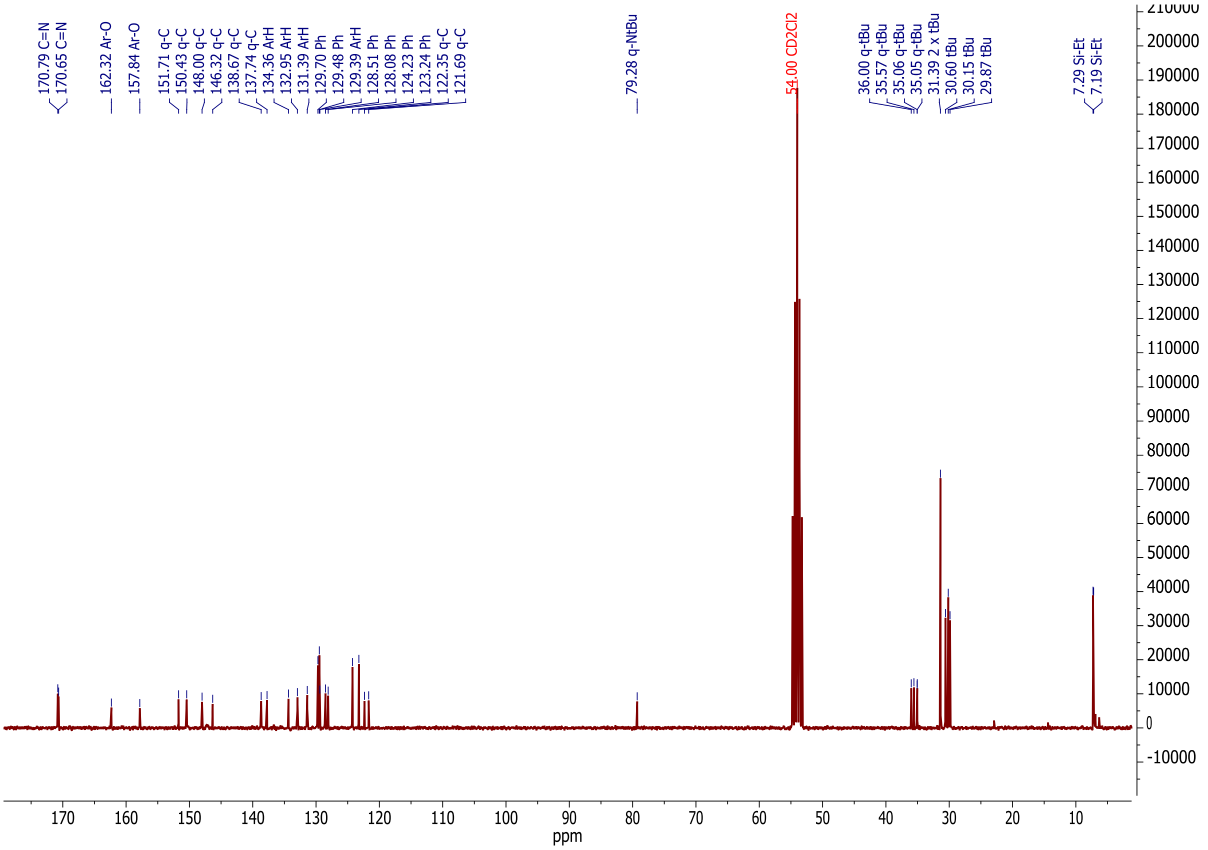

Figure S18. ^{13}C NMR spectrum of complex **3a** in CD_2Cl_2.

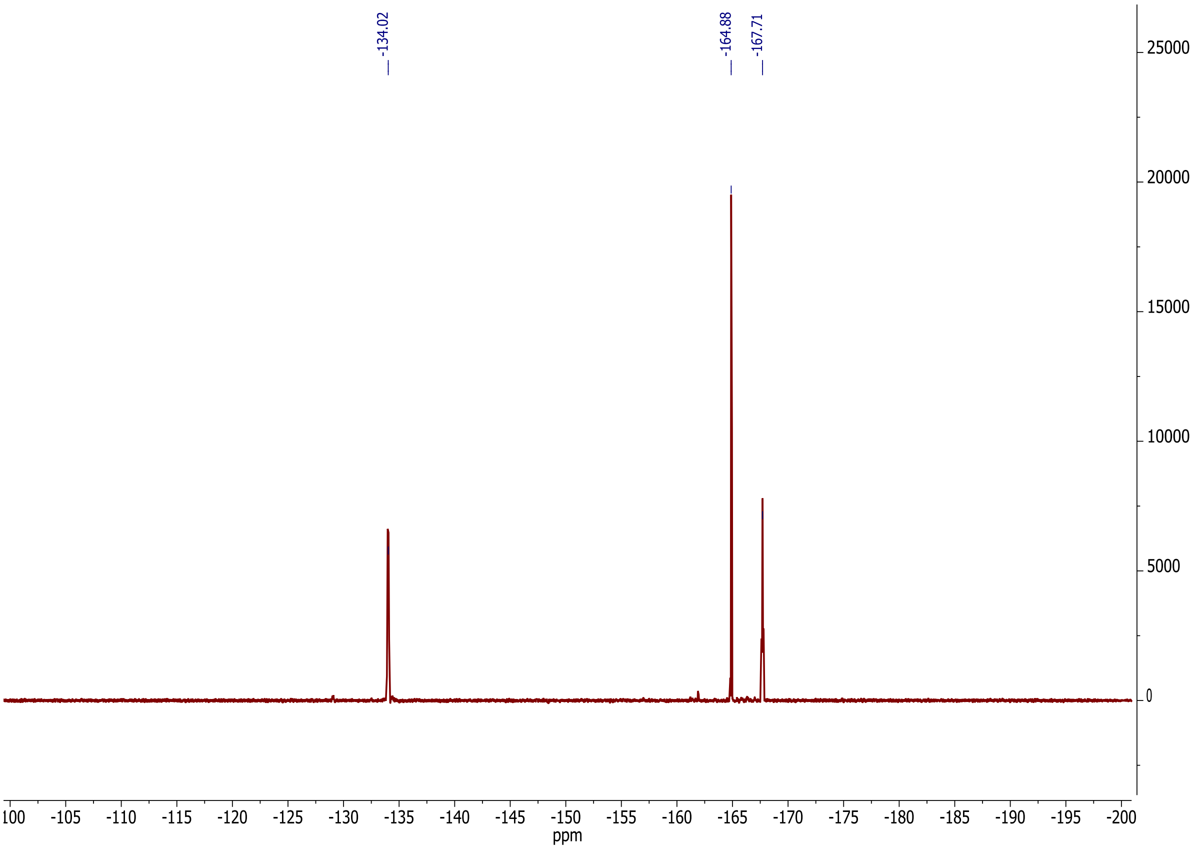

Figure S19. ^{19}F NMR spectrum of complex **3a** in CD_2Cl_2.

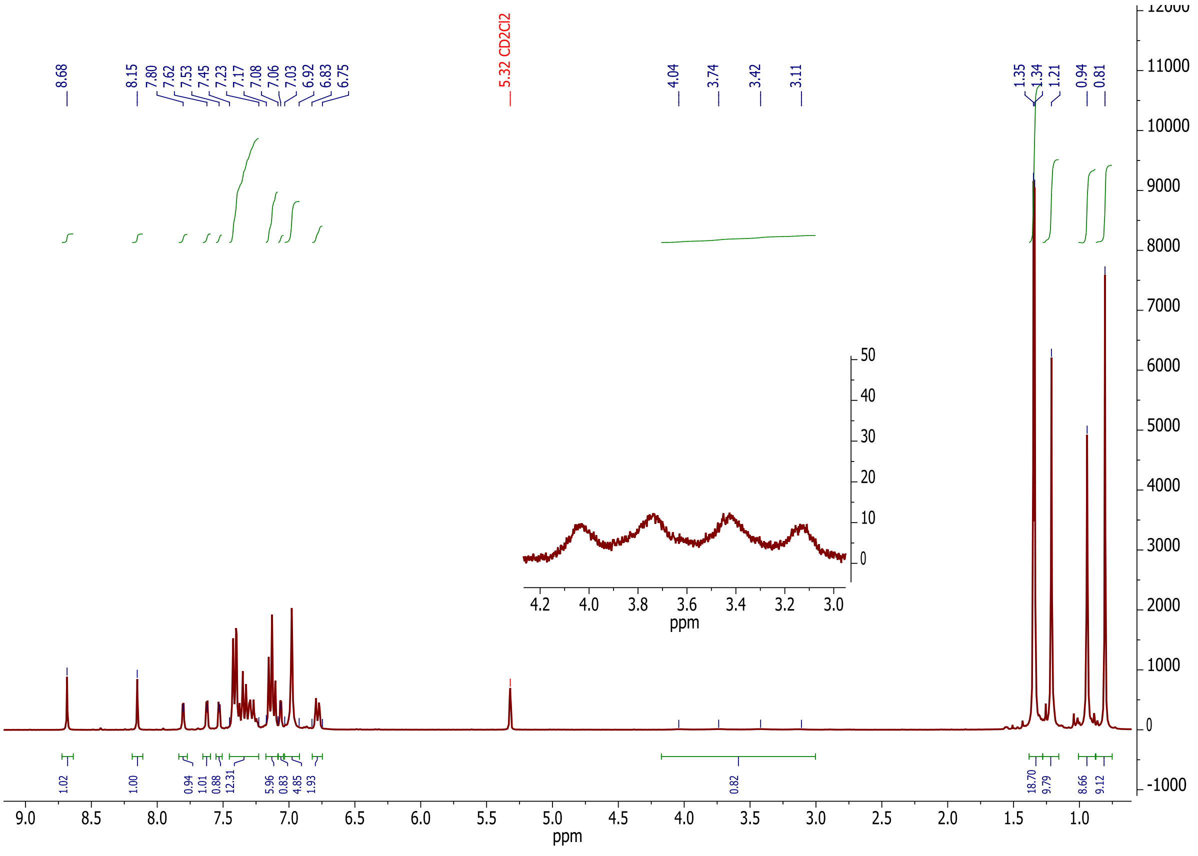

Figure S20. ^{1}H NMR spectrum of complex **3b** in CD_2Cl_2.

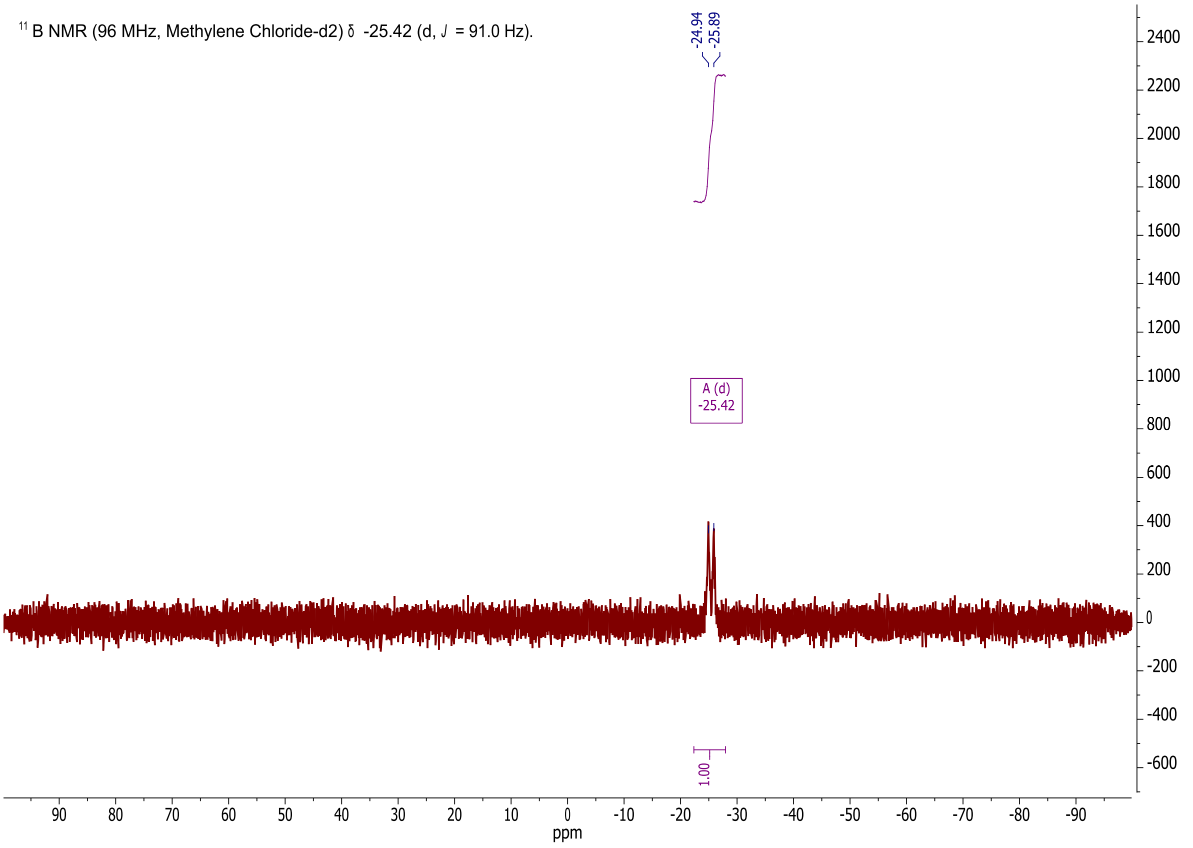

Figure S21. ^{11}B NMR spectrum of complex **3b** in CD_2Cl_2.

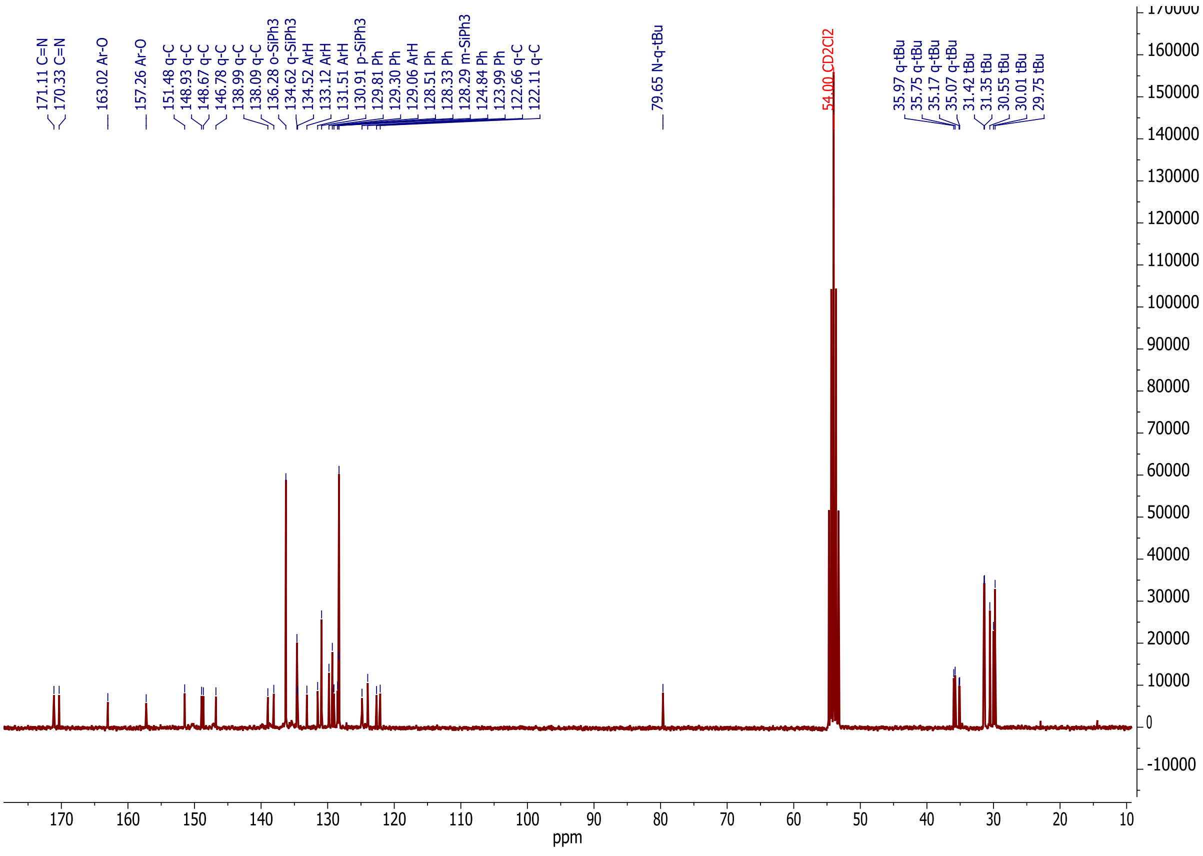

Figure S22. ^{13}C NMR spectrum of complex **3b** in CD_2Cl_2.

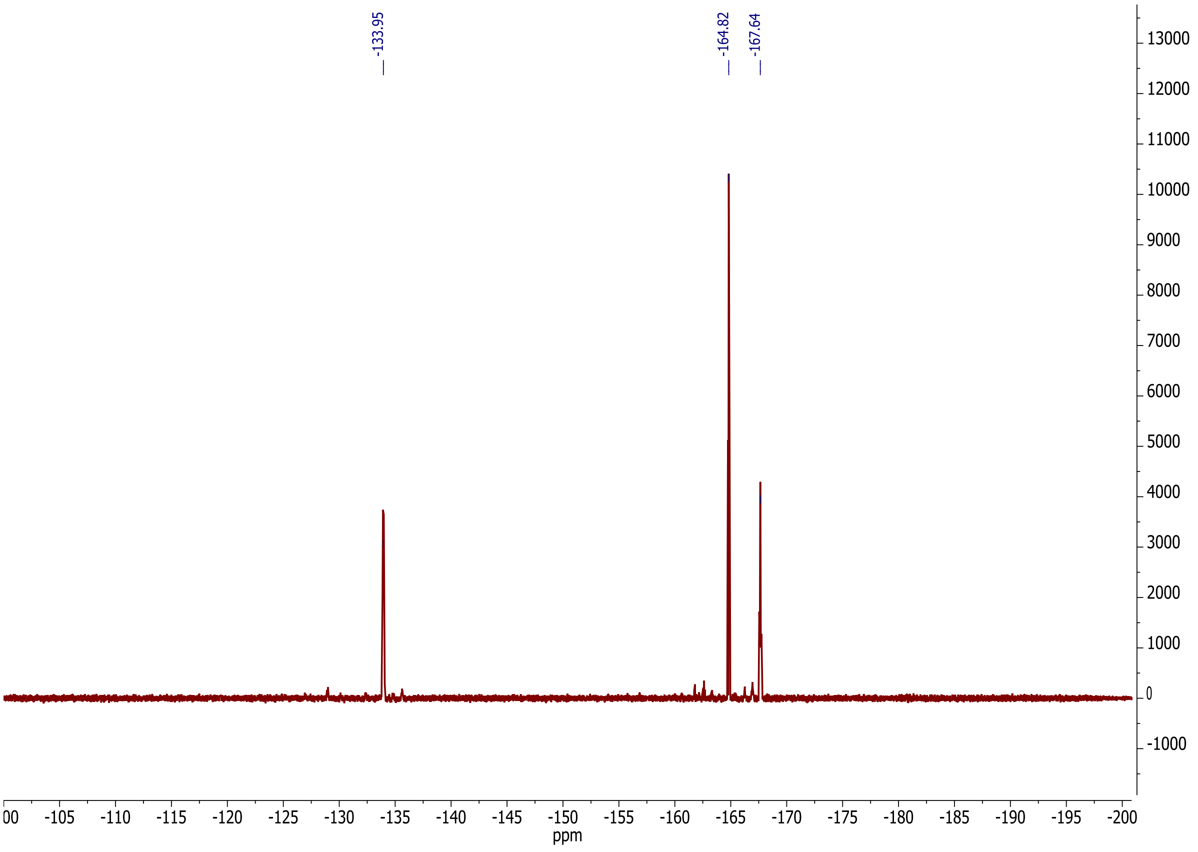

Figure S23. ^{19}F NMR spectrum of complex **3b** in CD_2Cl_2.

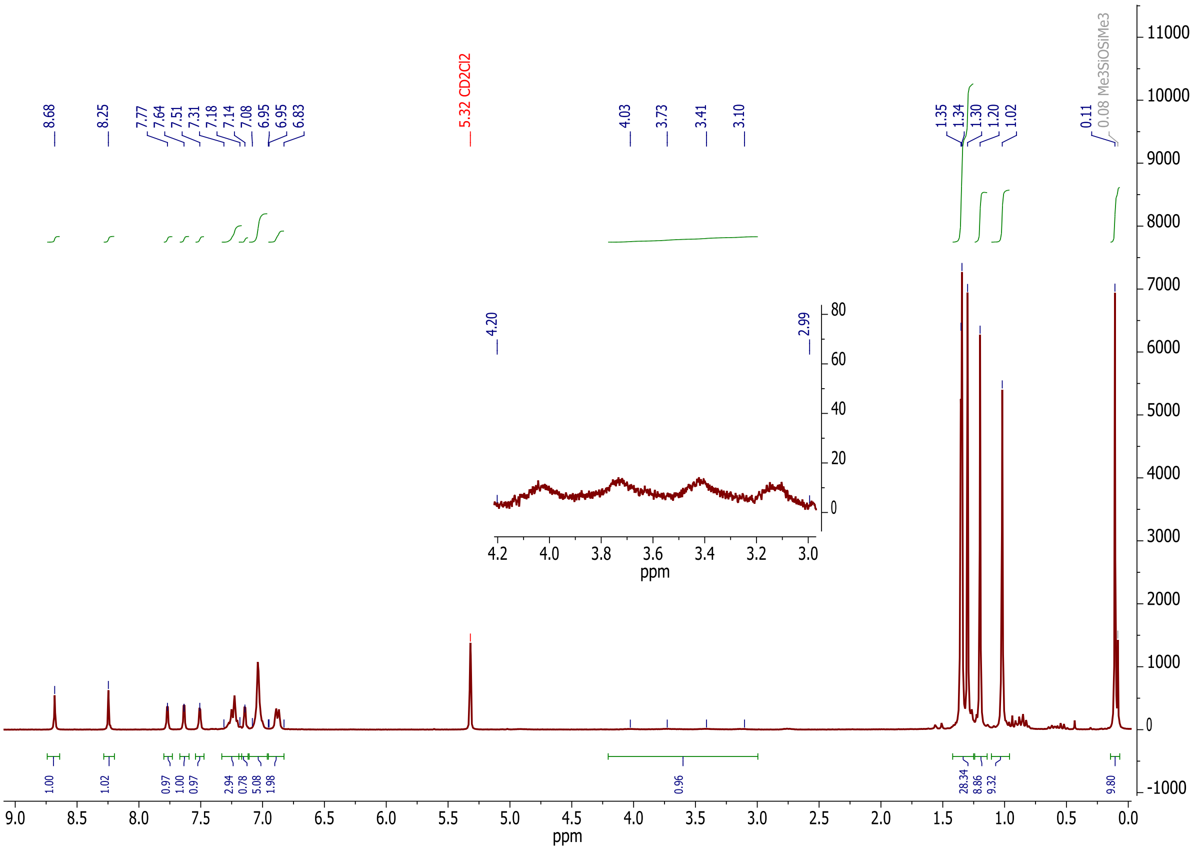

Figure S24. ^{1}H NMR spectrum of complex **3c** in CD_2Cl_2.

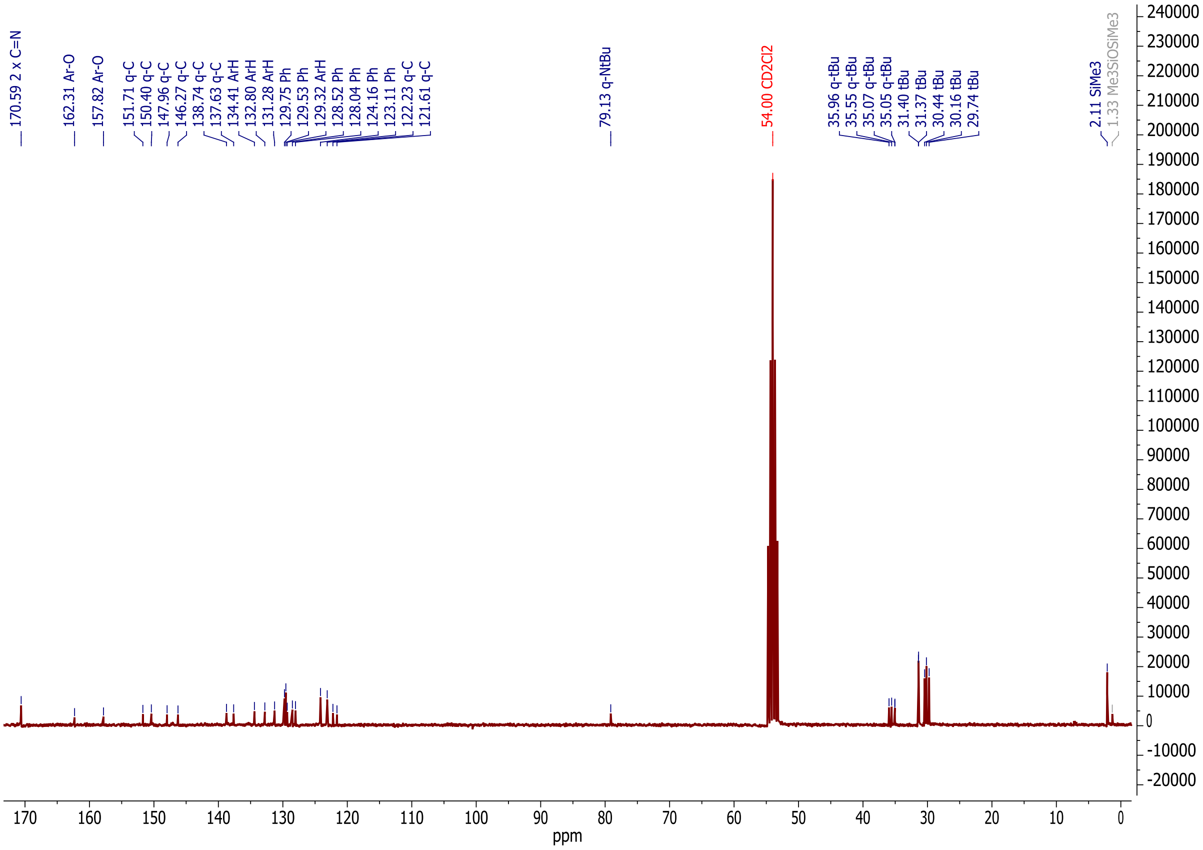

Figure S25. ^{13}C NMR spectrum of complex **3c** in CD_2Cl_2.

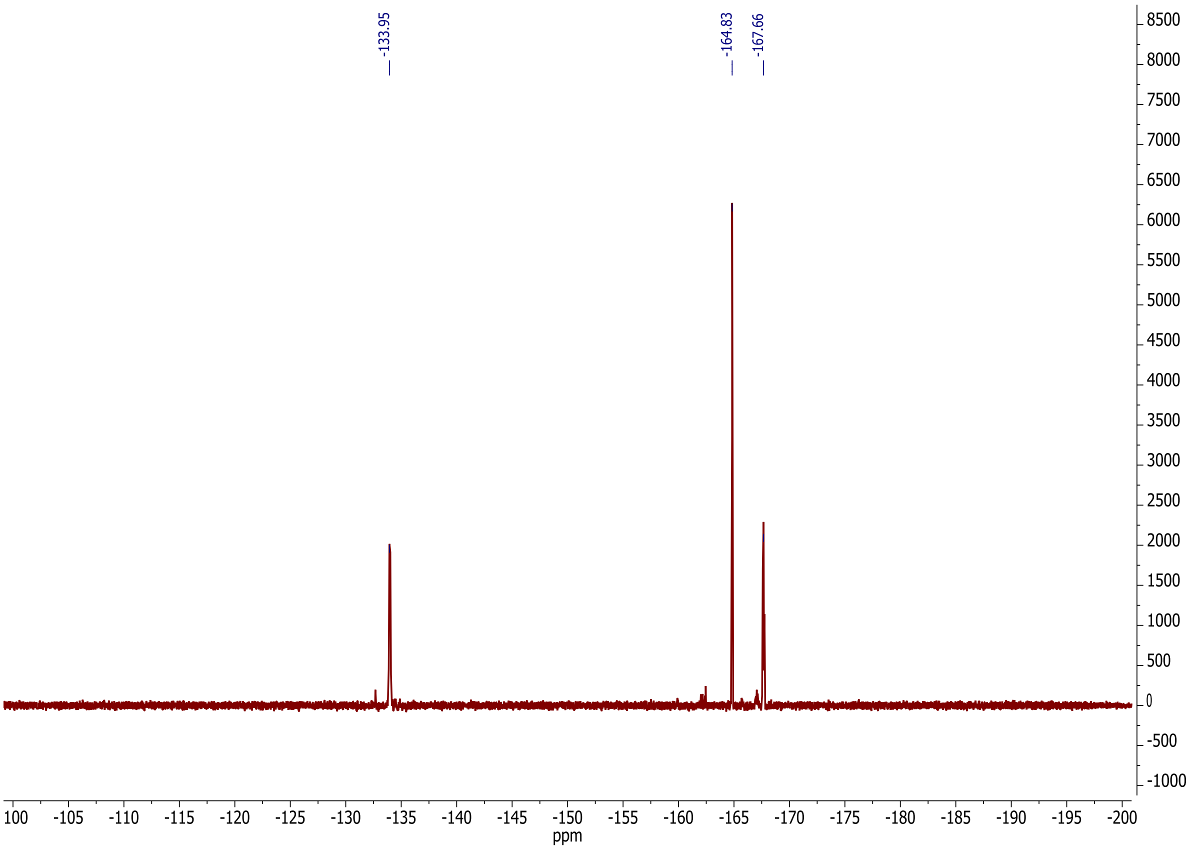

Figure S26. ^{19}F NMR spectrum of complex **3c** in CD_2Cl_2.

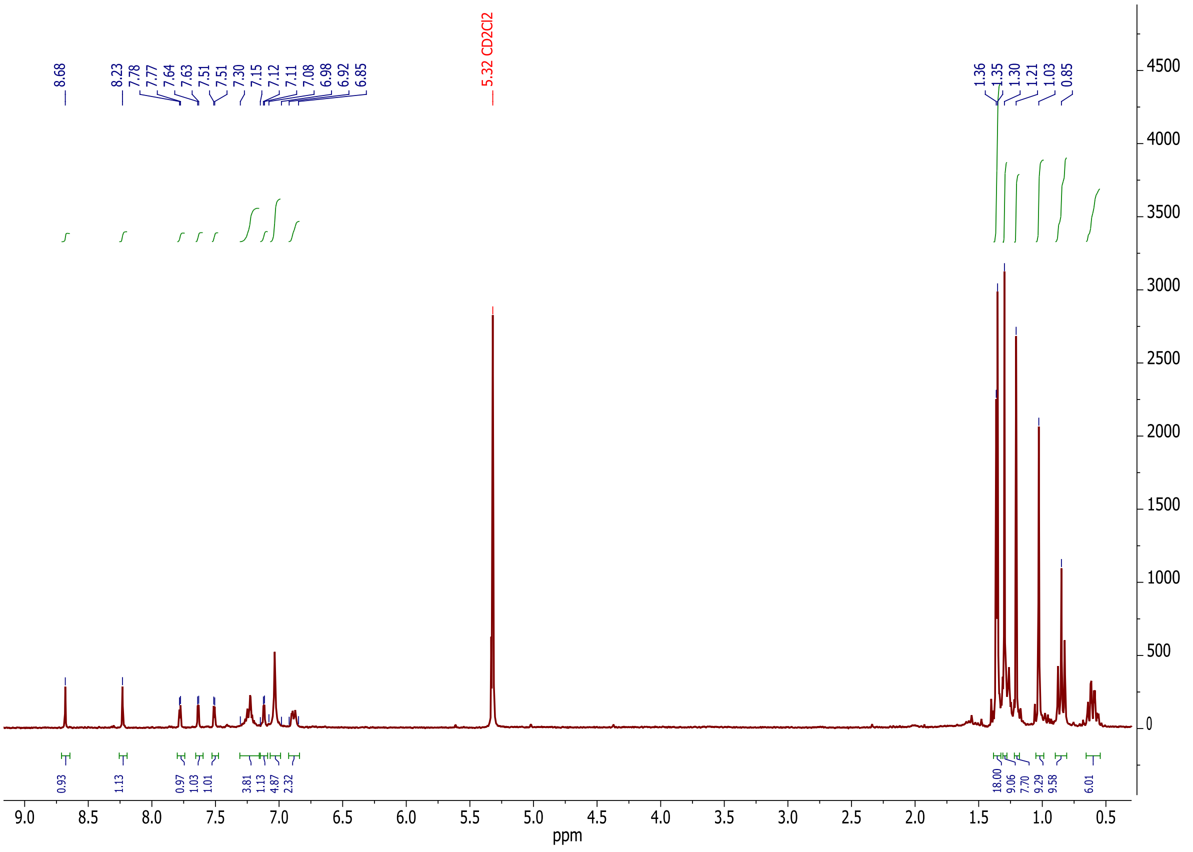

Figure S27. ^{1}H NMR spectrum of complex **4a** in CD_2Cl_2.

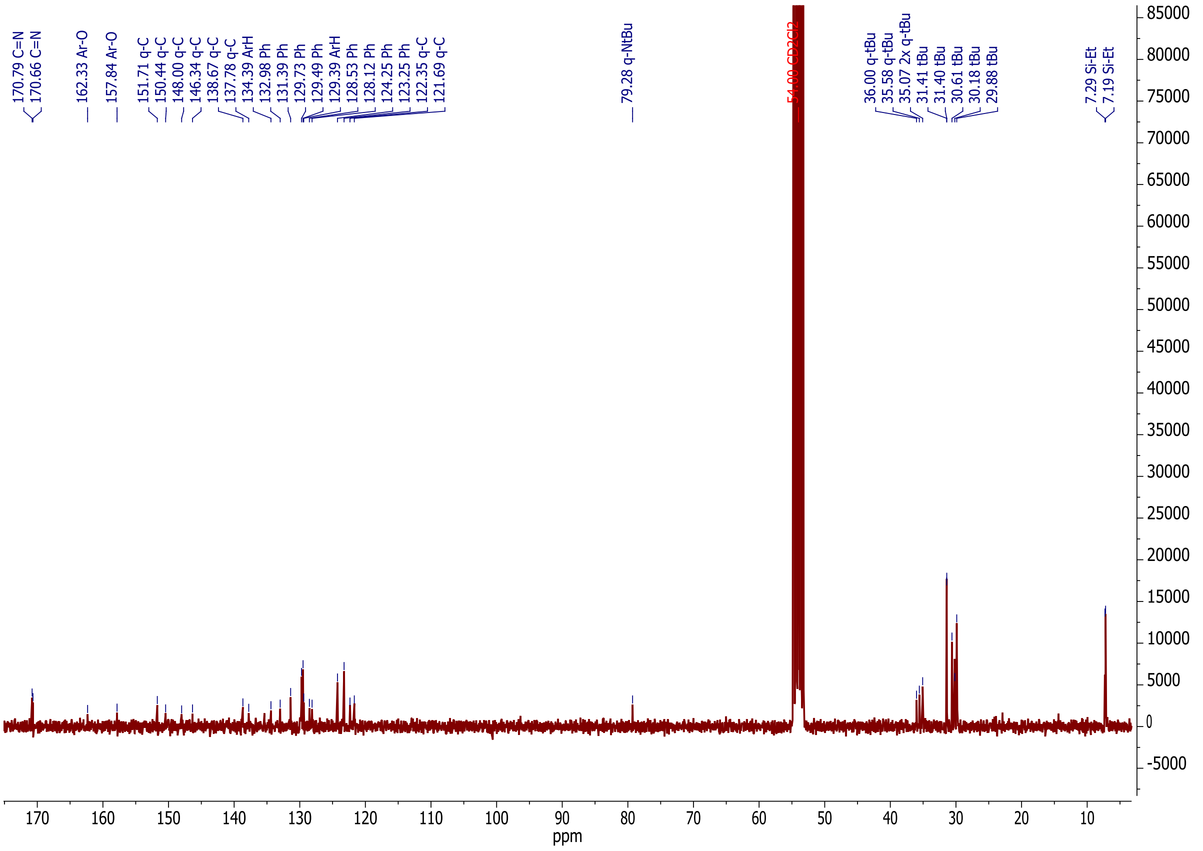

Figure S28. ^{13}C NMR spectrum of complex **4a** in CD_2Cl_2.

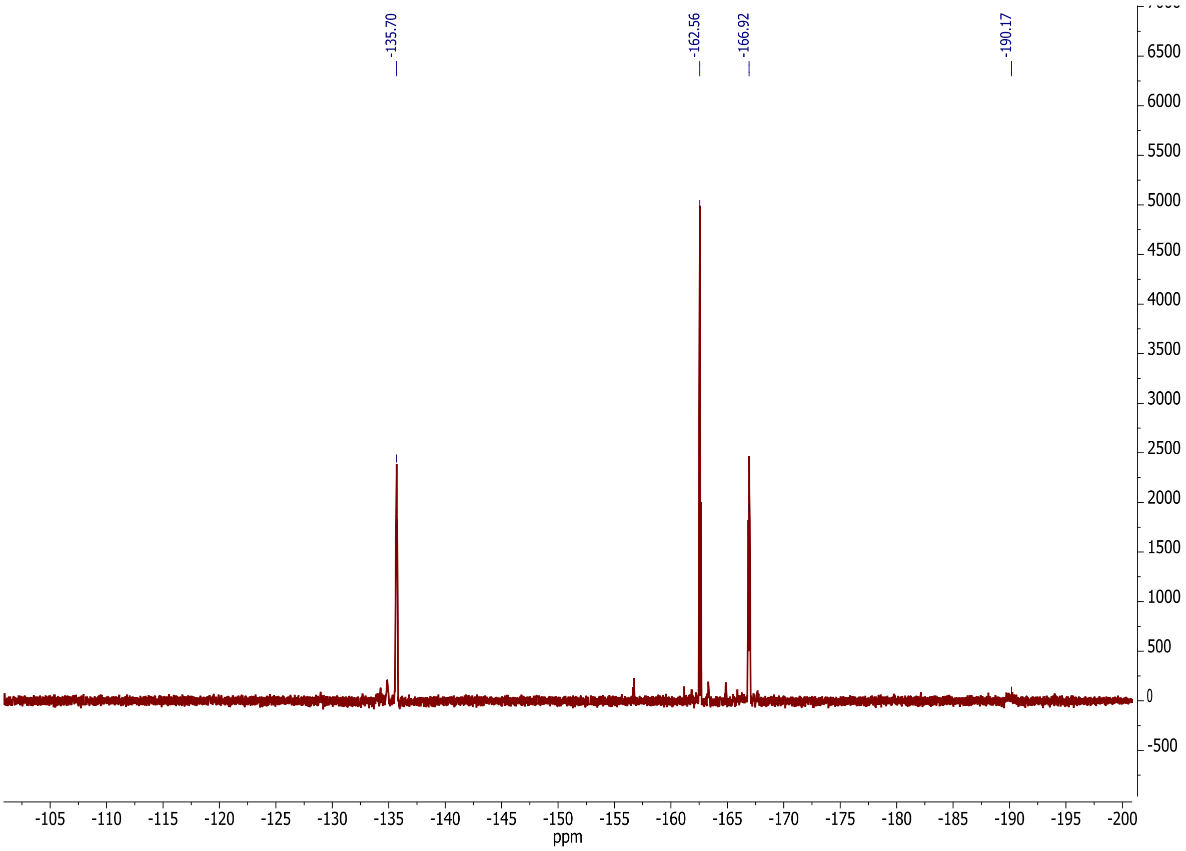

Figure S29. ^{19}F NMR spectrum of complex **4a** in CD_2Cl_2.

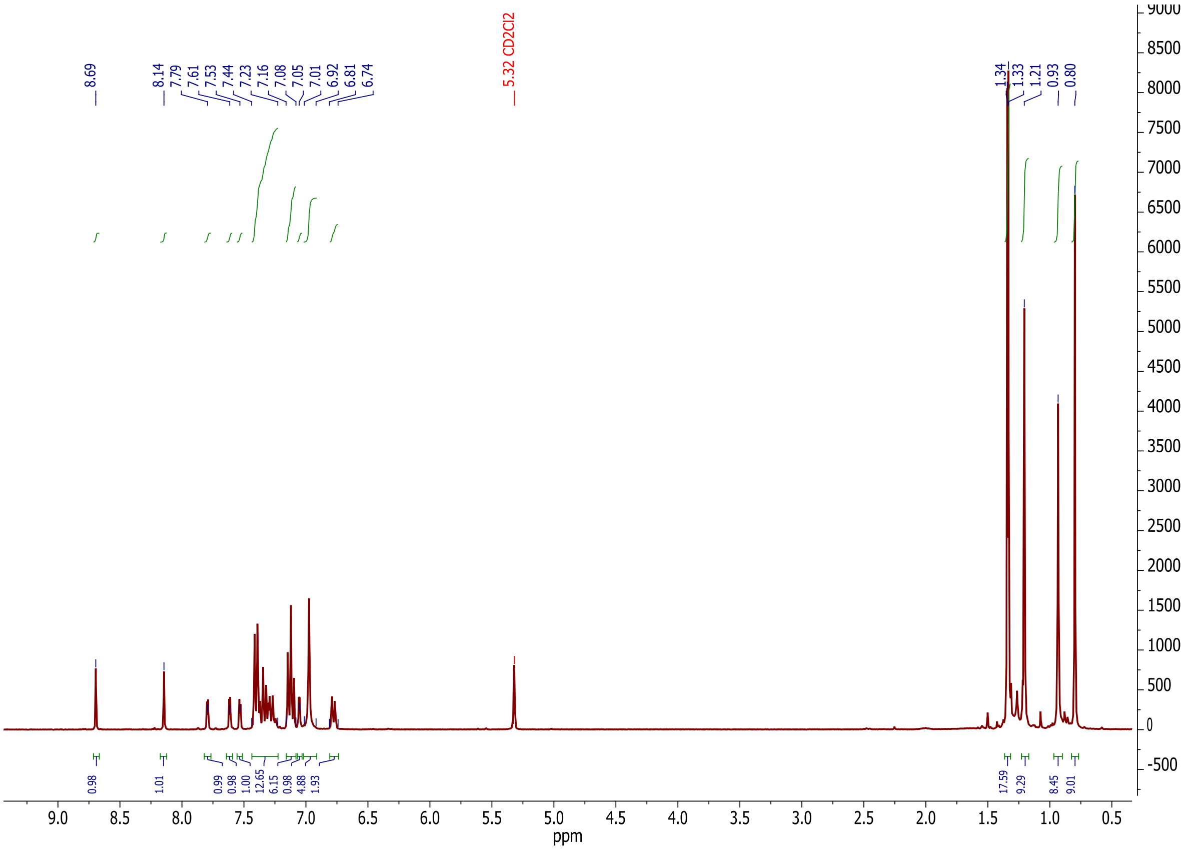

Figure S30. ^{1}H NMR spectrum of complex **4b** in CD_2Cl_2.

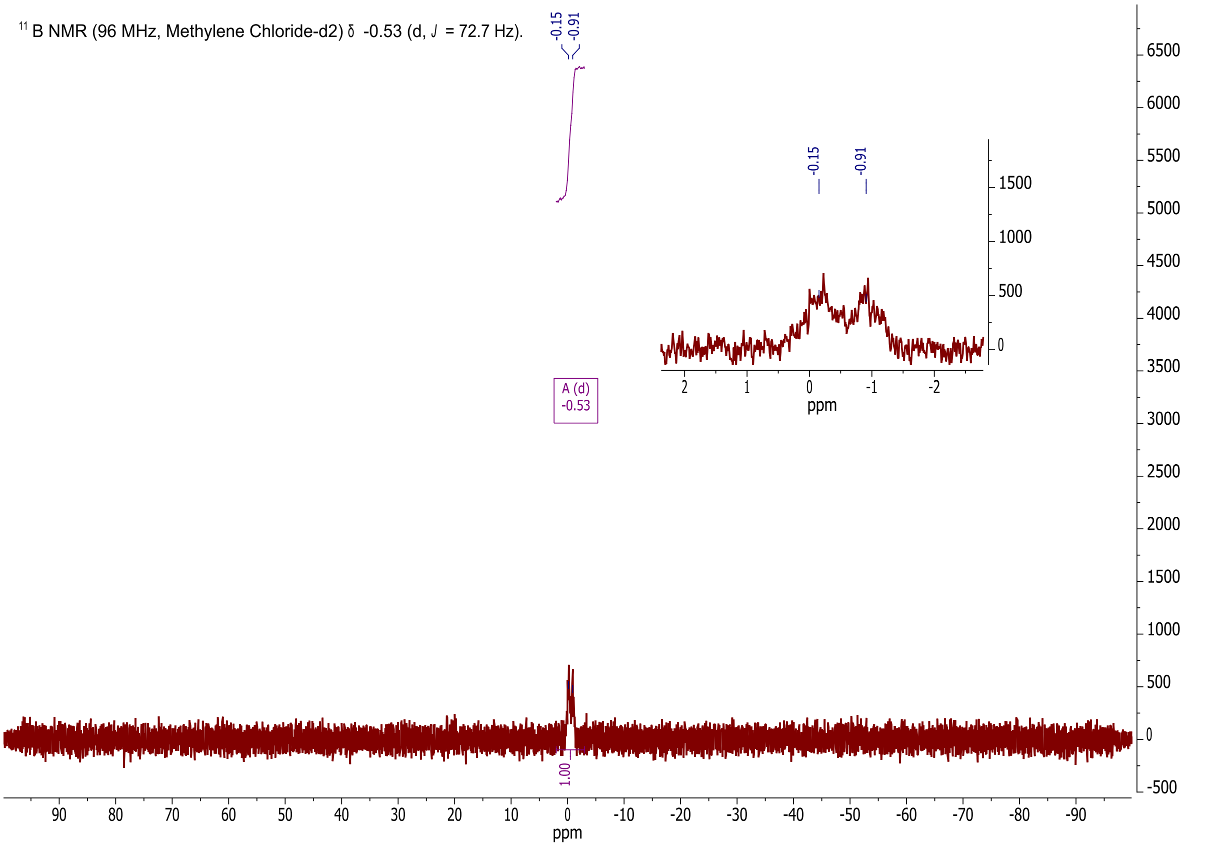

Figure S31. ^{11}B NMR spectrum of complex **4b** in CD_2Cl_2.

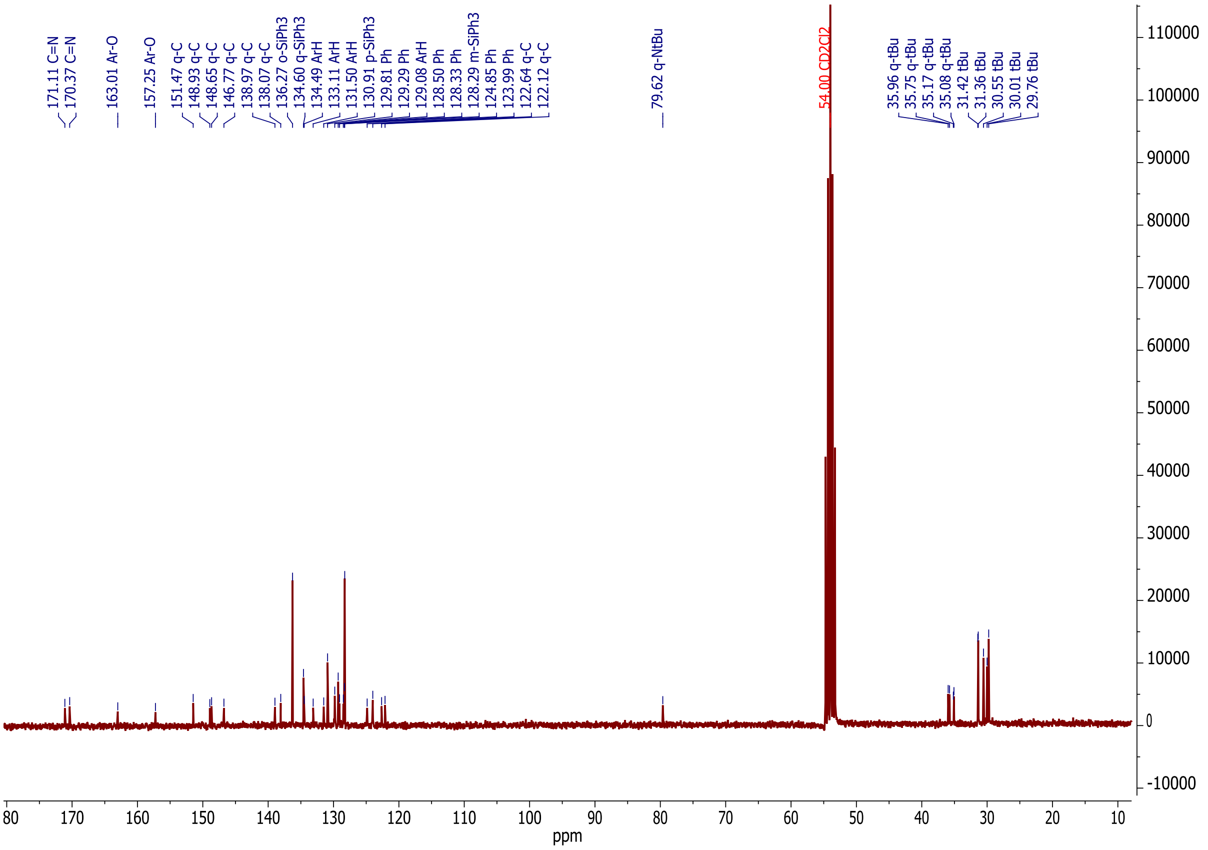

Figure S32. ^{13}C NMR spectrum of complex **4b** in CD_2Cl_2.

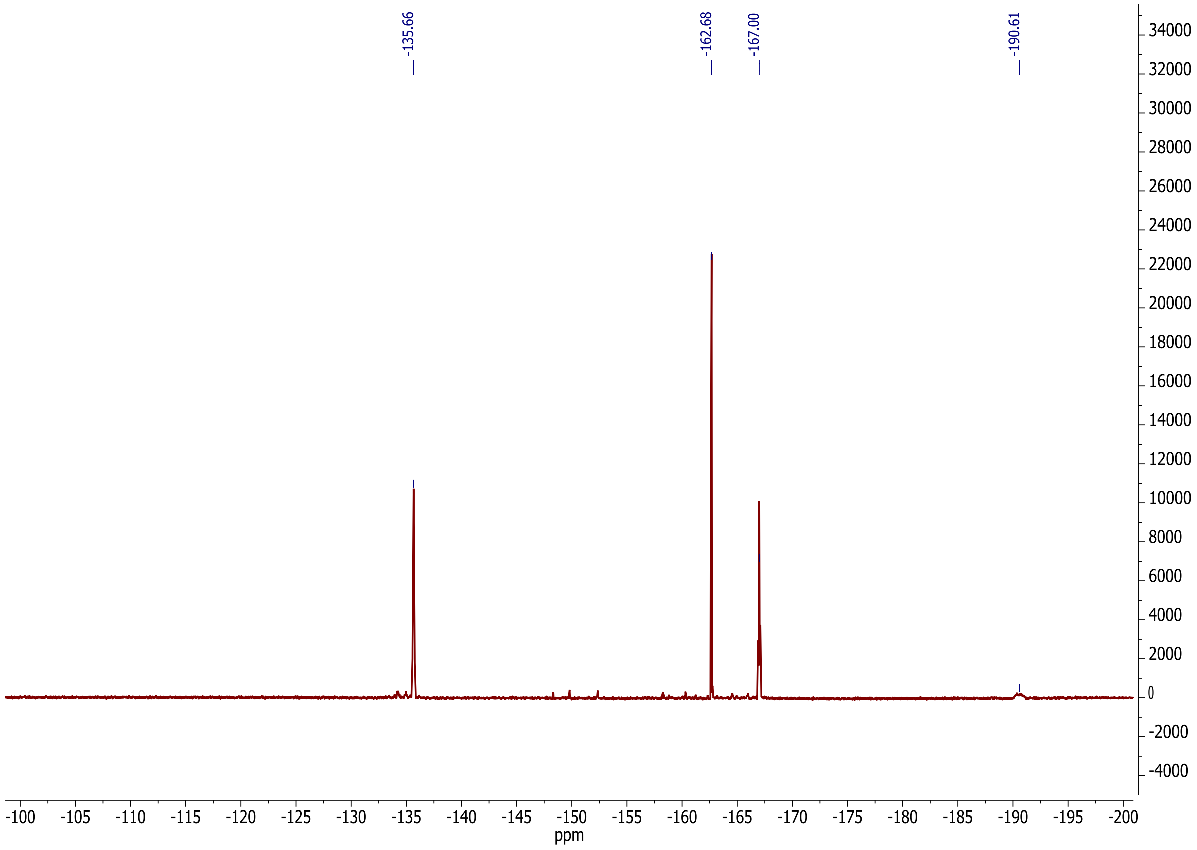

Figure S33. ^{19}F NMR spectrum of complex **4b** in CD_2Cl_2.

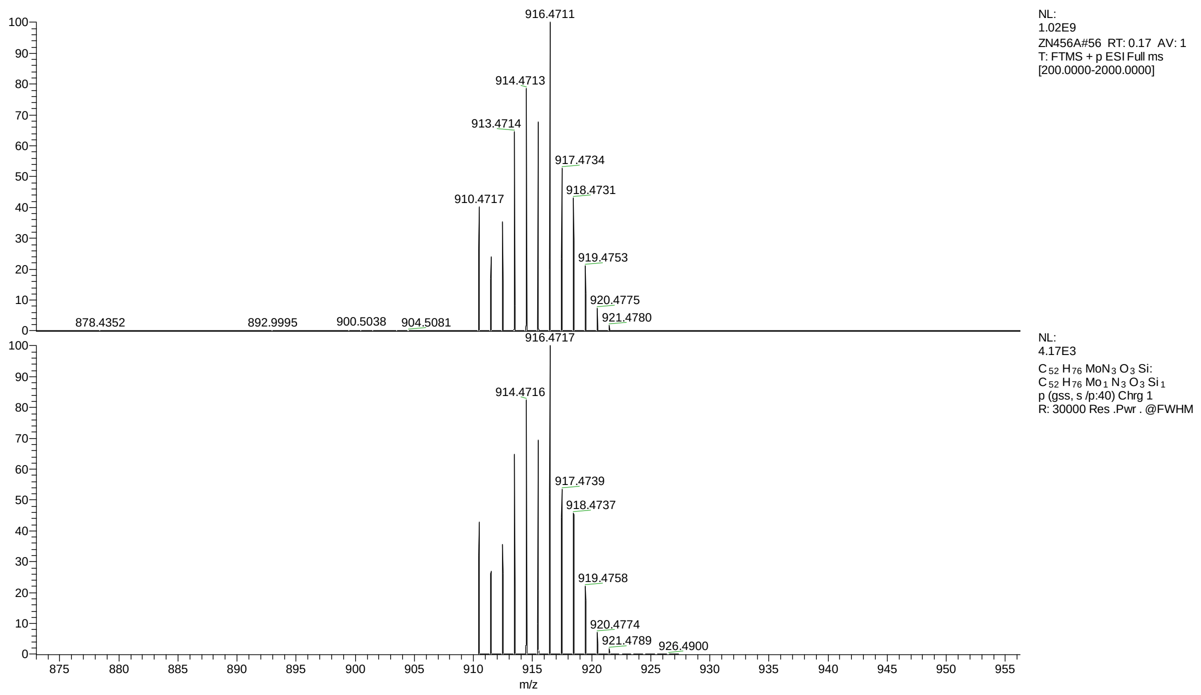

Figure S34. ESI-MS (positive mode) spectrum for **3a**, showing the peak for $[Mo(OSiEt_3)(N\textit{t}Bu)]^+$ with the correct isotope pattern.

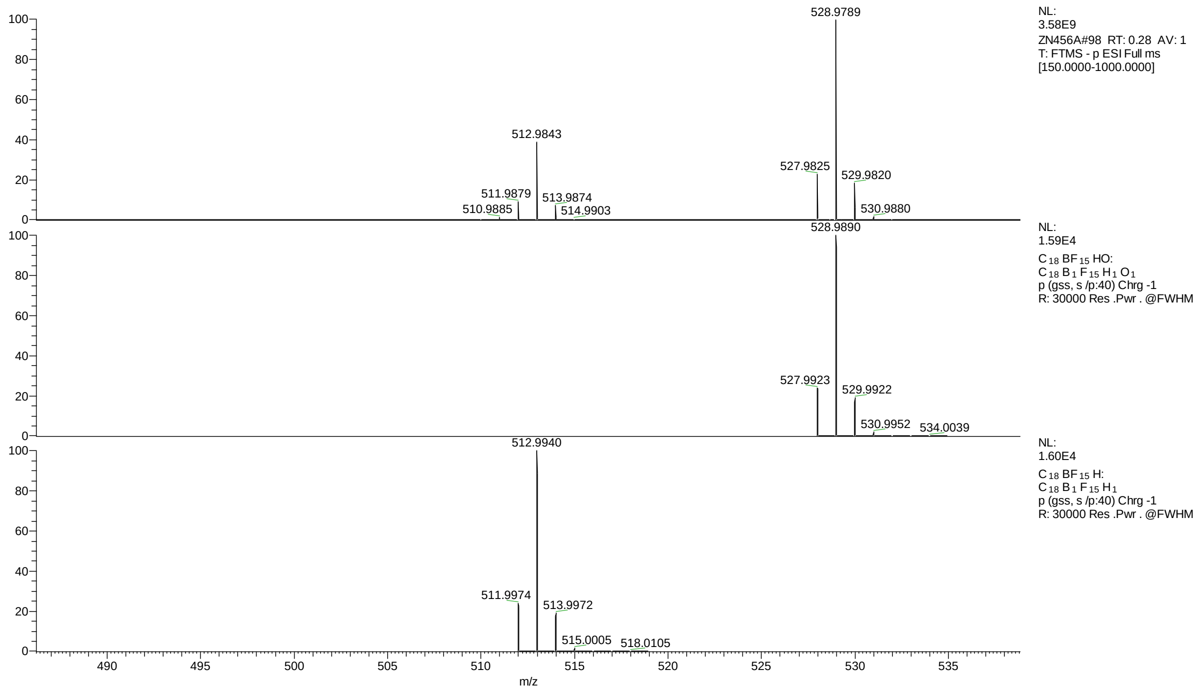

Figure S35. ESI-MS (negative mode) spectrum for **3a**, showing peaks for $[HB(C_6F_5)_3]^-$ and $[HOB(C_6F_5)_3]^-$, the latter presumably originating from a reaction with adventitious water in the sample.

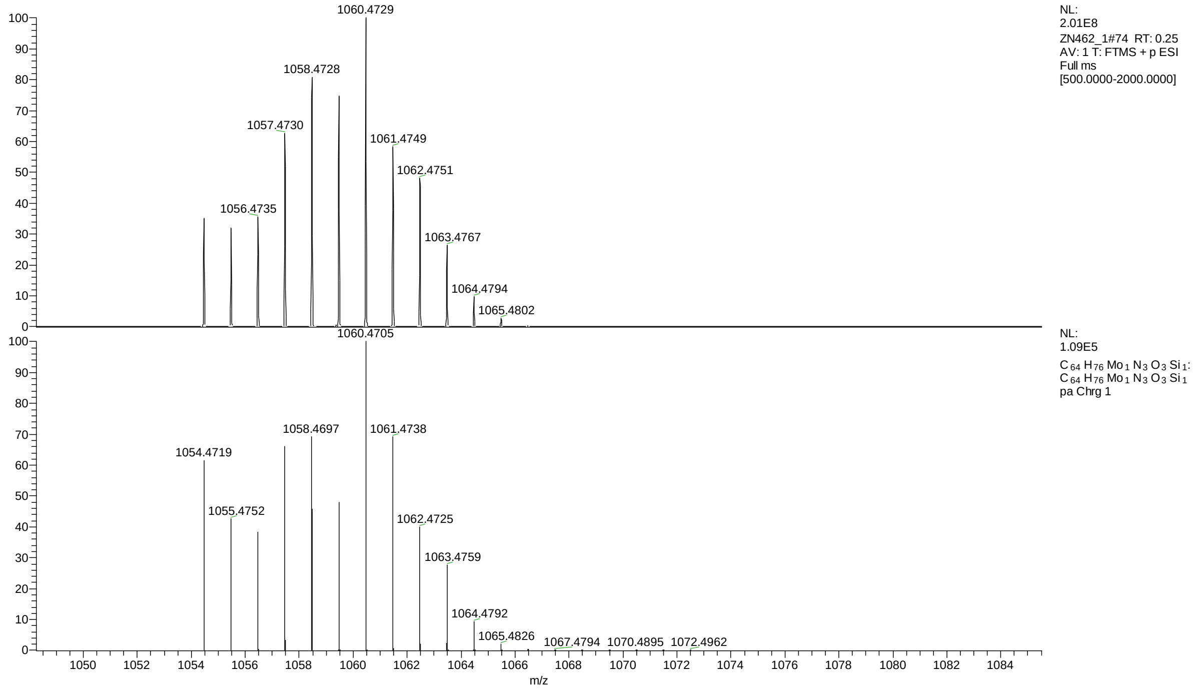

Figure S36. ESI-MS (positive mode) spectrum for **3b**, showing the peak for $[Mo(OSiPh_3)(N\textit{t}Bu)]^+$ with the correct isotope pattern.

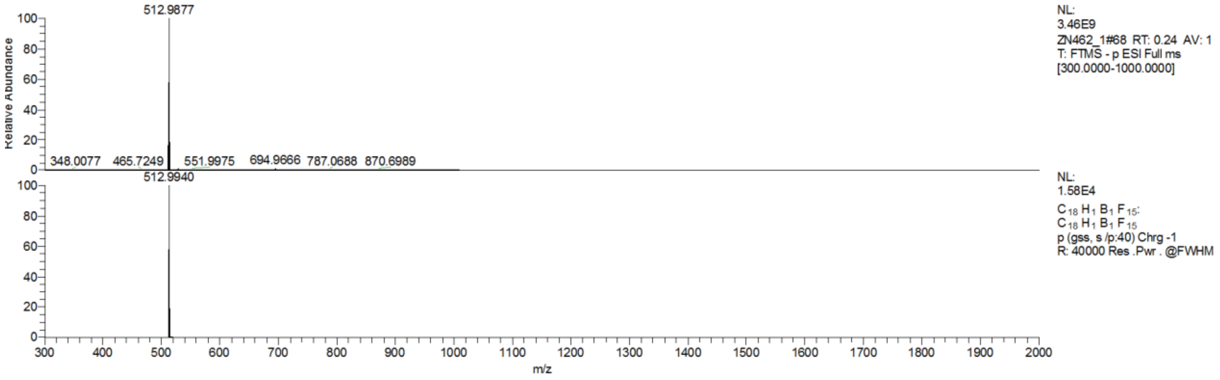

Figure S37. ESI-MS (negative mode) spectrum for **3b**, showing the peak for $[HB(C_6F_5)_3]^-$.

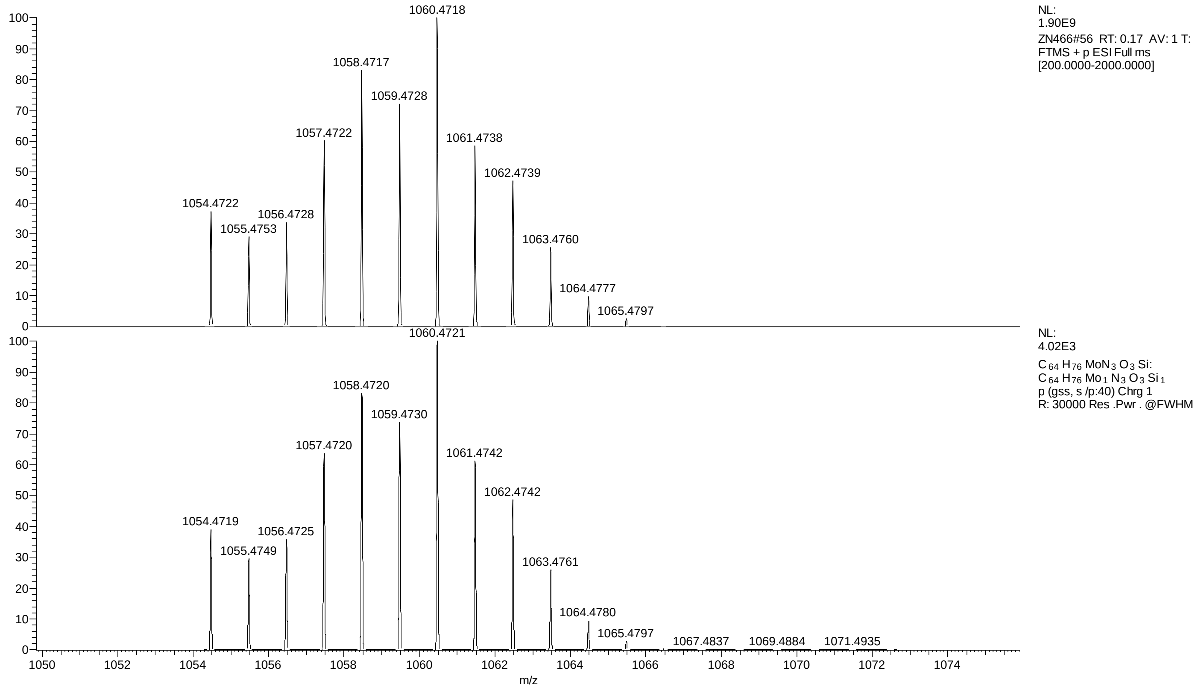

Figure S38. ESI-MS (positive mode) spectrum for **4b**, showing the peak for $[Mo(OSiPh_3)(N\textit{t}Bu)]^+$ with the correct isotope pattern.

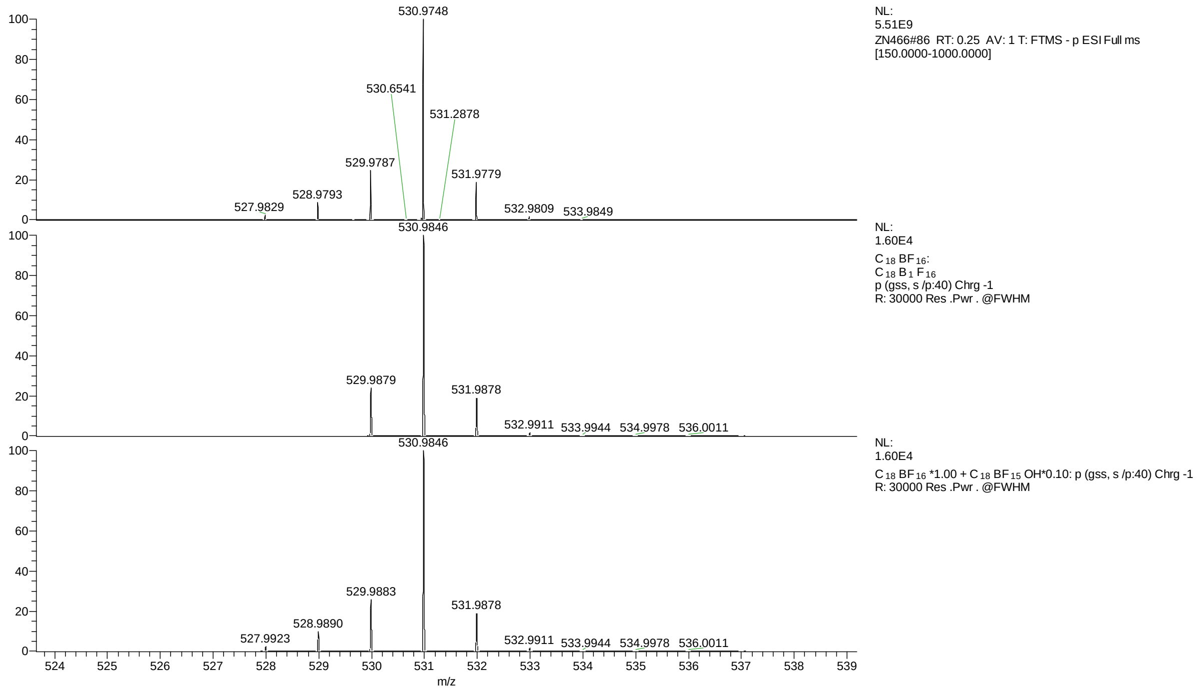

Figure S39. ESI-MS (negative mode) spectrum for **4b**, showing a peak for $[FB(C_6F_5)_3]^-$, accompanied by a small amount of $[HOB(C_6F_5)_3]^-$, the latter presumably originating from a reaction with adventitious water in the sample.

Electrochemistry data of the compounds

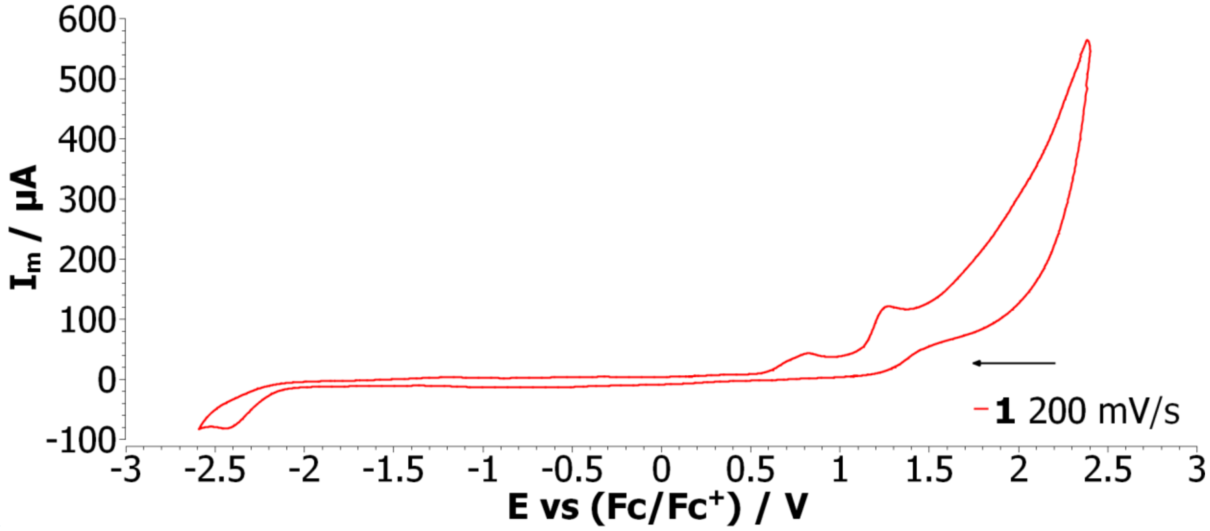

Figure S40. Full sweep-width cyclovoltammogram of complex **1**[1] in MeCN.

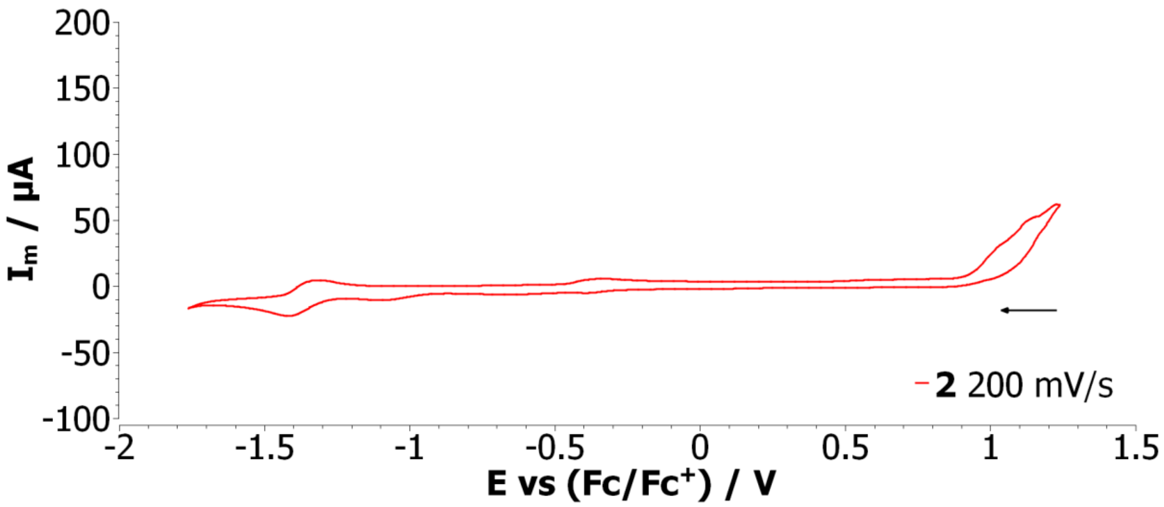

Figure S41. Full sweep-width cyclovoltammogram of complex **2** in CH_2Cl_2.

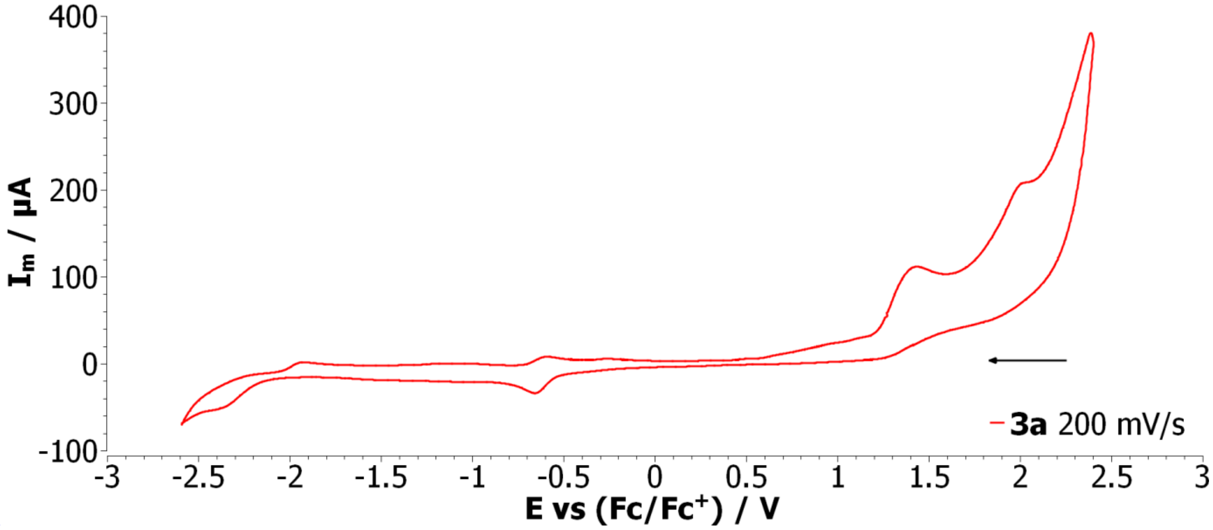

Figure S42. Full sweep-width cyclovoltammogram of complex **3a** in MeCN.

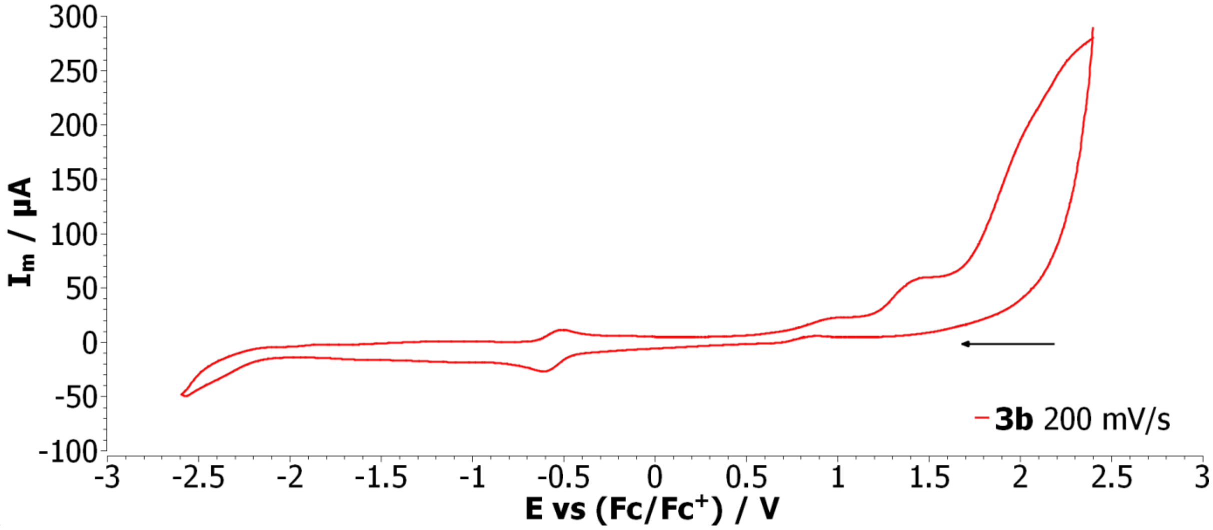

Figure S43. Full sweep-width cyclovoltammogram of complex **3b** in MeCN.

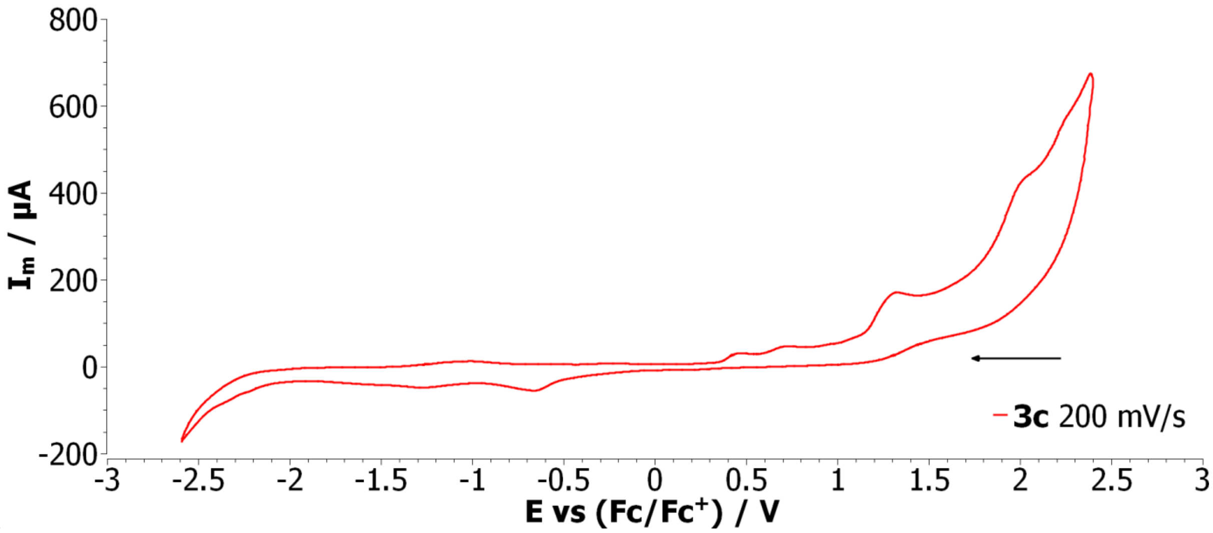

Figure S44. Full sweep-width cyclovoltammogram of complex **3c** in MeCN.

Crystallographic data for 2 and 4b

Crystal structure determination of 2. The structure was solved by direct methods (SHELXS-97)[3] and refined by full-matrix least-squares techniques against F^2 (SHELXL-2014/6).[4] The non-hydrogen atoms of the two complexes of the asymmetric unit were refined with anisotropic displacement parameters without any constraints. The H atoms of the phenyl rings including any adjacent CH=N groups were put at the external bisectors of the C–C–C angles at C–H distances of 0.95Å and common isotropic displacement parameters were refined for the H atoms of the same ring. The H atoms of the *tert*-butyl groups were refined with common isotropic displacement parameters for the H atoms of the same group and idealized geometries with tetrahedral angles, enabling rotation around the C–C bonds, and C–H distances of 0.98Å. For 809 parameters final *R* indices of R_1 = 0.0360 and wR^2 = 0.0900 (GOF = 1.028) were obtained. The largest peak in a difference Fourier map was 0.807eÅ^{-3}.

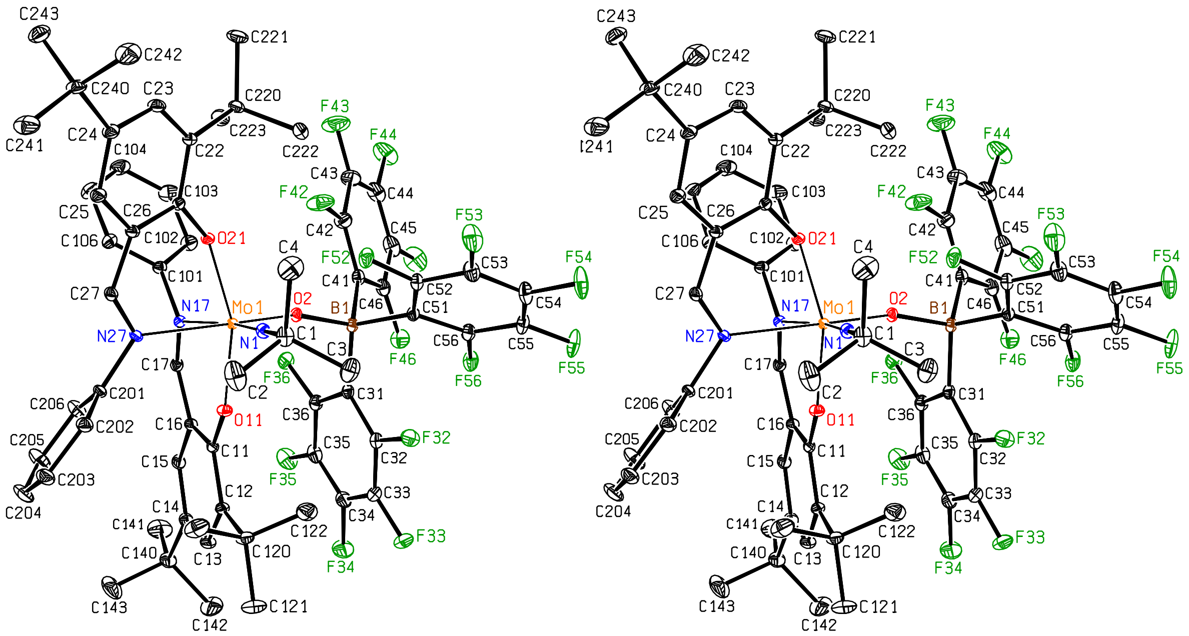

Figure S45. Stereoscopic ORTEP[5] plot of **2** showing the atomic numbering scheme. The probability ellipsoids are drawn at the 50% probability level. The H atoms were omitted for clarity reasons.

Table S1. Crystal data and structure refinement for **2** (CCDC deposition number: 1574332)

**Crystal data**	
Empirical formula	$C_{64}H_{61}BF_{15}MoN_3O_3$
Formula weight	1311.90
Crystal description	block, red
Crystal size	0.32 x 0.19 x 0.18mm
Crystal system, space group	monoclinic, P 2_1/n
Unit cell dimensions: a	12.0188(14)Å
b	26.298(3)Å
c	18.9156(19)Å
β	95.221(5)°
Volume	5953.8(11)$Å^3$
Z	4
Calculated density	1.464Mg/m^3
F(000)	2688
Linear absorption coefficient μ	0.316mm^{-1}
Absorption correction	semi-empirical from equivalents
Max. and min. transmission	1.000 and 0.841
Unit cell determination	2.48° < Θ < 33.67°<br>9805 reflections used at 100K

**Data collection**	
Temperature	100K
Diffractometer	Bruker APEX-II CCD
Radiation source	Incoatec microfocus sealed tube
Radiation and wavelength	MoK_{α}, 0.71073Å
Monochromator	multilayer monochromator
Scan type	ϕ and ω scans
Θ range for data collection	1.87 to 33.00°
Reflections collected / unique	82751 / 22432
Significant unique reflections	17789 with I > 2 σ(I)
R(int), R(sigma)	0.0490, 0.0477
Completeness to Θ = 33.0°	100.0%

**Refinement**	
Refinement method	Full-matrix least-squares on F^2
Data / parameters / restraints	22432 / 809 / 0
Goodness-of-fit on F^2	1.028
Final R indices [I > 2σ(I)]	R1 = 0.0360, wR2 = 0.0831
R indices (all data)	R1 = 0.0515, wR2 = 0.0900
Extinction expression	none
Weighting scheme	$w = 1/[\sigma^2(F_o^2)+(aP)^2+bP]$ where $P = (F_o^2+2F_c^2)/3$
Weighting scheme parameters a, b	0.0357, 2.7963
Largest Δ/σ in last cycle	0.002
Largest difference peak and hole	0.807 and -0.546$e/Å^3$
Structure Solution Program	SHELXS-97 (Sheldrick, 2008)
Structure Refinement Program	SHELXL-2014/6 (Sheldrick, 2015)

Table S2. Selected bond lengths [Å] and angles [°] for **2**.

Mo(1)–N(1)	1.7259(12)
Mo(1)–O(2)	1.8221(9)
Mo(1)–O(11)	1.9583(10)
Mo(1)–O(21)	1.9570(9)
Mo(1)–N(17)	2.3140(12)
Mo(1)–N(27)	2.2957(11)
O(2)–B(1)	1.5002(16)
O(11)–Mo(1)–O(21)	158.63(4)
N(1)–Mo(1)–N(17)	170.36(5)
O(2)–Mo(1)–N(27)	165.81(4)
B(1)–O(2)–Mo(1)	162.72(9)
C(1)–N(1)–Mo(1)	175.08(10)
C(11)–O(11)–Mo(1)	138.36(9)
C(17)–N(17)–C(101)	115.06(11)
C(17)–N(17)–Mo(1)	123.44(9)
C(101)–N(17)–Mo(1)	120.37(8)
C(21)–O(21)–Mo(1)	134.49(8)
C(27)–N(27)–C(201)	112.55(11)
C(27)–N(27)–Mo(1)	121.04(9)
C(201)–N(27)–Mo(1)	125.69(8)

Crystal structure determination of 4b. The structure was solved by direct methods (SHELXS-97)[3] and refined by full-matrix least-squares techniques against F^2 (SHELXL-2014/6).[4] The n-pentane molecules are disordered over two orientations and were refined with site occupation factors of 0.5. The C–C bonds in these solvent molecules were restrained to 1.53Å and the same anisotropic displacement parameters were used for the C atoms. The other non-hydrogen atoms were refined with anisotropic displacement parameters without any constraints. The H atoms of the aromatic rings as well as those of the imino groups were put at the external bisectors of the C–C–C angles at C–H distances of 0.95Å and common isotropic displacement parameters were refined for the H atoms of the same ring including the H atom of the adjacent imino group. The H atoms of the *tert*-butyl groups were refined with common isotropic displacement parameters for the H atoms of the same group and idealized geometries with tetrahedral angles, enabling rotation around the C–C bonds, and C–H distances of 0.98Å. The H atoms of the solvent molecules were included with idealized geometries and their isotropic displacement parameters fixed to 1.2 times U_{eq} of the C atom they are bonded to. For 1027 parameters final *R* indices of R1 = 0.0434 and wR^2 = 0.1283 (GOF = 1.038) were obtained. The largest peak in a difference Fourier map was 0.669eÅ^{-3}.

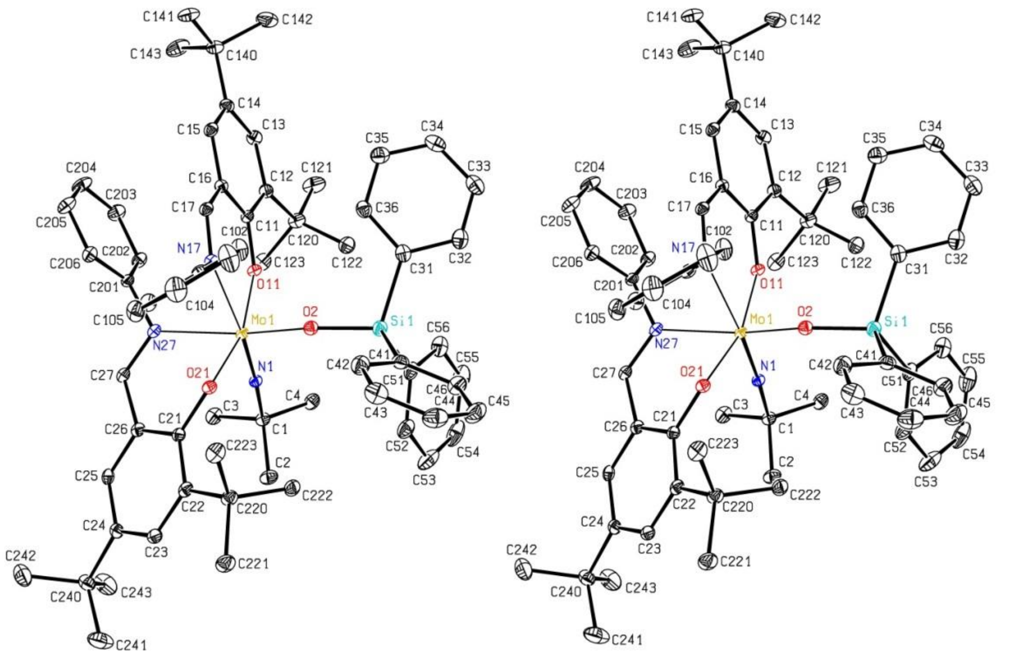

Figure S46. Stereoscopic ORTEP[5] plot of the complex cation found in **4b** showing the atomic numbering scheme. The probability ellipsoids are drawn at the 30% probability level. The H atoms were omitted for clarity reasons.

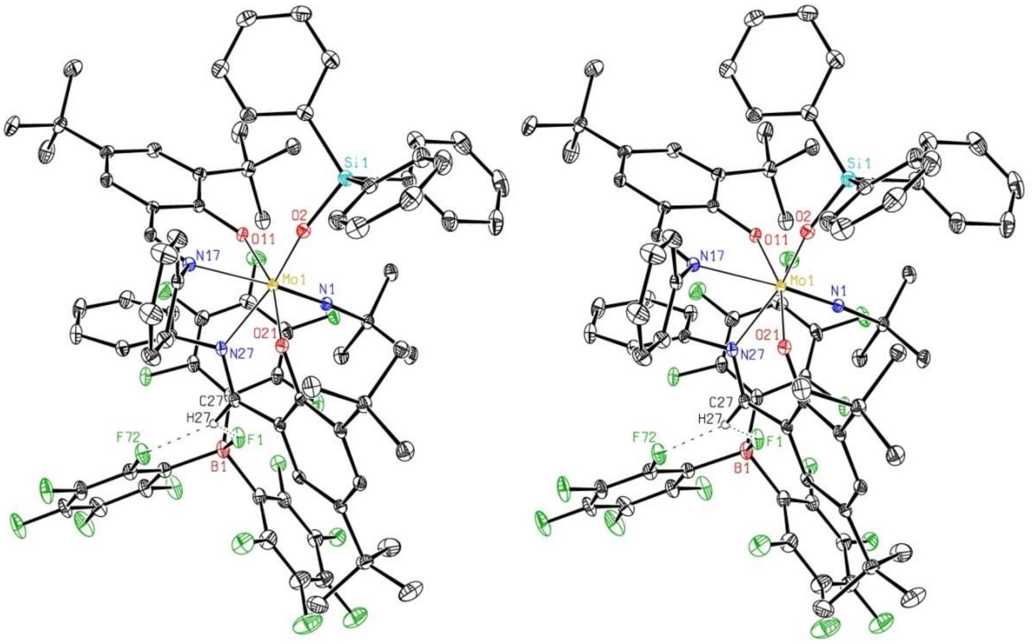

Figure S47. Stereoscopic ORTEP[5] plot of **4b** showing the atomic numbering scheme. The probability ellipsoids are drawn at the 30% probability level. H27 is drawn with an arbitrary radius; the other H atoms were omitted for clarity.

Table S3. Crystal data and structure refinement for **4b** (CCDC deposition number: 1574333)

Crystal data

Empirical formula		$C_{64}H_{76}MoN_3O_3Si^+ \; C_{18}BF_{16}^- \cdot C_5H_{12}$
Formula weight		1662.44
Crystal description		plate, amber
Crystal size		0.30 x 0.26 x 0.03mm
Crystal system, space group		monoclinic, P 2_1/c
Unit cell dimensions:	a	13.3781(10)Å
	b	36.228(3)Å
	c	18.4909(13)Å
	β	97.748(3)°
Volume		8880.0(12)$Å^3$
Z		4
Calculated density		1.243Mg/m^3
F(000)		3440
Linear absorption coefficient μ		0.241mm^{-1}
Absorption correction		semi-empirical from equivalents
Max. and min. transmission		1.000 and 0.839
Unit cell determination		2.31° < Θ < 25.52°
		9947 reflections used at 100K

Data collection

Temperature	100K
Diffractometer	Bruker APEX-II CCD
Radiation source	Incoatec microfocus sealed tube
Radiation and wavelength	MoK_α, 0.71073Å
Monochromator	multilayer monochromator
Scan type	ϕ and ω scans
Θ range for data collection	1.86 to 25.00°
Reflections collected / unique	50857 / 15605
Significant unique reflections	11678 with I > 2σ(I)
R(int), R(sigma)	0.0368, 0.0601
Completeness to Θ = 25.0°	99.9%

Refinement

Refinement method	Full-matrix least-squares on F^2
Data / parameters / restraints	15605 / 1027 / 14
Goodness-of-fit on F^2	1.038
Final R indices [I > 2σ(I)]	R1 = 0.0434, wR2 = 0.1170
R indices (all data)	R1 = 0.0663, wR2 = 0.1283
Extinction expression	none
Weighting scheme	$w = 1/[\sigma^2(F_o^2)+(aP)^2+bP]$ where $P = (F_o^2+2F_c^2)/3$
Weighting scheme parameters a, b	0.0691, 1.9520
Largest Δ/σ in last cycle	0.001
Largest difference peak and hole	0.669 and -0.404e/$Å^3$
Structure Solution Program	SHELXS-97 (Sheldrick, 2008)
Structure Refinement Program	SHELXL-2014/6 (Sheldrick, 2015)

Table S4. Selected bond lengths [Å] and angles [°] for **4b**.

Mo(1)–N(1)	1.715(2)
Mo(1)–O(2)	1.8975(19)
Mo(1)–O(11)	1.9391(18)
Mo(1)–O(21)	1.9365(18)
Mo(1)–N(17)	2.315(2)
Mo(1)–N(27)	2.214(2)
N(1)–C(1)	1.470(4)
O(2)–Si(1)	1.630(2)
O(11)–C(11)	1.344(3)
C(17)–N(17)	1.291(3)
O(21)–C(21)	1.354(3)
C(27)–N(27)	1.289(4)
B(1)–F(1)	1.435(3)
B(1)–C(61)	1.646(5)
B(1)–C(71)	1.648(4)
B(1)–C(81)	1.649(5)
O(11)–Mo(1)–O(21)	163.05(8)
N(1)–Mo(1)–N(17)	170.64(9)
O(2)–Mo(1)–N(27)	163.49(8)
C(1)–N(1)–Mo(1)	175.60(19)
Si(1)–O(2)–Mo(1)	155.54(12)
C(11)–O(11)–Mo(1)	143.05(17)
C(17)–N(17)–C(101)	114.2(2)
C(17)–N(17)–Mo(1)	125.59(18)
C(101)–N(17)–Mo(1)	120.03(17)
C(21)–O(21)–Mo(1)	125.99(16)
C(27)–N(27)–C(201)	115.8(2)
C(27)-N(27)–Mo(1)	121.15(17)
C(201)–N(27)–Mo(1)	123.04(18)

References

[1] N. Zwettler, N. Grover, F. Belaj, K. Kirchner, N. C. Mösch-Zanetti, *Inorg. Chem.* **2017**, *56*(17), 10147–10150.

[2] M. H. Holthausen, T. Mahdi, C. Schlepphorst, L. J. Hounjet, J. J. Weigand, D. W. Stephan, *Chem. Commun.* **2014**, *50*(70), 10038–10040.

[3] G. M. Sheldrick, *Acta Crystallogr, A* **2008**, *64*(Pt 1), 112–122.

[4] G. M. Sheldrick, *Acta Crystallogr, C* **2015**, *71*(Pt 1), 3–8.

[5] C. K. Johnson, *ORTEP. Report ORNL-3794,* Tennessee, USA, **1965**.
